# On-Site Detection of Neonicotinoid Pesticides Using Functionalized Gold Nanoparticles and Halogen Bonding

**DOI:** 10.1021/acsanm.3c00618

**Published:** 2023-04-24

**Authors:** Molly M. Sherard, Quang Minh Dang, Sophia C. Reiff, Jeffrey H. Simpson, Michael C. Leopold

**Affiliations:** Department of Chemistry, Gottwald Center for the Sciences, University of Richmond, Richmond, Virginia 23173, United States

**Keywords:** neonicotinoid, pesticides, gold nanoparticles, halogen bonding, imidacloprid, nitenpyram, aggregation

## Abstract

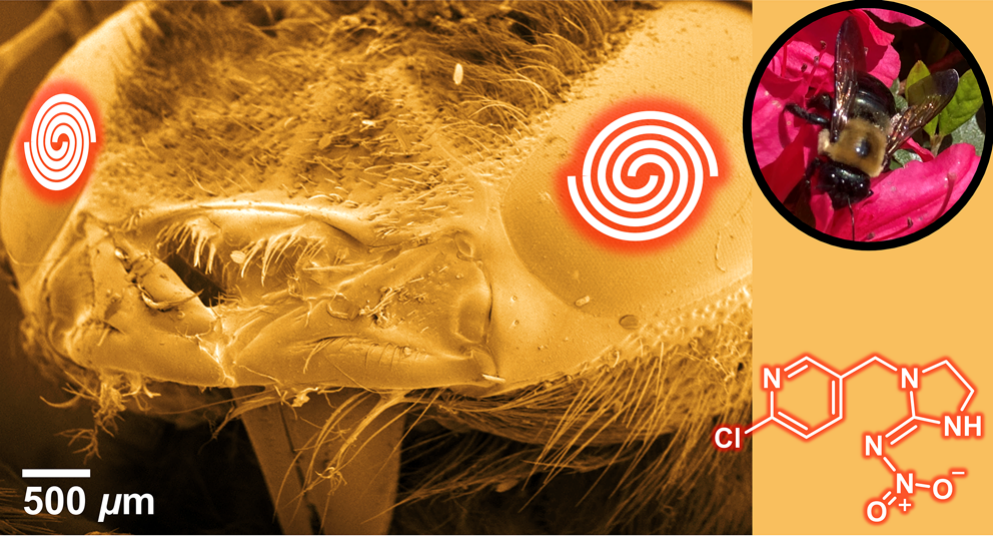

Neonicotinoid (NN) pesticides have emerged globally as one of the most widely used agricultural tools for protecting crops from pest damage and boosting food production. Unfortunately, some NN compounds, such as extensively employed imidacloprid-based pesticides, have also been identified as likely endangering critical pollinating insects like honey bees. To this end, NN pesticides pose a potential threat to world food supplies. As more countries restrict or prohibit the use of NN pesticides, tools are needed to effectively and quickly identify the presence of NN compounds like imidacloprid on site (e.g., in storage areas on farms or pesticide distribution warehouses). This study represents a proof-of-concept where the colloidal properties of specifically modified gold nanoparticles (Au-NPs) able to engage in the rare intermolecular interaction of halogen bonding (XB) can result in the detection of certain NN compounds. Density functional theory and diffusion-ordered NMR spectroscopy (DOSY NMR) are used to explore the fundamental XB interactions between strong XB-donor structures and NN compounds, with the latter found to possess multiple XB-acceptor binding sites. A fundamental understanding of these XB interactions allows for the functionalization of alkanethiolate-stabilized Au-NPs, known as monolayer-protected gold clusters (MPCs), with XB-donor capability (*f*-MPCs). In the presence of certain NN compounds such as imidacloprid, the *f*-MPCs subsequently exhibit visual XB-induced aggregation that is also measured with absorption (UV–vis) spectroscopy and verified with transmission electron microscopy (TEM) imaging. The demonstrated *f*-MPC-aggregation detection scheme has a number of favorable attributes, including quickly reporting the presence of the NN target, requiring only micrograms of suspect material, and being highly selective for imidacloprid, the most prevalent and most important NN insecticide compound. Requiring no instrumentation, the presented methodology can be envisioned as a simple screening test in which dipping a cotton swab of an unknown powder from a surface in a *f*-MPC solution causes *f*-MPCs to aggregate and yield a preliminary indication of imidacloprid presence.

## 1 Introduction and Background

Pollinating insects remain critical to world-wide food production that is needed to meet the needs of the fast-growing global population. Honey bees (*Apis mellifera*) represent the most important group of pollinating insects with ∼35% of world crop production, an estimated $160 billion industry, dependent on their assistance^1−7^ in maintaining a balanced and healthy ecosystem through pollination of both crop-producing and wild plants.^1^ Food production is also dependent on chemical pesticides, which provide protection of harvested crops from chewing insects (e.g., plant hoppers, beetles, and moths). Most pesticides work systemically, diffusing throughout all parts of the plant tissues, including nectar and pollen, and thus presenting direct exposure to foraging insects that can carry contaminated materials back to their colony hives.^4,8,9^ Governmental authorization of specific pesticide usage is typically preceded by required mortality testing that shows field concentrations of a pesticide are nonlethal to bees.^8^ Despite the recognition of risks to bees and increasing government regulations, the last two decades have seen drastic and continual declines in bee populations across the globe. In the winter of 2007, for example, ∼30% of U.S. beekeepers reported rapid and alarming losses of bee colonies, a phenomenon known as colony collapse disorder (CCD).^6^

Scientists have identified a number of contributing factors to declining bee populations and increasing CCD incidents that include habitat loss, invasive species (e.g., murder hornet), and the use of popular pesticides.^4,10^ Research has already established that repeated exposure to even nonlethal concentrations of pesticides may result in both *direct* adverse effects where pollinating insects’ foraging behaviors, such as learning, memory, and organizational/communication skills, are significantly impaired,^1,8,11^ and *indirect* consequences where exposure is linked to an increased vulnerability to virus infection and intestinal parasites (e.g., *Nosema*).^10^ As of 2021, most research now affirms that the most widely used class of pesticides poses an inherent and unacceptable danger to honey bees, bumble bees, and solitary pollinating bees.^1,4,9,12^

**Scheme 1:**
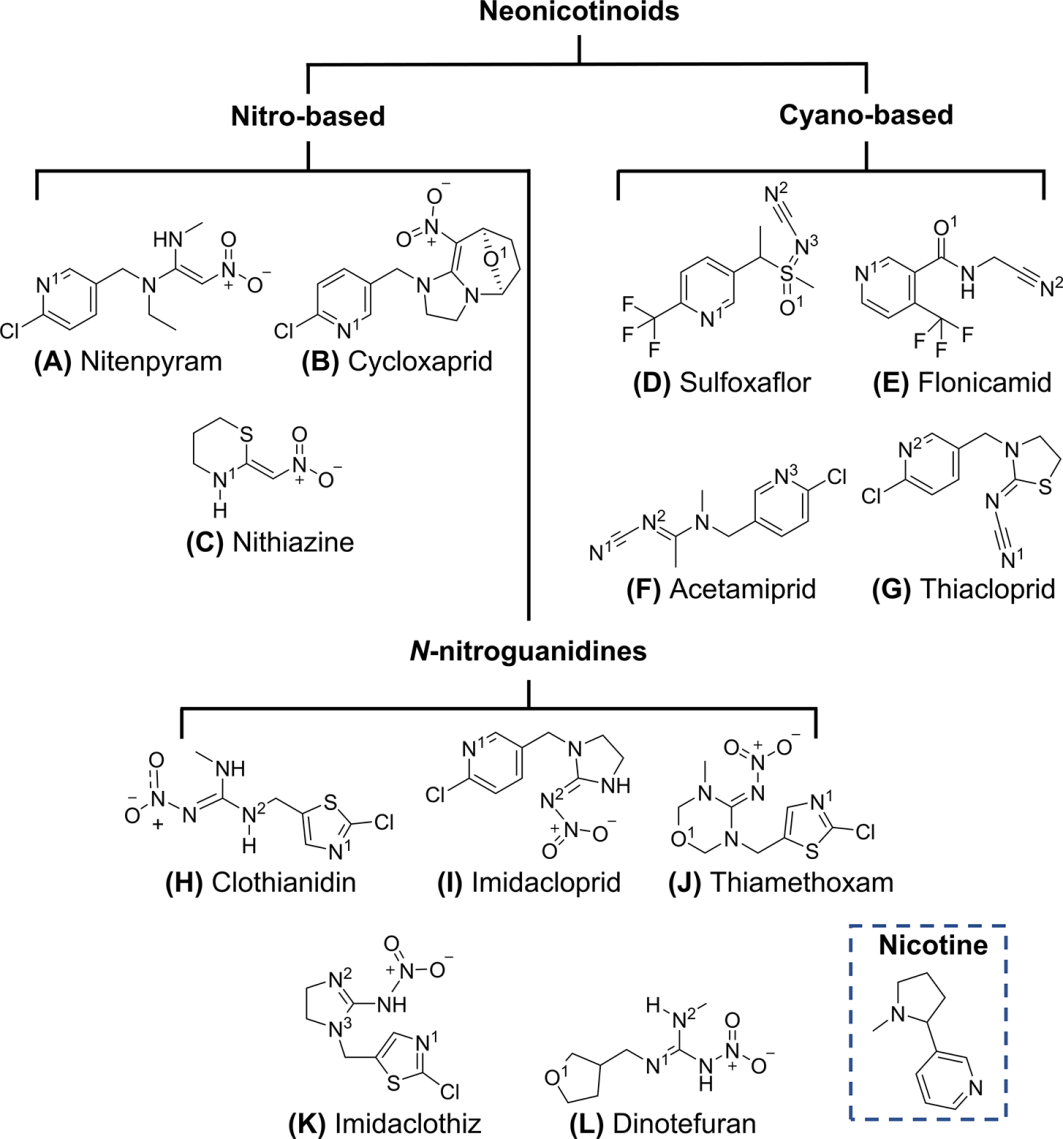
Chemical Structures of Neonicotinoid Pesticides and Nicotine

A class of systemic pesticides known as neonicotinoids (NNs) (Scheme 1) now dominates the global agricultural scene.^4,5,13^ NNs, literally interpreted as “new nicotine-like” pesticides, are now registered for combating chewing insects in over 120 countries and represent 25% of all insecticide sales worldwide (∼$3.7 billion in 2014).^4^ In the U.S., it is estimated that more than 3.5 million kg of NNs are applied to crops annually. Perceived as a safer alternative to organophosphate and carbamate-based pesticides while also offering broad spectrum toxicity, easy application, and high environmental persistence (fewer required treatments), NNs increasingly became the most commonly used commercial insecticide since the pyrethrum-based pesticides (1980s).^4,5,8,10,13^ Research suggests that NN usage has negative implications for both pollinating insects and human health, as the compounds are found in increasing concentrations in drinking water as well as children’s spinal fluid, blood, and urine.^14^ Additionally, it is now believed that targeted pests are developing resistance to certain NN pesticides, which causes an increase in the number and concentration of applications. This increase, in turn, exacerbates the risk to both pollinators and humans.^2,3,13,15^ In recent years, this risk has been recognized with mitigation attempts that include policy adjustments and legislation to slow or reverse the observed trends of declining pollinating insect populations.^4,16,17^ With the problems now being acknowledged, it is more critical than ever to develop practical tools to detect NN compounds on-site by nonexperts without requiring significant lab instrumentation. As such, research efforts have targeted NN detection, though the methodologies remain very instrumentation and/or personnel dependent,^15,18−21^ including fluorescence methods^22,23^ and more portable electrochemical techniques that often involve nanomaterials (NMs).^6,15,24,25^ With increasing legal measures to prohibit their use, the development of an easy-to-use, low-preparation NN detection system that is usable by nonexpert inspectors at manufacturing plants or pesticide storage centers on farms remains of high interest. As of today, commercial methodologies and materials of this nature are still rare, with only a few colorimetric test strips targeting organophosphate and carbonate pesticides.^15^

NN compounds (Scheme 1) can be subclassified into three structural categories: *N*-nitroguanidines (e.g., dinotefuran, Scheme 1L), nitromethylenes (e.g., nitenpyram, Scheme 1A), and *N*-cyanoamidines (e.g., acetamiprid, Scheme 1F) or NN-“like” compounds (e.g., cycloxaprid, Scheme 1B, and sulfoxaflor, Scheme 1D).^4^ While many NN structures share chloropyridine or furan moieties, it is the electron-rich functional groups (nitromethylene, nitroimine, and cyanoimine) that form the basis of their effectiveness as pesticides and that make them of interest in our study. Once ingested by a pest insect, NNs selectively and irreversibly bind to the nicotinic acetylcholine receptor (nAChR), which triggers nerve signaling. Acetylcholine esterase normally metabolizes acetylcholine but cannot break down the NN compounds, which cause sustained nerve stimulation at the receptor and eventually lead to paralysis and insect death. The nAChRs are present in significantly greater amounts in insects, making NNs ∼5-fold more selective in targeting insects compared to other pesticides.^4^

Chemical screening methods that can be used on-site for fast indication of the presence of a targeted compound are often associated with the forensic chemistry field and known as presumptive tests (e.g., Marquis test for opioid detection, luminol or leucomalachite green for blood detection, and Meisenheimer and Griess tests for gunshot residues).^26−33^ Many of these commercialized tests are colorimetric or spectroscopic based and used in the field to give a preliminary indication of the presence of specific chemicals without requiring significant time, trained personnel, or costly instrumentation. A colorimetric NP system was developed as an on-site testing method for determining the “age” and chemical composition of whiskey in wooden casks.^34^ Presumptive tests can be prone to occasional false positives and always require secondary confirmatory lab analysis (e.g., GC–MS). That said, they remain crucial on-site tools for identifying or narrowing unknown substances to a class of compounds, identifying specific chemical presence, or reporting solution conditions.^26,27^

**Figure 1:**
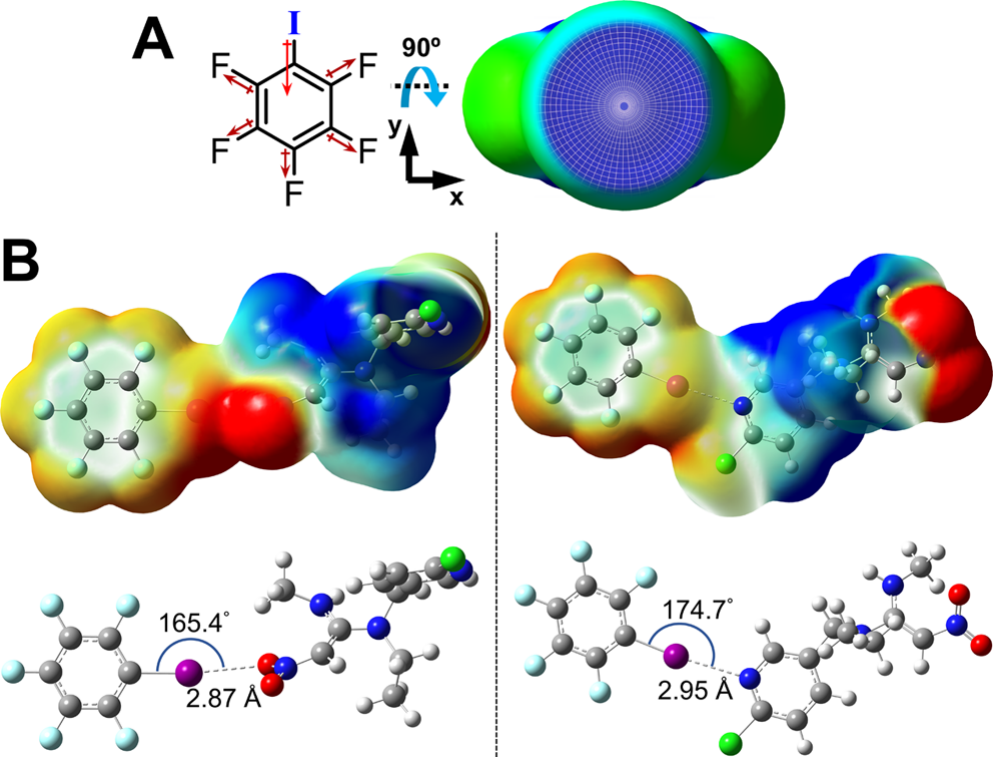
(A) IPFB represents a model XB donor molecule in which (left) electron-withdrawing fluorines on the aromatic ring create the overall dipole that results in an electron-deficient region known as the “σ-hole” at the iodine atom; the σ-hole is visualized from left to right after rotating IPFB’s structure 90° about the *x*-axis. (B) DFT-generated, space-filled electrostatic potential maps (top) and geometry-optimized structures (bottom) of XB adducts of IPFB (XB donor) with nitenpyram (XB acceptor) at the NO_2_ (left) and N^1^ (right) binding sites. Note: Geometry-optimized XB adducts of IPFB with other NNs are in Supporting Information (Figures S1–S20).

All chemical sensing methods require a fundamental interaction of significant strength to be established between the molecules of interest and the sensing platform. Recent work in our group has focused on exploring halogen bonding (XB) as a potential interaction to be exploited for such purpose. XB involves a positive region of electron deficiency (δ+), known as a sigma (σ) hole, found on a polarized halogen atom within one molecule (XB donor) interacting with an electron-rich region (δ−) on another molecule (XB acceptor).^35−37^ Molecular structure motifs, such as iodo-perfluoro-aromatic compounds, offer optimization of the σ-hole size (Figure 1A) and thus XB-donor strength.^38^ Here, the σ-hole of iodopentafluorobenzene (IPFB) at the iodine atom is created because of electron-withdrawing fluorine substitutions at multiple aromatic positions. Recently, we have reported the synthesis of thiolate ligands featuring an optimized XB-donor moiety (−C_6_F_4_I) featuring a σ-hole of significant strength. The thiol group opposite the XB-donor moiety on the ligand allows for the functionalization of the ligands onto the periphery of alkanethiolate-protected Au-NPs, known as monolayer protected clusters (MPCs),^39^ to create XB-donor functionalized MPCs (*f*-MPCs). By harnessing XB-donor capabilities in Au-NPs, the unique properties of Au-NPs, such as their surface plasmon resonance (SPR), can be utilized to directly observe XB interactions with XB-acceptor molecules.^40^

In this paper, XB interactions between *f*-MPCs (XB donors) and XB-acceptor sites on NN compounds are explored. While XB interactions between molecules have been extensively explored using computational tools, the translation of a theoretical understanding of XB interactions into a functional chemical system is a more rarely achieved phenomenon. Density functional theory (DFT) and diffusion-ordered NMR spectroscopy (DOSY NMR) are used to gain a greater fundamental understanding of interactions between electron-rich functional groups found on XB-acceptor NN compounds (e.g., nitromethylene, nitroimine, and cyanoimine—Scheme 1) and the XB-donor IPFB. XB interactions, along with hydrogen bonding (HB) interactions, have both already been identified as potentially playing a role in NN binding to insect nAChRs,^41^ which makes XB an interaction to explore in this capacity. Herein, we demonstrate that *f*-MPCs with XB-donor capability can detect the presence of NN compounds via XB-induced aggregation of the Au-NPs that is visually observable and further investigated using spectroscopy (NMR and UV–vis), dynamic light scattering, and electron microscopy—a proof-of-concept methodology with important environmental implications.

## 2 Experimental Details

### 2.1 Materials and Instrumentation

Chemical materials were purchased at the highest available purity (Millipore-Sigma, Oakwood Chemical, and TCI Chemicals), including NN compounds (Cayman Chemical, A2B Chem, and LGC Standards), and used without further purification or modification. NN compounds that were not tested were not commercially available at the time of the study. All aqueous solutions and experiments were prepared or conducted with 18.2 MΩ cm ultrapurified water. A high-performance supercomputer (SPYDUR) was used for DFT calculations. UV–vis spectroscopy data was collected on an Agilent 8453 Photodiode Array Spectrophotometer to characterize *unf*-MPC and *f*-MPC solutions and *f*-MPCs’ XB-induced aggregation events. Transmission electron microscopy (TEM; JEOL 1010 with Advanced Microscopy Techniques XR-100 CCD image collection) was operated at 80–100 kV for the assessment of the as-prepared MPC’s average diameter as well as to visualize XB-induced *f*-MPC aggregation events. NMR spectroscopy was carried out using a Bruker AVANCE III (400 MHz) spectrometer, with chemical shifts reported relative to tetramethylsilane and trichlorofluoromethane (CFCl_3_) for ^1^H and ^19^F NMR, respectively. NMR spectra were subsequently analyzed using Mestrelab’s MestreNova (v14.2) software. Dynamic light scattering (DLS) measurements were taken on a Malvern Instrument (model ZEN3600) at Virginia Commonwealth University (Carpenter Research Lab).

### 2.2 Computational Methodology

As in prior reports,^38,40^ DFT calculations of XB interactions carried out in the Gaussian16 software^42^ use the M06 functional^43^ with the cc-pVDZ (geometry optimization)^44^ and cc-pVTZ (single point) basis sets^45^ to estimate energy of interaction (Δ*E*_int_) or binding energy, XB bond lengths (X·B), and XB bond angles (R-X·B). In this study, relative comparisons rather than absolute energies were the goals of the computational analyses. More negative Δ*E*_int_ values, shorter X·B bond lengths, and more linear (180°) R-X·B bond angles indicated stronger XB interactions.^46^ The optimized geometries of all XB adducts were visualized using the GaussView program.^47^ Additional experimental details of computational measurements for this study are included in Supporting Information (Page S2).

### 2.3 NMR Measurements

As in prior studies,^40^ diffusion-ordered NMR spectroscopy (DOSY NMR) was used to measure the diffusion coefficients (*D*) of NN compounds (XB acceptors) and solvent molecules (control) in the presence and absence of either XB donor molecules like IPFB, control compound perfluorotoluene (PFT) (non-XB donor), or *unf*-MPC and *f*-MPC solutions. As in previous measurements of this nature for XB interactions,^38,40^ samples were prepared with equal concentrations of either IPFB (XB donor) or PFT (non-XB-donor control) and NN compounds (imidacloprid, nitenpyram, and thiacloprid) before transferring to 7 in.−5 mm OD heavy-wall NMR tubes (Norell) for DOSY NMR measurements. Similarly, *f*-MPC and *unf*-MPC solutions (whose concentration is equivalent to Abs_@518nm_ = 0.2 au in tetrahydrofuran (THF) or toluene) were well-mixed with either 11 mM nitenpyram, 50 mM imidacloprid, or 100 mM thiacloprid before transferring to heavy-wall NMR tubes (Norell) for DOSY NMR measurements. Additional DOSY NMR experimental parameters are provided in Supporting Information (Page S2).

### 2.4 Gold Nanoparticle Synthesis and Functionalization

Hexanethiolate-protected Au-NPs, also known as C6-MPCs, were synthesized via a modified Brust–Schiffrin reaction^48^ and characterized as previously described in significant detail.^40^ As in prior reports utilizing the same type of MPCs, characterization (UV–vis spectroscopy, NMR spectroscopy, and TEM imaging with histogram analysis) of C6-MPCs revealed an *average* diameter of 4.46 (±0.08) nm and a composition of Au_2951_(C6)_876_.^49^

C6-MPCs were converted to XB-donor functionalized MPCs (*f*-MPCs) via established place–exchange reactions^50^ with a previously in-house synthesized XB-donor ligand.^40^ The ligand features a 2,3,5,6-tetrafluoroidodobenzene moiety (−C_6_F_4_I) that has been shown in a prior study to engage in strong XB interactions.^38,40^ Place-exchanged (or functionalized) MPCs were purified via size-exclusion chromatography, as reported in the literature^51^ and previously replicated in our laboratory.^40^ In brief, the successful formation of *f*-MPCs bearing peripheral XB-donor ligands with −C_6_F_4_I moieties was confirmed using ^1^H and ^19^F NMR measurements after iodine degradation that liberates the *f*-MPCs’ peripheral ligands as disulfides, known as the iodine “death” reactions.^50^ From ^1^H NMR analysis, the *average* composition of the *f*-MPCs was estimated to be Au_2951_(C6)_438_(ligand-C_6_F_4_I)_438_ (equivalent to 50% degree of functionalization).^40^

### 2.5 Nanoparticle (MPC) Aggregation Experiments

The aggregation of *f*-MPCs via XB interactions or lack thereof for *unf*-MPCs was monitored and characterized via UV–vis spectroscopy, visual photography, DLS, and TEM imaging. For a typical sample, 600 μL of a *f*-MPC (or *unf*-MPC) solution (i.e., Abs_@518nm_ = 0.2 au in THF or toluene) was placed in a 0.75 mL-capacity, 10 × 2 mm pathlength, screw-capped, and quartz cuvette (Type 46, FireflySci). As demonstrated in the literature, gold NPs of this diameter correspond to an estimated extinction coefficient of 7.06 × 10^6^ M^–1^ cm^–1^, which Beer’s law translates to ∼28 or 70 nM for MPC solutions with Abs_@518nm_ = 0.2 or 0.5 au, respectively.^52^ UV–vis spectra of *f*-MPC (XB donor) or *unf*-MPC (non-XB material) solutions in THF or toluene (solvent selection promotes strong XB interactions^38^ and the solubility of NNs, *f*-MPCs, and *unf*-MPCs) were measured before and after the addition of XB-acceptor molecules (NN compounds or positive-control molecule DABCO) at different time intervals. Carefully measured masses of each tested NN compound were added to the *f*-MPC solution in the cuvette. Cuvettes were cleaned with aqua regia in between samples. Caution: Aqua regia, a 3:1 ratio of concentrated HCl/HNO_3_, is extremely dangerous, requires appropriate PPE, and should never be placed in a sealed container. Visual changes to the solution were recorded and compared to the as-prepared *f*-MPC (or *unf*-MPC) solutions without added XB acceptors. At various time intervals, aliquots (5 μL) of the *f*- or *unf*-MPC–NN mixtures were extracted, drop-cast on 200-mesh Formvar-coated (Electron Microscopy Sciences) or carbon-coated copper (SPI Supplies) TEM grids, allowed to dry inverted, and then imaged. Note: carbon-coated copper grids were more effective with aggregated samples as the bulk material often caused heating issues with the microscope electron beam. Each TEM grid was imaged ≥5 different areas and used for a composite characterization of the MPC materials at that stage of the analysis.

Interferent testing and limit of detection (LOD) determinations used a similar methodology as described above. UV–vis spectra of *f*-MPC solutions (XB donor) were measured before and after addition of 50 mM interferent compounds (including acetamiprid, clothianidin, parathion, chlorpyrifos, carbaryl, and dioctyl phthalate) in the presence or absence of 50 mM imidacloprid. For minimal analyte concentration detectable, UV–vis spectra of *f*-MPC solutions (Abs_@518nm_ = 0.2 au in THF or toluene, with an estimated concentration of ∼28 nM)^52^ were collected before and after the smallest amount of imidacloprid or nitenpyram that yielded a measurable and repeatable reduction in SPR intensity.

## 3 Results and Discussion

### 3.1 DFT Calculations

When exploring specific molecular interactions of an entire class of molecules, computational methods serve as an instructive tool to inform subsequent experimental design and execution in the lab. In the case of neonicotinoids (NNs) engaging in potential XB interactions with a model XB donor (e.g., iodopentafluorobenzene or IPFB), computational modeling with DFT allowed for the evaluation of several interaction parameters of each XB adduct: interaction energy (Δ*E*_int_), XB “bond” length (X·B) or distance, and XB “bond” angle (R-X·B, θ_XB_). Stronger XB interactions should correlate with more negative Δ*E*_int_ values, shorter X·B distances, and more linear R-X·B angles (θ_XB_ ∼ 180°). Figure 1B provides an example of the computationally modeled XB adduct of IPFB (XB donor) with the NN compound nitenpyram (XB acceptor) (Scheme 1A). It is immediately notable from DFT evaluation of the IPFB–nitenpyram adduct that this particular NN compound has two potential XB-acceptor sites for XB interactions with IPFB or other XB donors. DFT results of the IPFB–nitenpyram adduct show significant XB taking place at the nitro (NO_2_) group (Δ*E*_int_ = −11.38 kcal/mol) *and* at the nitrogen (N^1^) group (Δ*E*_int_ = −5.99 kcal/mol). To place these values in context, an XB adduct of IPFB (XB donor) with tributylphosphine oxide (Bu_3_PO, XB acceptor), established in prior work from our lab, exhibited a DFT-measured Δ*E*_int_ of −10.95 kcal/mol.^38,40^ For the context of intermolecular interaction strength, computational measurements (quantum mechanical calculations) show that HB within simple adducts of this nature exhibited Δ*E*_int_ values ranging between 7 and 11 kJ/mol (∼1.7–2.6 kcal/mol).^53^ While Δ*E*_int_ values energies provided guidance on XB interaction strength, we note that DFT-measured R-X·B angles at nitro groups (Figure 1B, left) were observed to deviate significantly from 180° because they are measured from one of the nitro oxygens rather than the midpoint of the bifurcated interaction with both oxygens. R-X·B angles, measured at the N^1^ acceptor site (Figure 1B, right), were consistently measured closer to 180° because the interaction involved only one lone pair of electrons on the nitrogen, with the smaller deviation attributable to other intermolecular interactions and steric effects present in molecules of this complexity. Optimized geometries of other NN compounds engaging in XB interactions with IPFB are provided in the Supporting Information (Figures S1–S20), and a summary of all interaction parameters for all IPFB−NN adducts is provided in Table 1. DFT analysis indicates that all the NNs explored in this work possess at least two XB-acceptor sites that could interact with the XB donor IPFB. The presence of two or more potentially strong XB-acceptor sites on the NN compounds is highly relevant to their proposed detection via functionalized NP (*f*-MPC)-based aggregation. For example, HB-capable ligands affixed to citrate-stabilized Au-NPs were shown to be able to use HB’s intermolecular interaction strength to detect the molecule melamine in solution via NP aggregation.^30^ Similarly, our prior work investigating XB interactions with Au-NPs showed that a model molecule with two XB-acceptor sites known as 1,4-diazabicyclo[2.2.2]octane (DABCO) was able to engage in strong enough XB interactions with *f*-MPCs featuring XB-donor −C_6_F_4_I moieties to induce an NP aggregation event in solution.^40^ As described in the next section, DOSY NMR measurements were successfully employed to confirm the XB interactions in the *f*-MPCs–DABCO mixture.^40^ For the current study, the collection of DFT results (Table 1) was then used to narrow the focus to the detection of specific NN compounds of high relevance and serve as proof-of-concept for establishing XB as a viable interaction to be exploited for their molecular detection.

**Table 1:** DFT Computational Interaction Energies (Δ*E*_int_), Bond Distances (XBD), and Bond Angles of XB Adducts of XB Donor IPFB and Various Neonicotinoid Compounds (XB Acceptors)

	neonicotinoid	XB acceptor sites	Δ*E*_int_ (kcal/mol)^a^	X·B distance (Å)	R-X·B angle (θ)
A	nitenpyram	NO_2_	–11.33	2.87	165.4
		N^1^	–5.97	2.95	174.7
B	cycloxaprid	N^1^	–6.36	2.94	174.3
		O^1^	–5.12	2.90	175.4
		NO_2_	–7.29	2.86	167.3
C	nithiazine	N^1^	–3.90	3.07	176.0
		NO_2_	–6.81	2.90	173.9
D	sulfoxaflor	N^1^	–5.06	3.01	173.7
		N^2^	–7.37	2.99	165.2
		N^3^	–5.11	3.06	164.9
		O^1^	–7.02	2.94	166.2
E	flonicamid	N^1^	–5.27	2.94	177.9
		N^2^	–3.93	3.04	179.2
		O^1^	–7.17	2.98	170.3
F	acetamiprid	N^1^	–6.25	2.93	178.5
		N^2^	–4.58	3.09	178.0
		N^3^	–5.68	2.96	173.9
G	thiacloprid	N^1^	–6.19	2.94	178.7
		N^2^	–6.71	3.08	171.0
H	clothianidin	NO_2_	–5.88	2.94	174.1
		N^1^	–4.95	2.98	177.4
		N^2^	–3.89	3.17	164.2
I	imidacloprid	NO_2_	–5.74	2.92	172.8
		N^1^	–6.07	2.95	173.5
		N^2^	–7.98	3.31	166.1
J	thiamethoxam	NO_2_	–6.34	2.90	171.3
		N^1^	–5.70	2.95	178.0
		O^1^	–3.53	2.95	174.6
K	imidaclothiz	N^1^	–5.51	2.96	178.4
		N^2^	–6.74	2.91	172.8
		N^3^	–3.69	3.19	173.2
		NO_2_	–3.64	3.01	174.9
L	dinotefuran	NO_2_	–5.05	3.02	164.3
		N^1^	–7.22	2.93	175.3
		N^2^	–5.36	3.00	171.0
		O^1^	–6.75	2.82	175.4

a Notes: Δ*E*_int_ = *E*(XB adduct) −[*E*(XB donor) + *E*(XB acceptor)] (gas-phase values). DFT functional and basis sets: M06-2X/cc-pVTZ//M06-2X/cc-pVDZ.

### 3.2 DOSY NMR Measurements of XB Interactions

Prior work in our group established DOSY NMR as a feasible method to detect XB interactions and quantitate their strength between equimolar mixtures of XB donor and XB acceptor molecules in solution.^40^ In principle, DOSY NMR-measured diffusion coefficients (D) of XB-acceptor molecules (e.g., NN compounds) should indicate significantly slower diffusion when they engage in XB interactions with a strong XB donor molecule like IPFB.^40^ In this study, a limited number of DOSY NMR measurements of this nature were conducted between IPFB and representative molecules from each NN subclass (Scheme 1), including nitenpyram (nitro-based NNs), imidacloprid (*N*-nitroguanidine-based NNs), and thiacloprid (cyano-based NNs). Results from these measurements and the corresponding control experiments are summarized in Table 2 and in the Supporting Information (Table S1) (i.e., the thiamethoxam system). The corresponding ^1^H NMR spectra of the tested NN compounds are provided in the Supporting Information (Figures S21 and S22). For the following discussion of DOSY NMR results, it is helpful to refer to the experiment number (#) in the first column of Table 2, where experiment #1–6, #7–12, and #13–18 examine the XB adducts of IPFB with nitenpyram, imidacloprid, and thiacloprid, respectively. For all the systems tested, log *D* values represent the relative diffusion (mobility) rate of the targeted molecules, with more negative values indicating slower diffusion/low mobility due to the presence of additional intermolecular interactions (i.e., XB interactions). For each NN system, in addition to interactions with IPFB, two major control measurements were conducted with PFT as a non-XB donor (i.e., a molecule of similar size/structure that does not engage in strong XB interactions) and solvent molecules (which were expected to remain relatively constant across the different experiments).

**Table 2:** DOSY NMR Measurements of Diffusion Coefficients (*D*) in Various Experimental XB and Control Systems^a^

#	XB acceptor	solvent	XB donor	target	log *D* (log(m^2^/s))
1	nitenpyram (11 mM)	toluene-*d*_8_	none	^1^H nitenpyram	–8.78 (±0.04)
2			IPFB (11 mM)		–8.86 (±0.04)
3			PFT (11 mM)		–8.80 (±0.03)
4			none	^1^H toluene-*d*_7_*h*_1_	–8.59 (±0.02)
5			IPFB (11 mM)		–8.62 (±0.03)
6			PFT (11 mM)		–8.60 (±0.03)
7	imidacloprid (50 mM)	tetrahydrofuran-*d*_8_	none	^1^H imidacloprid	–8.76 (±0.04)
8			IPFB (50 mM)		–8.84 (±0.04)
9			PFT (50 mM)		–8.77 (±0.04)
10			none	^1^H tetrahydrofuran-*d*_7_*h*_1_	–8.53 (±0.03)
11			IPFB (50 mM)		–8.52 (±0.02)
12			PFT (50 mM)		–8.52 (±0.04)
13	thiacloprid (100 mM)	tetrahydrofuran-*d*_8_	none	^1^H thiacloprid	–8.75 (±0.04)
14			IPFB (100 mM)		–8.77 (±0.05)
15			PFT (100 mM)		–8.76 (±0.04)
16			none	^1^H tetrahydrofuran-*d*_7_*h*_1_	–8.52 (±0.02)
17			IPFB (100 mM)		–8.52 (±0.05)
18			PFT (100 mM)		–8.53 (±0.04)

a Note: ^1^H toluene-*d*_7_*h*_1_ and ^1^H tetrahydrofuran-*d*_7_*h*_1_ refer to the ^1^H NMR residual solvent signals of toluene and THF molecules, respectively, which contain one less deuterium atom than toluene-*d*_8_ and tetrahydrofuran-*d*_8_, respectively.

Experiments #1–6 (Table 2) focus on nitenpyram in toluene in the presence and absence of IPFB or PFT. DOSY NMR measurements of ^1^H NMR signals of nitenpyram by itself and in the presence of PFT (experiments #1 vs #3) show similar values of −8.78 (±0.04) and −8.80 (±0.03). Notably, in the presence of IPFB (experiment #2), nitenpyram’s diffusion is notably slower, with a measured log *D* of −8.86 (±0.04). These results suggest that IPFB and nitenpyram engage in specific, strong XB interactions, which is further bolstered by the results of solvent control experiments #4–6 that show nearly identical log *D* values for protonated toluene in the same systems. The same trends are observed for the other NN systems as well. In experiments #7–9, imidacloprid (Scheme 1I) diffuses more slowly in the presence of IPFB (−8.84 ± 0.04) than in IPFB’s absence (−8.76 ± 0.04) or in PFT’s presence (non-XB donor) (−8.77 ± 0.04). The solvent controls again showed nearly identical log *D* values for protonated THF in the same systems (experiments #10–12). Similar results were obtained for the thiacloprid (Scheme 1G) system, though we note that the XB interactions between IPFB and thiacloprid were less substantial. Taken collectively, the DOSY experiments on these representative examples reinforce the results of the DFT calculations indicating that these molecules can engage in XB interactions. The question, however, remained as to whether such intermolecular interactions were strong enough to be harnessed for a specific purpose or application.

### 3.3 Detection of Neonicotinoid Compounds with the XB-Capable Functionalized Au-NPs—an Aggregation Model

Of all NN compounds, imidacloprid, or (2*E*)-1-((6-chloro-3-pyridinyl)methyl)-*N*-nitro-2-imidazolidinimine (Scheme 1E), developed by Bayer-CropScience in the mid-1990s, remains one of the most successful and widely utilized compounds of the group.^4,5^ On a global scale, the usage of imidacloprid, which features chloropyridine and nitroguanidine moieties, as a pesticide is second only to that of glyphosate.^54,55^ Additionally, imidacloprid is widely used in veterinary medicine as the main active ingredient in a number of prescribed topical treatments for flea, tick, and heartworm prevention/treatment (e.g., Advantage-Multi, Advantix, Seresto).^56^ As previously mentioned for NN compounds in general, the advantage of imidacloprid as a pesticide stems from its easy application (e.g., soil drenching, trunk injection, and spraying), environmental persistence (highly leachable with a half-life of 100–1250 days), and well-documented acute toxicity toward pest insects (EC_50_ of ∼0.86 μM for insect nAChRs vs two orders of magnitude higher for mammals).^4^ Unfortunately, imidacloprid has also been identified as one of the primary NN chemicals thought to negatively impact bee populations^1,57^ as well as having potential long-term effects on mammals.^4^ In 2013, in direct response to declining honey bee populations, the EU halted the use of imidacloprid on corn fields and identified the compound as a neurotoxin, while the U.S. EPA conducted a 2020 study on imidacloprid’s impact on human health.^4^ Concerns about imidacloprid were heightened as it started to be found in drinking water (unregulated) and fresh-water streams (Canada) and was prevalent in food crops.^4^ Simultaneously, studies involving mouse models showed imidacloprid exposure was related to reproduction development defects (i.e., teratogenicity effects), motor activity decline, and hepatotoxicity.^4,10^ Because of its prevalent usage globally, environmental persistence, and emergence in scientific literature regarding potential detrimental health effects, the development of a detection method for imidacloprid represents one of the most significant goals in this area of study, particularly if the method is fast and executable in the field by nonexperts.^6,15,25^ As such, imidacloprid is a major target molecule for our proposed XB-capable *f*-MPC detection scheme.

Structural DFT calculations of imidacloprid (Table 1I) show that, like many of the NN compounds examined, it exhibits three potential XB-acceptor binding sites. According to DFT calculations, sulfoxaflor, imidaclothiz, and dinotefuran (Table 1D,K,L, respectively) have four potential XB-acceptor sites, while nitenpyram and thiacloprid (Table 1A,G), have only two potential XB-acceptor sites. In the case of imidacloprid, DFT calculations identify three nearly equally strong XB-acceptor sites on the molecule: N^1^ in the chloropyridine group, N^2^ in the nitroguanidine group, and the terminal nitro (NO_2_) group. In theory, if each XB-acceptor site could strongly engage with a XB-donor moiety that could be easily monitored, the imidacloprid molecule could be detected via XB interactions (see below).

**Figure 2:**
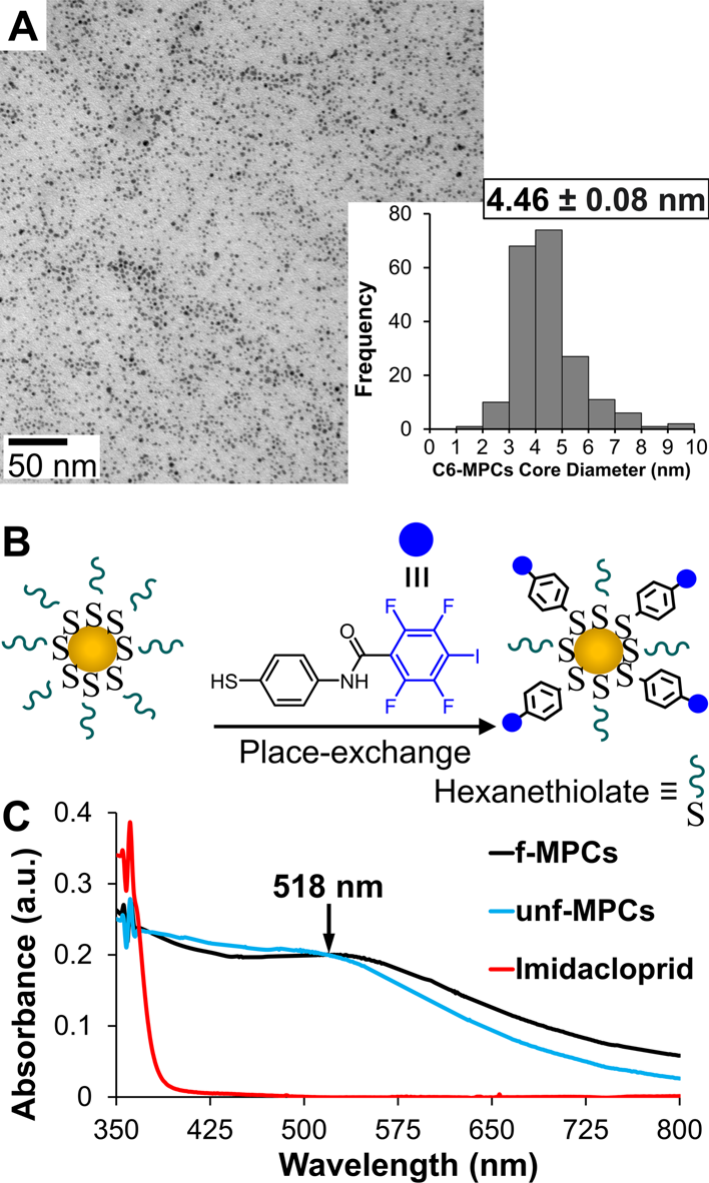
(A) TEM imaging and histogram analysis (inset) of the *average* core diameter of as-prepared C6-MPCs (or *unf*-MPCs). (B) Schematic representation of the functionalization of C6-MPCs with XB-donor ligands via place–exchange reactions that results in *f*-MPCs with XB-donor capabilities. (C) UV–vis spectra of *unf*-MPCs and *f*-MPCs in toluene with characteristic surface plasmon bands at ∼518 nm (Abs_@518_ = 0.20 au) as well as the spectrum of imidacloprid (50 mM) in THF.

Alkanethiolate-stabilized Au-NPs or MPCs are well-known for their stability in different solvents, ease of modification, and distinctive optical properties, including a strong SPR band, which can be readily observed using UV–vis spectroscopy.^39^ The SPR band observed with larger MPCs is a surface phenomenon captured when the oscillation of the collective electrons at the surface of the gold core comprising the Au-NPs matches the frequency of incident light and causes a broad absorption band in the visible region of the electromagnetic spectrum. Over the past two decades, research has established that the SPR band’s intensity and maximum absorbance (λ_max_) are specific to certain NP characteristics, including core size and composition, gold surface modifiers, and interparticle spacing, the last of which is most critical to the current study.^40^ Hexanethiolate-protected Au-NPs or C6-MPCs were prepared with an initial *average* composition of Au_2951_(C6)_876_ and the average diameter of 4.46 (±0.08) nm (Figure 2A) before surface-functionalization with an in-house prepared XB-donor ligand (Figure 2B) via well-known place exchange reactions^40^ to yield functionalized MPCs (*f*-MPCs) with an *average* composition of Au_2951_(C6)_438_(ligand–C_6_F_4_I)_438_. Notably, the *f*-MPCs feature XB-donor −C_6_F_4_I moieties extending from the MPC’s periphery into solution to facilitate XB interactions with XB-acceptor molecules. Figure 2C shows the characteristic SPR band at 518 nm of C6-MPCs (referred to as unfunctionalized MPCs (*unf*-MPCs) hereafter) red shifts slightly upon the functionalization of XB-donor ligands to form *f*-MPCs. Both the UV–vis spectra of *unf*-MPCs and *f*-MPCs are also compared to that of imidacloprid, which has nearly zero absorbance after 425 nm. UV–vis spectra of all the NNs examined in this study are provided in the Supporting Information (Figures S23–S27) for reference.

**Figure 3:**
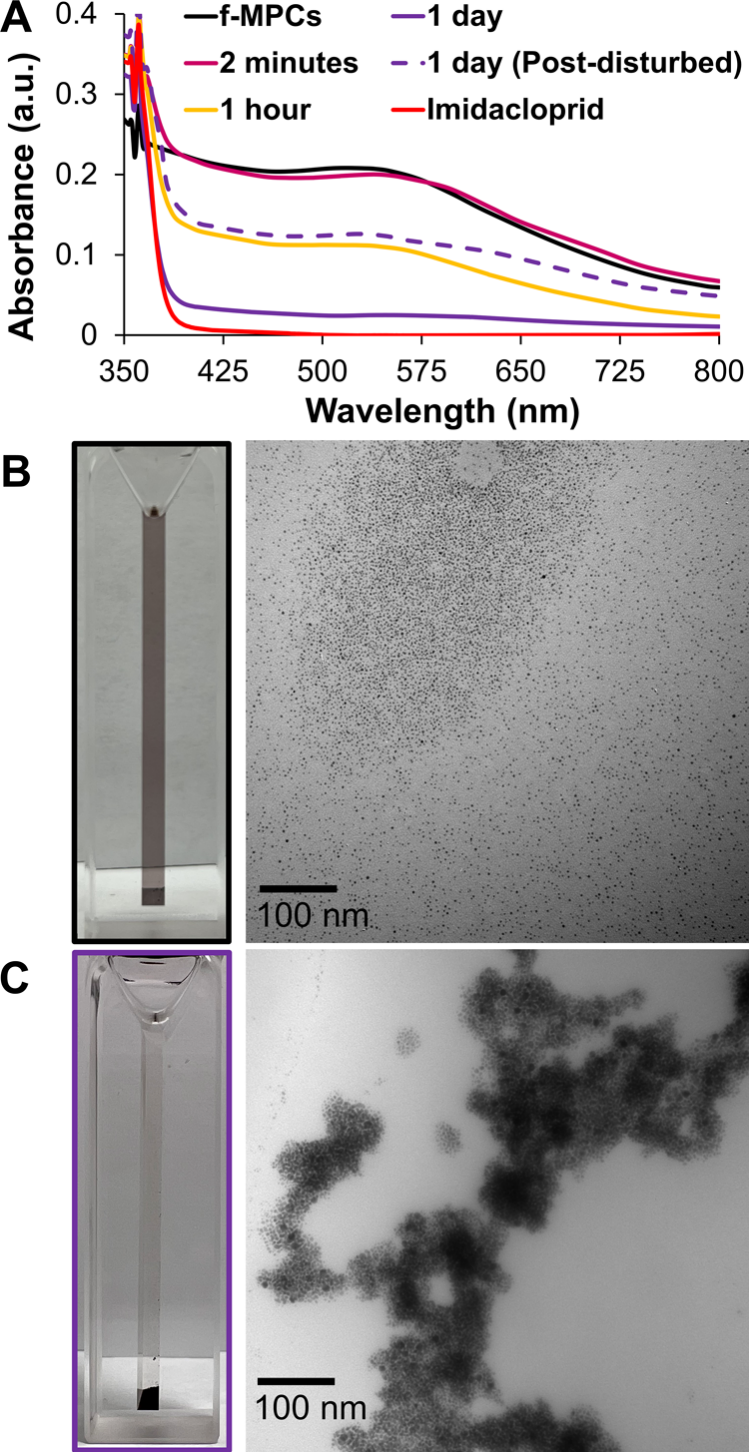
(A) UV–vis spectra of *f*-MPCs in THF upon exposure to imidacloprid (50 mM) over time. (B,C) TEM images (right) with visual pictures (left) of *f*-MPCs (B) before and (C) 1 day after the addition of imidacloprid (50 mM). The dashed UV–vis spectrum in (A) was taken briefly after the temporary resuspension of precipitated *f*-MPC aggregates at the bottom of the cuvette (after 1 day).

**Scheme 2:**
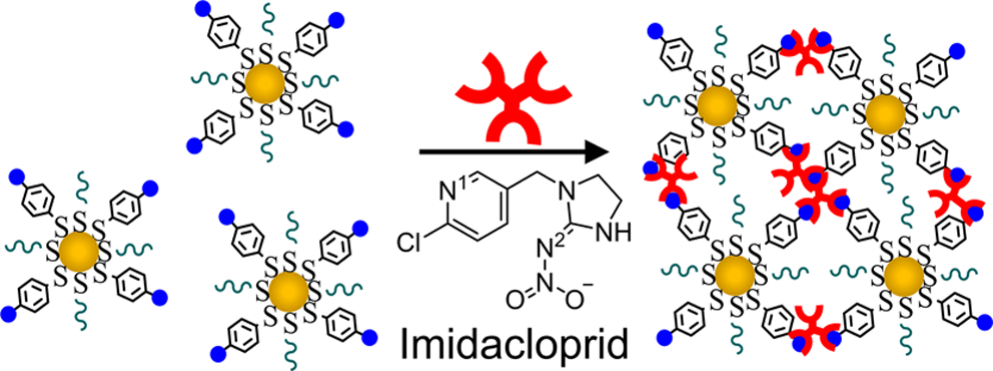
Illustration of Aggregation Events upon Exposure of *f*-MPCs (XB Donor) to Imidacloprid (XB Acceptor)

It was hypothesized that imidacloprid’s three potential XB-acceptor binding sites (identified by DFT calculations) would form XB interactions with multiple *f*-MPCs simultaneously via the XB-donor −C_6_F_4_I moieties. Engaging the multiple-site XB interactions should, in turn, decrease interparticle spacing between multiple *f*-MPCs and lead to NP agglomeration and eventual aggregation. In the proposed experiment (Scheme 2), the addition of imidacloprid to a *f*-MPC solution should result in the disappearance and/or significant red shift of the SPR band, which is consistent with other NP-based aggregation events in the literature.^30−32,40,58,59^Figure 3A shows the UV–vis spectra of the *f*-MPC solution in THF after the addition of imidacloprid. When imidacloprid was initially added to the *f*-MPC solution, the spectral signature of imidacloprid and the SPR band (Figure 3) of *f*-MPCs were both visibly evident. However, upon mixing, there was already an immediate red shift and a corresponding decrease in the absorbance of *f*-MPCs’ SPR band’s λ_max_. Both responses are consistent with the agglomeration of *f*-MPCs in solution due to diminished interparticle spacing that,^40^ in this case, was instigated by XB interactions between *f*-MPCs and imidacloprid. Over time, this XB-induced *f*-MPC agglomeration resulted in significant aggregation, with evident precipitation of aggregated *f*-MPCs on the bottom of the cuvette (Figure 3C, left). Figure 3B,C shows the expected TEM images of independent *f*-MPCs and aggregated *f*-MPCs before and 1 day after the addition of imidacloprid, respectively. TEM images of aggregated *f*-MPCs with imidacloprid after 2 min are provided in the Supporting Information (Figure S28). Over longer periods of time, aggregated *f*-MPCs eventually precipitated out of solution completely, and, if agitated, the UV–vis spectrum temporarily reflected the resuspension of *f*-MPC aggregates that precipitated once again in a matter of minutes (Figure 3A, dashed UV–vis spectrum).

As a natural extension of the study, it was of interest to explore if the *f*-MPCs would successfully detect other neonicotinoids that had either (a) similar structures to imidacloprid (e.g., clothianidin, thiamethoxam) or (b) different numbers of potential XB-acceptor sites (see below). Regarding the latter factor, nitenpyram (Scheme 1A) was targeted. Also developed in the mid-1990s (Sumitomo Chemical Takkeda Agro Co.), nitenpyram presents a fitting complementary NN compound for detection. While not nearly as widely used as imidacloprid, nitenpyram, featuring chloropyridine and nitromethylene groups, is another NN in the subclass of nitro-based NNs that are known to be more toxic than their cyano-family counterparts (Scheme 1). With a shorter half-life (weeks), moderate leaching, greater water solubility, and higher vapor pressure, nitenpyram is a fast-acting NN compound that has the potential to be found in water sources and more pedestrian (vs agricultural) applications.^2−4^ For example, veterinarians administer nitenpyram as an oral treatment (Capstar) for the immediate treatment of pets for flea and tick infestations prior to prescribing more long-term solutions with imidacloprid-based treatments. As such, nitenpyram is a popular target in the literature for sensing schemes.^18,60,61^ For this study, nitenpyram represents an interesting target because DFT calculations (Table 1) show only *two* viable XB-acceptor sites at the nitro (NO_2_) and N^1^ groups within the chloropyridine ring, the former being one of the most negative Δ*E*_int_ values recorded in Table 1. It was of interest to test nitenpyram to see if the number of XB-acceptor sites (>2) was required to achieve detection and/or if it provided some contextual selectivity to the method.

**Figure 4:**
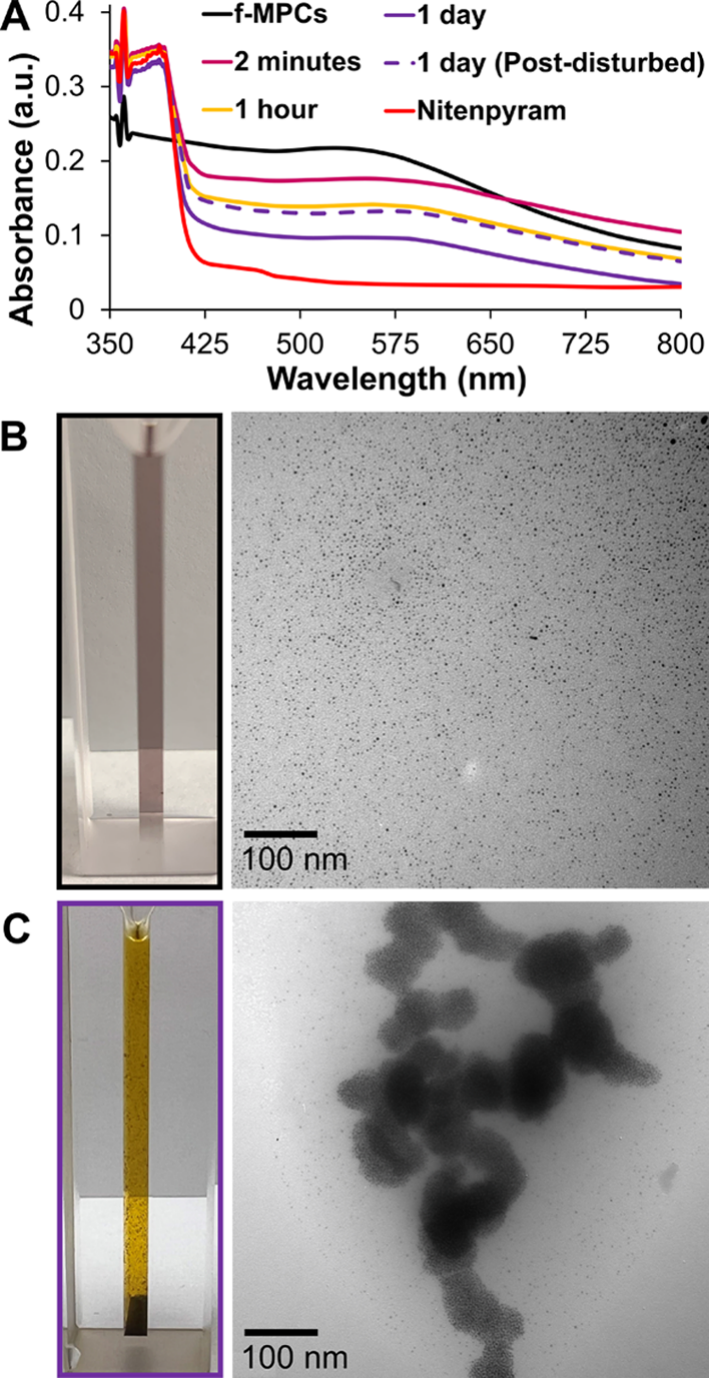
(A) UV–vis spectra of *f*-MPCs in toluene upon exposure to nitenpyram (11 mM) over time. (B,C) TEM images (right) with visual pictures (left) of *f*-MPCs (B) before and (C) 1 day after the addition of nitenpyram (11 mM). The dashed UV–vis spectrum in (A) was taken briefly after the temporary resuspension of precipitated *f*-MPC aggregates at the bottom of the cuvette (after 1 day).

Figure 4 captures the results of *f*-MPCs engaging in XB with nitenpyram and clearly shows that the system detects the nitenpyram in a similar fashion to imidacloprid. In brief, when nitenpyram was added to an *f*-MPC solution in toluene, the early UV–vis spectra (e.g., 2 min) (Figure 4A) reflected the spectroscopic signature of both *f*-MPCs and nitenpyram, including the expected and prominent SPR band at 518 nm of the MPC material (Figure 2). After mixing, over time, the same notable red shift in the SPR band and a significant decrease in absorbance due to XB-induced aggregation of the *f*-MPCs were observed. As with the imidacloprid system, aggregation could be visually confirmed as aggregates “crashed out” of solution (Figure 4C, left), a phenomenon that was observed in the corresponding TEM image (Figure 4C, right).

**Figure 5:**
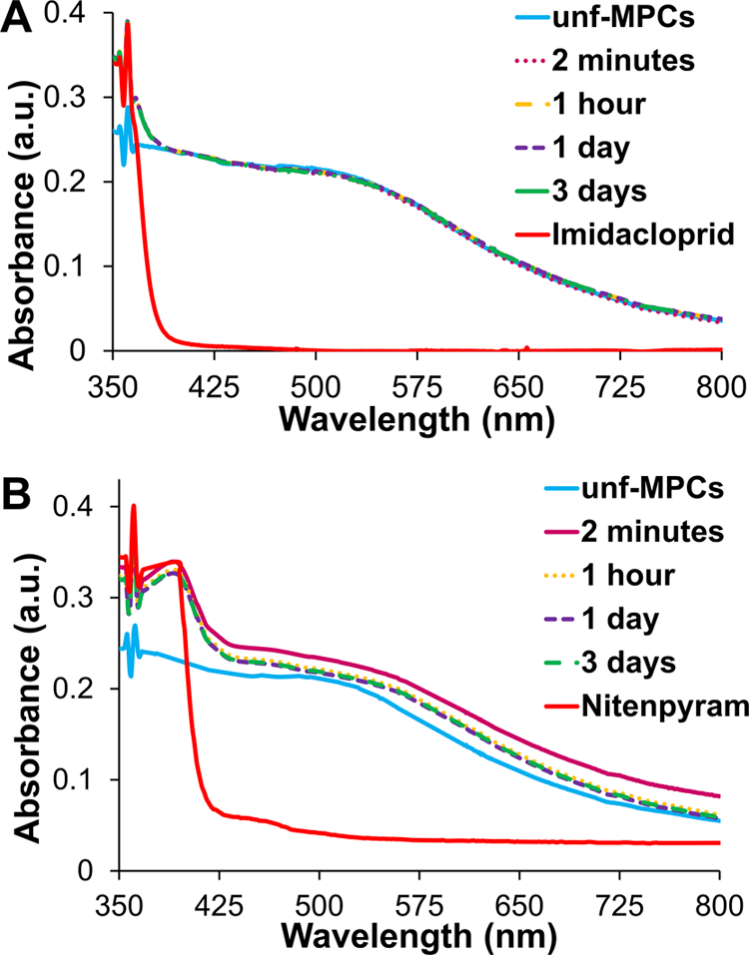
UV–vis spectra of control experiments exposing *unf*-MPCs to either (A) imidacloprid (50 mM) or (B) nitenpyram (11 mM) over time.

As in other NP aggregation-based molecular detection schemes, control experiments are key to demonstrating the observed phenomenon is due to XB interactions. As will be shown, these control experiments produced significantly different spectral trends. In the case of imidacloprid, it was mixed with *unf*-MPCs (not place-exchanged with the XB-donor ligands) that lacked the −C_6_F_4_I moieties to engage in XB interactions. As shown in Figure 5A, mixing *unf*-MPCs with imidacloprid did not result in the same UV–vis spectral behaviors expected for NP aggregation events, even after days of exposure. This result suggests aggregation of *f*-MPCs was indeed due to decreased interparticle spacing induced by significant XB interactions. Similar experiments mixing *unf*-MPCs with nitenpyram also showed no spectral shifts or diminished SPR signal (Figure 5B) on the scale observed with the *f*-MPCs–nitenpyram mixture, again supporting that the observed aggregation events were XB-induced (Figures 3 and 4). The *f*-MPCs–nitenpyram aggregation event was also successfully repeated using THF as the solvent (Supporting Information, Figure S31), an important result given that our prior work established that solvent can significantly affect the strength of XB interactions.^38^ Solvent effects are discussed more in the Conclusions section.

**Figure 6:**
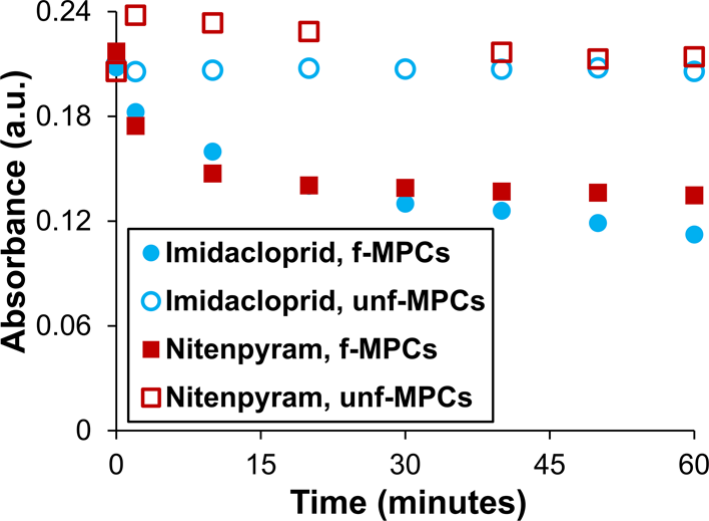
Spectral tracking of Abs_@518nm_ as a function of time for the mixtures of either *f*-MPCs or *unf*-MPCs exposed to either imidacloprid or nitenpyram (shorter time-scale analysis).

In terms of using *f*-MPCs for the quick detection of imidacloprid or nitenpyram, it is important to examine the UV–vis spectral response of the systems in the first few minutes versus hours after initial mixing. Figure 6 summarizes the UV–vis spectral responses in the early timeframe (1 h) for both *f*-MPCs and *unf*-MPCs exposed to imidacloprid and nitenpyram. As a quick detection system, the UV–vis spectra of *f*-MPCs in the presence of either NN compound exhibited a notable decrease of ∼35% in the first 20 min compared to only 1–2% decreases seen with corresponding *unf*-MPCs in the same timeframe. Longer timeframes (days) showed that the decrease in absorbance of the *f*-MPCs–imidacloprid mixture was more pronounced and ongoing than that of the *f*-MPCs–nitenpyram mixture after the first hour. In comparison, mixtures of *unf*-MPCs (incapable of XB interactions) with either nitenpyram or imidacloprid yielded stable UV–Vis spectra for days. The longer timeframe results are provided in the Supporting Information (Figure S29). It is notable that the behaviors of these mixtures (i.e., red-shifting SPR band and decreasing absorbance due to XB-induced aggregation of *f*-MPCs versus comparatively negligible UV–vis spectral changes seen with *unf*-MPCs) are entirely consistent with the previously reported XB-induced aggregation event of the same *f*-MPCs mixed with a model, two-binding-site XB-acceptor molecule, DABCO.^40^ This DABCO result was reproduced for the current study and shown in Supporting Information (Figure S30) as a positive control test for the functionality of the *f*-MPCs. Taken collectively, these results suggest that the systematic decrease in the SPR band of *f*-MPCs in the presence of these two NN compounds is directly attributable to XB-induced agglomeration and subsequent aggregation of these XB adducts, while no notable shifts or changes in the UV–vis spectra are observed with *unf*-MPCs in the presence of those same compounds.

^1^H DOSY NMR was used to measure the diffusion coefficients for nitenpyram, imidacloprid, and thiacloprid, each of which is representative of each major category of NN compound (Scheme 1), and solvent molecules (controls) in the presence of either *f*-MPCs or *unf*-MPCs. The results of these experiments are shown in Table 3; it is again useful to refer to the experiment number (#) in the first column when discussing the results. Essentially, the hypothesis of these experiments is that if there are significant XB interactions between *f*-MPCs and a NN compound, the latter will diffuse more slowly because of its strong, specific binding to the bulky *f*-MPCs. This effect should be absent with *unf*-MPCs, which cannot engage in XB interactions. This comparison of a NN with either *f*-MPCs or *unf*-MPCs is shown in Table 3 for nitenpyram (experiments #1–2), imidacloprid (experiments #5–6), and thiacloprid (experiments #9–10). Each NN compound exhibited a more negative log *D* value when mixed with *f*-MPCs than with *unf*-MPCs. While this supports the hypothesis, it is notable that nitenpyram and imidacloprid showed significantly more negative log *D* values with *f*-MPCs versus *unf*-MPCs than thiacloprid. These results agree with our aggregation experiments, in which *f*-MPCs aggregated and “crashed out” of solution in the presence of imidacloprid (Figure 3) or nitenpyram (Figure 4). Similar observations were absent in the mixtures of *f*-MPCs with thiacloprid or thiamethoxam (Supporting Information, Figures S32 and S33). The UV–vis spectra of the *f*-MPCs exposed to thiacloprid or thiamethoxam gradually decreased over three days but never resulted in visible *f*-MPC aggregation. This “selectivity” for imidacloprid and nitenpyram, believed to be related to NN solubility, is further discussed in the Conclusions section. Also notable from Table 3 are the log *D* values of solvent molecules (control experiments) in the MPCs–NN mixtures, which stay relatively constant across all respective system environments (experiments #3 vs #4 for toluene; experiments #7 vs #8 and experiments #11 vs #12 for THF).

**Table 3:** DOSY NMR Measurements of Diffusion Coefficients (*D*) of Nitenpyram, Imidacloprid, and Thiamethoxam in the Presence of u*nf*-MPCs and *f*-MPCs^a^

#	XB acceptor	solvent	XB donor	target	log *D* (log(m^2^/s))
1	nitenpyram (11 mM)	toluene-*d*_8_	*unf*-MPCs	^1^H nitenpyram	–8.65 (±0.06)
2			*f*-MPCs		–8.76 (±0.03)
3			*unf*-MPCs	^1^H toluene-*d*_7_*h*_1_	–8.55 (±0.07)
4			*f*-MPCs		–8.56 (±0.06)
5	imidacloprid (50 mM)	tetrahydrofuran-*d*_8_	*unf*-MPCs	^1^H imidacloprid	–8.63 (±0.04)
6			*f*-MPCs		–8.73 (±0.04)
7			*unf*-MPCs	^1^H tetrahydrofuran-*d*_7_*h*_1_	–8.48 (±0.03)
8			*f*-MPCs		–8.48 (±0.05)
9	thiacloprid (100 mM)	tetrahydrofuran-*d*_8_	*unf*-MPCs	^1^H thiacloprid	–8.61 (±0.06)
10			*f*-MPCs		–8.63 (±0.05)
11			*unf*-MPCs	^1^H tetrahydrofuran-*d*_7_*h*_1_	–8.49 (±0.06)
12			*f*-MPCs		–8.48 (±0.04)

a Notes: Concentrations of *unf*-MPCs and *f*-MPCs are equivalent to A_518_ = 0.50 au. DOSY NMR measurements of the sample of each NN with either *unf*-MPCs or *f*-MPCs were carried out 15 min after sample preparation. ^1^H toluene-*d*_7_*h*_1_ and ^1^H tetrahydrofuran-*d*_7_*h*_1_ refer to the ^1^H NMR residual solvent signals of toluene and THF molecules, respectively, that contain one less deuterium atom than toluene-*d*_8_ and tetrahydrofuran-*d*_8_, respectively.

**Figure 7:**
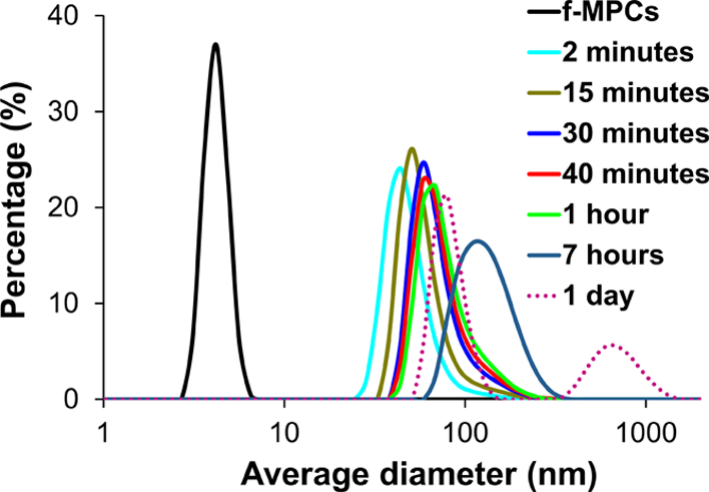
DLS results of *f*-MPCs in THF (Abs_@518_ = 0.20 au) before and after the addition of imidacloprid (12 mM) as a function of time. A control DLS experiment using *unf*-MPCs is included in Supporting Information (Figure S35) and shows no change in particle average diameter over time.

Given its widespread global use, imidacloprid detection using the *f*-MPCs was also observed using dynamic light scattering (DLS). As shown in Figure 7, a solution of *f*-MPCs prior to exposure to imidacloprid had an expected average diameter of ∼4.5 nm that is consistent with TEM histogram measurements. Upon exposure of *f*-MPCs to imidacloprid, there was nearly immediate evidence of increased NP average diameter due to the *f*-MPCs agglomerating in solution after only 2 min. Persistent increases in particle size over time were observed for the *f*-MPCs in the presence of imidacloprid, particularly over the first hour. After 24 h, nearly complete aggregation or visible precipitation of *f*-MPCs was again observed. DLS measurements of the 1 day sample after being physically perturbed via inversions show a significantly broadened peak, representing even larger particle average diameter and large aggregates temporarily suspended in solution. An analogous experiment using a solution of *unf*-MPCs showed no change in NP average diameter after exposure to imidacloprid over the same time frame (Supporting Information, Figure S35).

### 3.4 Interferent and LOD Considerations

With any molecular detection scheme, even one that targets on-site, fast identification of an unknown powder or heavy residue that can be collected, it is important to establish effective selectivity in the presence of interferent species and sufficient sensitivity in terms of detection limits toward a targeted analyte. In both cases, the much more desirable NN target molecule remains the increasingly used and environmentally dangerous imidacloprid. As such, the *f*-MPC scheme was executed for imidacloprid in the presence of four different interferent groups: (1) other non-aggregating NN pesticides (acetamiprid and clothianidin, Scheme 1H,F; see Section 3.5 below); (2) commonly employed organophosphate pesticides (parathion and chlorpyrifos); (3) a commonly used carbamate pesticide (carbaryl);^62^ and (4) a plasticizer commonly found in the environment (dioctyl phthalate). As conducted in other studies of this nature,^23^ simple mixtures of these compounds were added to *f*-MPC solutions in the presence and absence of imidacloprid. UV–vis spectra collected for these experiments show that, in all cases, *f*-MPCs aggregated in the presence of imidacloprid as expected and regardless of the presence of any of the interferents, while analogous control solutions containing only the interferent species with no imidacloprid resulted in no evidence of aggregation on the time-scale of the experiment (24 h). These results are shown in Supporting Information (Figures S36–S38) and establish that these *f*-MPCs are highly selective for imidacloprid even when other NN compounds, environmental contaminants, or other organophosphate or carbamate pesticides are present in significant amounts (50 mM).

**Figure 8:**
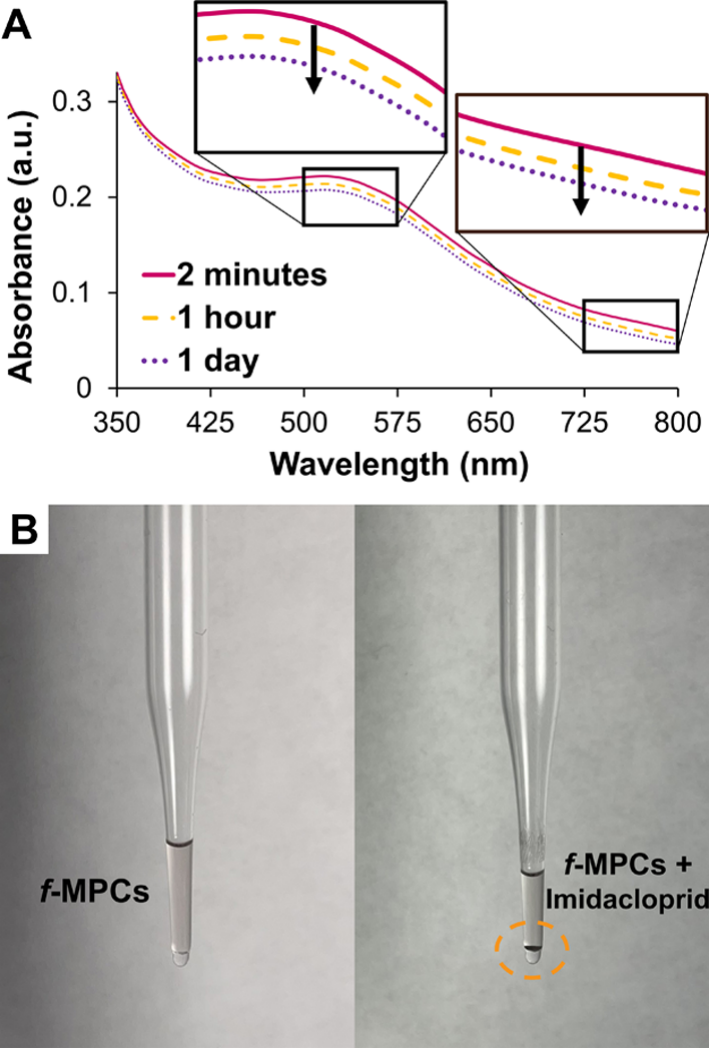
(A) UV–vis spectra of *f*-MPCs in THF exposed to 1 μM imidacloprid over time. (B) Visual images of 10 μL of *f*-MPC solution in THF in a sealed Pasteur pipette before (left) and 1 h after (right) the addition of 25 μg of imidacloprid. Note: A similar analysis of nitenpyram resulted in a minimal mass of 11 μg and a LOD of 1 μM (Supporting Information, Figure S40).

In terms of sensitivity, NP aggregation schemes of this nature have two major considerations when it comes to LOD evaluation. Like other colorimetric or visual NP aggregation schemes,^58^ it is not unusual to have *both* a visible (no calibration curve) and an instrumental-based LOD, the latter requiring a hand-held device to be developed. Indeed, given that aggregation was observed instrumentally using spectroscopy, DLS, and TEM, it seems that the instrumental LOD detection will be linked to the ability to detect the *f*-MPCs themselves. In this study, spectroscopy was mainly used to correlate response to the visual aggregation of the *f*-MPCs, which means that the LOD is linked to the *ratio* of soluble NN molecules to *f*-MPCs as well as the available instrumentation capability to observe aggregation. For a visible indication of imidacloprid, we systematically lowered the mass of the NN in the sample holder to determine the minimum concentration of imidacloprid that caused obvious aggregation. Spectroscopically, we based our estimated LOD on the minimum concentration of imidacloprid that caused a measurable and repeatable spectral shift and/or decrease. In this manner, as shown in Figure 8, aggregation was visible with 1 μM imidacloprid (or 25 μg), while spectral shifts suggested a 3 μM LOD for imidacloprid (Supporting Information, Figure S39). In either case, it is important to note that visual observation of aggregation will depend on the size/shape of the sample holder to see NP precipitation, while the development of a dedicated, hand-held spectroscopic instrument with a smaller volume chamber for the sample would likely achieve a lower LOD.

### 3.5 Less Prominent Neonicotinoid Compound Testing

Even though it is not used as extensively as imidacloprid in NN applications, XB-induced aggregation of *f*-MPCs was also investigated for other *N*-nitroguanidine-based NNs with similar structures to imidacloprid, including clothianidin, thiamethoxam, and dinotefuran (Scheme 1H,J,L, respectively). Interestingly, for thiamethoxam, red-shifts of *f*-MPCs’ SPR band and decreasing absorbance in UV–vis spectra were again observed, though eventual aggregation was not visible on the same timescale (Supporting Information, Figure S33). It is speculated that this is related to the solubility limits of thiamethoxam in that there was not enough of the NN compound solubilized to interact with a significant number of *f*-MPCs, a concept discussed further in the Conclusions section. The UV–vis spectra of *f*-MPCs in the presence of clothianidin and dinotefuran did not diminish over time (Supporting Information, Figures S33 and S34), an example of systems that likely did not engage in significant XB interactions. Another *N*-nitroguanidine-classified NN compound called imidaclothiz (Scheme 1K) was not tested experimentally due to a lack of commercial availability.

Cyano-based NN compounds sulfoxaflor, acetamiprid, and thiacloprid (Scheme 1D,F,G, respectively) were tested with *f*-MPCs, while flonicamid (Scheme 1E) was not tested for XB-induced aggregation. Spectroscopic tracking of the UV–vis spectra of *f*-MPCs exposed to sulfoxaflor, acetamiprid, and thiacloprid showed very little spectral shift or decrease over time, suggesting the absence of XB-induced aggregation (Supporting Information, Figures S31 and S32). These systems were again severely limited by the solubility of the compounds in the solvent (THF). However, it is also reasonable to infer from the collective experimental and computational results that the cyano groups in this subclass of NN compounds may simply represent weaker XB acceptors (vs nitro groups) that are unable to engage in strong enough XB interactions for a measurable response.^63^ That said, the stability of *f*-MPCs observed with clothianidin and dinotefuran (nitro-containing NN compounds) suggests that solubility may play the most critical role (see the Conclusions section). Cycloxaprid and nithiazine, the remaining nitro-based NN compounds (Scheme 1B,C, respectively), were not tested in this study due to their insolubility in toluene and THF (tested solvents) and commercial unavailability, respectively. As such, based on the significant number of NNs tested, the specific *f*-MPC aggregation-based detection scheme in this study appears to be self-selective for signaling the presence of two critical NN compounds: imidacloprid, one of the most widely used NNs globally, and nitenpyram.

### 3.6 Imidacloprid Sensors

Even though the focus of our study was the functionalized NPs and their XB-induced aggregation, we recognize that these materials could serve as a functional component of a sensing system. As with the development of any sensing scheme, it is important to contextualize the potential analytical performance in the broader field, where several sensors targeting imidacloprid have been developed.^64−69^ A study in 2022 employed the fluorescence signal of a Zr metal–organic structure and its aggregation to detect imidacloprid and thiamethoxam with excellent LODs, including in real samples of fruit juice analyzed in a laboratory environment. Additionally, the report tested the selectivity of the sensor against only three other NN compounds.^66^ An electrochemical sensor, reported in 2022 by Harraz et al., featuring an electrode modified with a composite film of Ag-NPs in a carbon/hematite ore, performed in a similar capacity to this study in terms of sensitivity, yielding detection in the μM range with a LOD of ∼1 μM. As with most modified electrodes, it is susceptible to fouling, particularly in environmental testing, and, in this study, was tested against an interferent array not including other pesticides.^67^ A very encouraging trend in the literature on imidacloprid sensors is the number of colorimetric/spectroscopic studies utilizing the same techniques used in our study (i.e., UV–vis spectroscopy and TEM imaging),^64,65,68−70^ including some reports employing different Au-NPs and observing similar magnitude spectral shifts.^64,68,69^ Moghaddam and coworkers demonstrated colorimetric sensing of imidacloprid using graphene–Au quantum dots (QDs) that visibly changed the color, though sometimes visibly indistinguishable between certain concentrations, or exhibited spectroscopy attenuation of the SPR in the presence of only ppm pesticide—the latter technique was required for imidacloprid quantitative analysis. The QDs, which interacted with imidacloprid’s imidazole group, are non-trivial to synthesize and were demonstrated to detect the pesticide on vegetables requiring significant preparation after simulated treatments that may or may not be the expectation in the environment. Targeting the imidazole group has the disadvantage that imidacloprid is not the only NN compound with that functionality.^70^ A study by Feng et al. this year (2023) used colorimetry/spectroscopy with Prussian blue polymer NPs for imidacloprid detection. However, their selectivity testing did not include other NN compounds and focused on other types of pesticides, some of which yielded the same colorimetric response as imidacloprid (parathion and fenthion). Interestingly, these authors observed that a contributing factor to their observed selectivity was the polarity of their solvent^65^—a similar conclusion to our study as discussed below. Other researchers who employed Au-NPs to detect imidacloprid used visible spectroscopy to monitor water-soluble Au-NPs (i.e., citrate-based Au-NPs), reporting small SPR spectral shifts, like our study, with exposure to increasing imidacloprid concentrations as well as similar LODs (0.5–1.0 μM).^68,69^ These types of aqueous NPs, some requiring intensive synthesis for this application, are notorious for poor long-term stability, with one report indicating that the NPs only lasted 15 days.^68^ Another recent study by Zhao and coworkers employed thiol-protected Au-NPs, which are more stable, but their methodology required the use of an automated shaker and colorimetric imager.^64^ In all the Au-NP studies mentioned, however, imidacloprid selectivity was established in the presence of other non-neonicotinoid pesticides and only a limited number of competing neonicotinoid compounds. Assessed collectively, we can surmise a number of advantages of our proposed NN detection scheme using our *f*-MPCs compared to other systems in the literature, including (1) high selectivity for imidacloprid against an array of other NN pesticides (the only exception being nitenpyram); (2) the use of highly stable Au-NPs where the *f*-MPCs remained selective for imidacloprid for a year or more; and (3) the aggregation, which is a visible, non-instrumental indication of the presence of the NN compound even if interferents are present in significant amounts. In addition to these advantages, the *f*-MPC scheme presented appears to have response times and LODs of similar magnitude to the aforementioned sensor literature, suggesting the functionality of these materials is promising if developed into an instrumental, hand-held sensor.

## 4 Conclusions

The goals of this research project were two-fold: (1) achieving a greater fundamental understanding of XB interactions between strong XB-donor moieties and different types of NN compounds; and (2) the synthesis of XB-donor functionalized MPCs (*f*-MPCs) that serve as the functional component of uniquely functionalized NMs capable of aggregation-based detection of one of the most widely used NN pesticides globally, imidacloprid—a platform that can serve as the basis for developing detection tools that do not require instrumentation or trained personnel. The observed aggregation events of *f*-MPCs featuring strong XB-donor ligands, which are selective for only imidacloprid and nitenpyram, establish the viability of using these NMs for such an application. While the *f*-MPC system in this study is self-selective for imidacloprid and nitenpyram, the results suggest that these systems may have more applications and offer parameters that can be tuned for selectivity toward other compounds of interest. Like other NP aggregation schemes,^34^ we strongly suspect that our system can be adjusted to be applied for other NN compounds, albeit with important limitations. A critical component of this methodology, established here and in other studies, is the role of solvent in XB-based systems, where certain solvents can “shield”/weaken the XB interactions.^38,40,71^ Absent that shielding effect of the solvent, our demonstrated experimental system is effective because *both* the specific-diameter *f*-MPCs *and* the targeted NNs are soluble in a solvent that promotes strong XB interactions. For example, while imidacloprid, nitenpyram, and *f*-MPCs were soluble in THF, we suspect that sulfoxaflor, acetamiprid, thiacloprid, clothianidin, thiamethoxam, and dinotefuran are not soluble in THF to the same degree and thus self-limiting in detection by this method. Given our understanding and ability to manipulate the solubility of *f*-MPCs (e.g., altering gold core sizes, peripheral ligand properties/functional groups, and degrees of XB-donor ligand functionalization), it is conceivable that specific *f*-MPC systems could be designed for other NN compounds—studies that are currently underway in our laboratory. The current work represents a fundamental proof-of-concept application of XB-capable *f*-MPCs for fast, on-site identification of imidacloprid and nitenpyram. One can envision dipping a cotton swab sampling of an unknown powder found at a pesticide manufacturer or storage area into a *f*-MPC solution, which then exhibits XB-induced NP aggregation and thus yields a preliminary indication of the presence of imidacloprid, an increasingly regulated or prohibited pesticide in many parts of the world.

## Supporting Information Available

Experimental details of DFT calculations and DOSY NMR measurements; DFT geometry-optimized XB adducts of IPFB with all neonicotinoid compounds; DOSY NMR measurements of thiamethoxam in the presence of IPFB, PFT, *f*-MPCs, and *unf*-MPCs; ^1^H NMR spectra of DOSY-tested neonicotinoid compounds; UV–vis spectra of neonicotinoid compounds; UV–vis spectra and/or TEM images of aggregation events of *f*-MPCs with nitenpyram (in THF), imidacloprid (after 2 min), and DABCO (positive control); UV–vis spectral tracking over long periods for *f*-MPCs and *unf*-MPCs (control) exposed to imidacloprid, nitenpyram, and DABCO; UV–vis spectra of *f*-MPCs in the presence of less prominent NN compounds; DLS results of *unf*-MPCs with imidacloprid; interferent testing with other NN compounds; imidacloprid calibration curve; and UV–vis spectra and visual imaging of *f*-MPCs with nitenpyram (PDF)
