# Supplementary material for: On-Site Detection of Neonicotinoid Pesticides Using Functionalized Gold Nanoparticles and Halogen Bonding
Source: ACS Appl Nano Mater. 2023 Apr 24;6(10):8367–81. doi: 10.1021/acsanm.3c00618 (PMC10227770; doi:10.1021/acsanm.3c00618)
Supplement: Supplementary file 1 — an3c00618_si_001.pdf [file an3c00618_si_001.pdf]

Supporting Information

On-site Detection of Neonicotinoid Pesticides Using Functionalized Gold Nanoparticles and Halogen Bonding

Molly M. Sherard,= Quang Minh Dang, = Sophia C. Reiff, Jeffrey H. Simpson, and Michael C. Leopold*
Department of Chemistry, Gottwald Science Center, University of Richmond, Richmond, VA 23173
*Corresponding author: mleopold@richmond.edu

Table of Contents:

- Experimental details of **density functional theory (DFT)** calculations and **diffusion ordered spectroscopy (DOSY) NMR** measurements – p. S2
- **Figures S1 to S20:** Geometry-optimized XB adducts of **XB donor iodopentafluorobenzene (IPFB)** with various XB-acceptor binding sites on **neonicotinoid** compounds: **(B) cycloxaprid** (pp. S3–S4), **(C) nithiazine** (p. S5), **(D) sulfoxaflor** (pp. S6–S7), **(E) flonicamid** (pp. S8–S9), **(F) acetamiprid** (pp. S10–S11), **(G) thiacloprid** (p. S12), **(H) clothianidin** (pp. S13–S14), **(I) imidacloprid** (pp. S15–S16), **(J) thiamethoxam** (pp. S17–S18), **(K) imidaclothiz** (pp. S19–S20), and **(L) dinotefuran** (pp. S21–S22).
- **Tables S1 to S2:** DOSY NMR measurements of **(J) thiamethoxam** and **protonated tetrahydrofuran** (control) in the presence of **IPFB** (XB donor), **perfluorotoluene** (non-XB donor), ***f*-MPCs**, and ***unf*-MPCs**. – p. S23
- **Figures S21 to S22:** ^{1}H NMR spectra for tested **neonicotinoid** compounds in tetrahydrofuran-d_8 (unless otherwise stated) including: **(A) nitenpyram** in toluene-d_8 (p. S24), **(G) thiacloprid** (p. S24), **(I) imidacloprid** (p. S25), and **(J) thiamethoxam** (p. S25).
- **Figures S23 to S27:** UV-Vis spectra for tested **neonicotinoid** compounds in tetrahydrofuran (unless otherwise stated) including: **(A) nitenpyram** in toluene and in tetrahydrofuran (p. S26), **(D) sulfoxaflor** (p. S27), **(F) acetamiprid** (p. S27), **(G) thiacloprid** (p. S28), **(H) clothianidin** (p. S28), **(I) imidacloprid** (p. S29), **(J) thiamethoxam** (p. S29), and **(L) dinotefuran** (p. S30).
- **Figure S28:** TEM analysis of aggregation events of **(I) imidacloprid** with ***f*-MPCs** in tetrahydrofuran after 2 minutes. – p. S31
- **Figure S29:** Spectral tracking of $Abs_{@518nm}$ as a function of time of the mixtures of either ***f*-MPCs or *unf*-MPCs** exposed to either **(I) imidacloprid**, **(A) nitenpyram**, or **DABCO** (longer time-scale analysis). – p. S32
- **Figures S30:** UV-Vis spectra and TEM analysis of aggregation events of ***f*-MPCs** with **DABCO** in toluene. – p. S33
- **Figures S31 to S34:** UV-Vis spectra of ***f*-MPCs** exposed to other **neonicotinoid** compounds in tetrahydroguran over time including: **(A) nitenpyram** (p. S34), **(D) sulfoxaflor** (p. S34), **(F) acetamiprid** (p. S35), **(G) thiacloprid** (p. S35), **(H) clothianidin** (p. S36), **(J) thiamethoxam** (p. S36), and **(L) dinotefuran** (p. S37).
- **Figure S35:** DLS results of *unf*-MPCs before and after exposure to imidacloprid (12 mM) – p. S38
- **Figures S36 to S38:** Results of interference tests with **other neonicotinoid** compounds (**acetamiprid and clothianidin**, p. S39), common **organophosphate** pesticides (**parathion** and **chlorpyrifos**, p. S40), a common **carbamate** pesticide (**carbaryl**, p. S40), and a **plasticizer** (**dioctyl phthalate**, p. S41).
- **Figure S39:** Imidacloprid calibration curve with *f*-MPCs in THF with limit of detection reported. – p. S42
- **Figure S40:** UV-Vis spectra of *f*-MPCs exposed to 1µM nitenpyram and visual images of *f*-MPC aggregation with 11µg nitenpyram. – p. S43
- **References** – p. S44

Density Functional Theory (DFT) Analysis

Gas-phase geometry optimizations and single-point calculations of the XB donors, XB acceptors, and XB adducts were performed using the Gaussian16 software[1] with the M06 functional[2] and the cc-pVDZ[3] (geometry optimization) and cc-pVTZ[4] (single point) basis sets. For the larger atoms (iodine, bromine, and selenide), the small (28–e^-) Dirac-Fock (MDF) effective-core pseudopotentials and the corresponding basis sets were used.[5, 6] Frequency analyses were conducted to confirm that the geometry-optimized structures corresponded to true minima (i.e., no imaginary frequencies) on the respective potential energy surfaces. The energy of interaction (ΔE_{int}) or binding energy between XB donors and acceptors was used to estimate the thermodynamic favorability and stability of the XB adducts. Specifically, more negative ΔE_{int} values suggest more thermodynamically favorable adducts. Zero-point energy and basis set superposition corrections were not included in the ΔE_{int} calculations based on the assumption that these harmonic corrections would be very similar for each system as the adducts are structurally similar. The XB bond lengths (X••B) and XB bond angles (R–X••B) of all XB adducts were also obtained as additional indicators of the strength of XB interactions. Specifically, strong XB interactions are typically characterized by bond lengths shorter than the van der Waals distances of the interacting atoms and nearly linear (180°) R–X–B bond angles.[8-12]

Experimental Details of Diffusion-Ordered NMR Spectroscopy (DOSY) Measurements

DOSY spectra with convection-compensation were collected using thick-walled 5mm NMR tubes. The DOSYcc parameter set in Bruker Topspin v3.2 was used with the following parameter configuration. The relaxation delay was 3 seconds with 90-degree 1H RF pulses of 12 microseconds and 180-degree 1H RF pulses of 24 microseconds. 16 scans were collected over 16 gradient increments from 2% to 95% of the gradient strength, using a linear gradient modulation with a d20 of 60 ms. The data set was processed with an f2 dimension of 16k and an f1 dimension of 32. The dosy2d command in Topspin was executed to process the data set. DOSY peaks were measured with a precision of 0.01 log D units and uncertainties were determined based on the width of the first two cross peak contours.

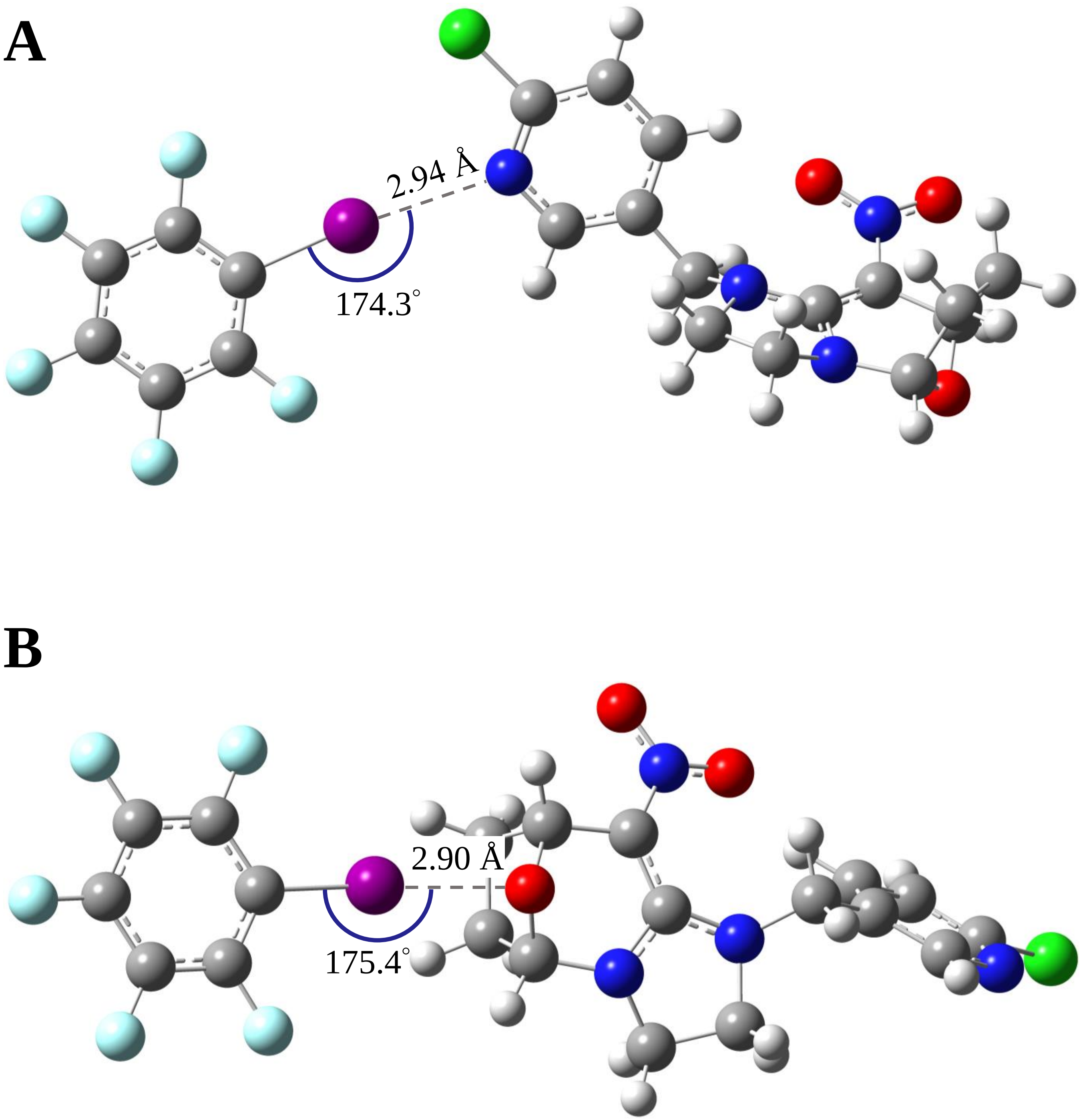

Figure S1. Geometry-optimized XB adducts of XB acceptor **cycloxaprid** ($C_{14}H_{15}ClN_4O_3$) at **(A)** N^1 and **(B)** O^1 with XB donor **iodopentafluorobenzene (IPFB)**.

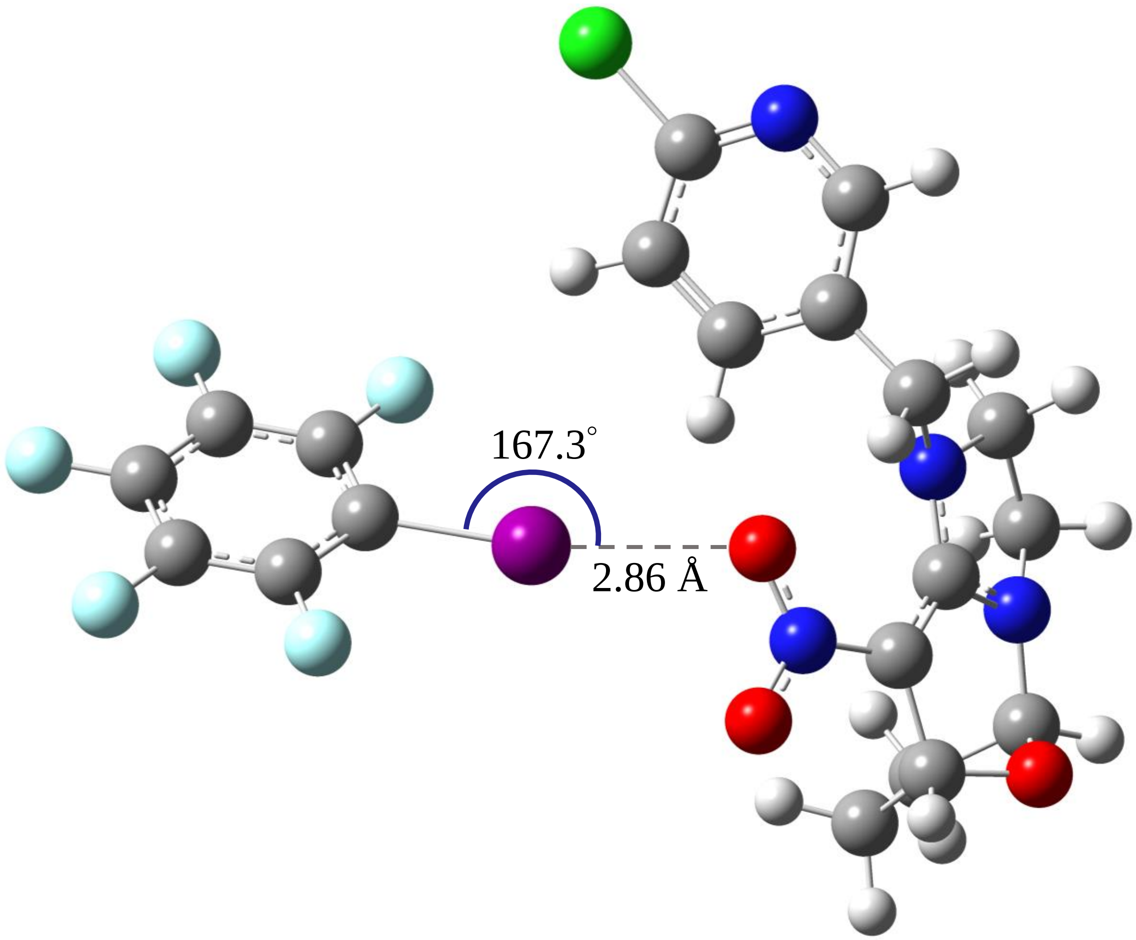

Figure S2. Geometry-optimized XB adducts of XB acceptor **cycloxaprid** ($C_{14}H_{15}ClN_4O_3$) at NO_2 with XB donor **iodopentafluorobenzene (IPFB)**.

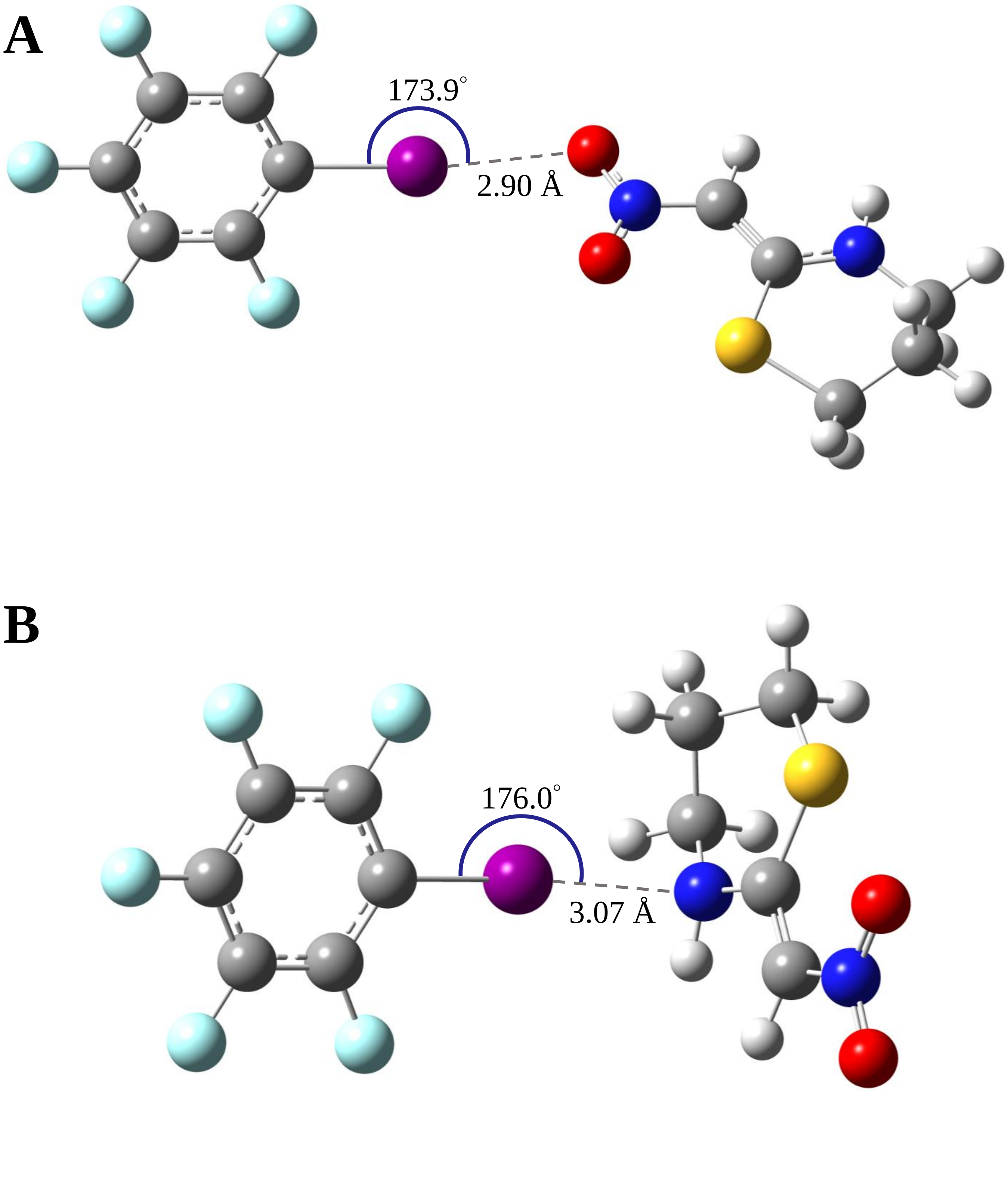

Figure S3. Geometry-optimized XB adducts of XB acceptor **nithiazine** ($C_5H_8N_2O_2S$) at **(A)** NO_2 and **(B)** N^1 with XB donor **iodopentafluorobenzene (IPFB)**.

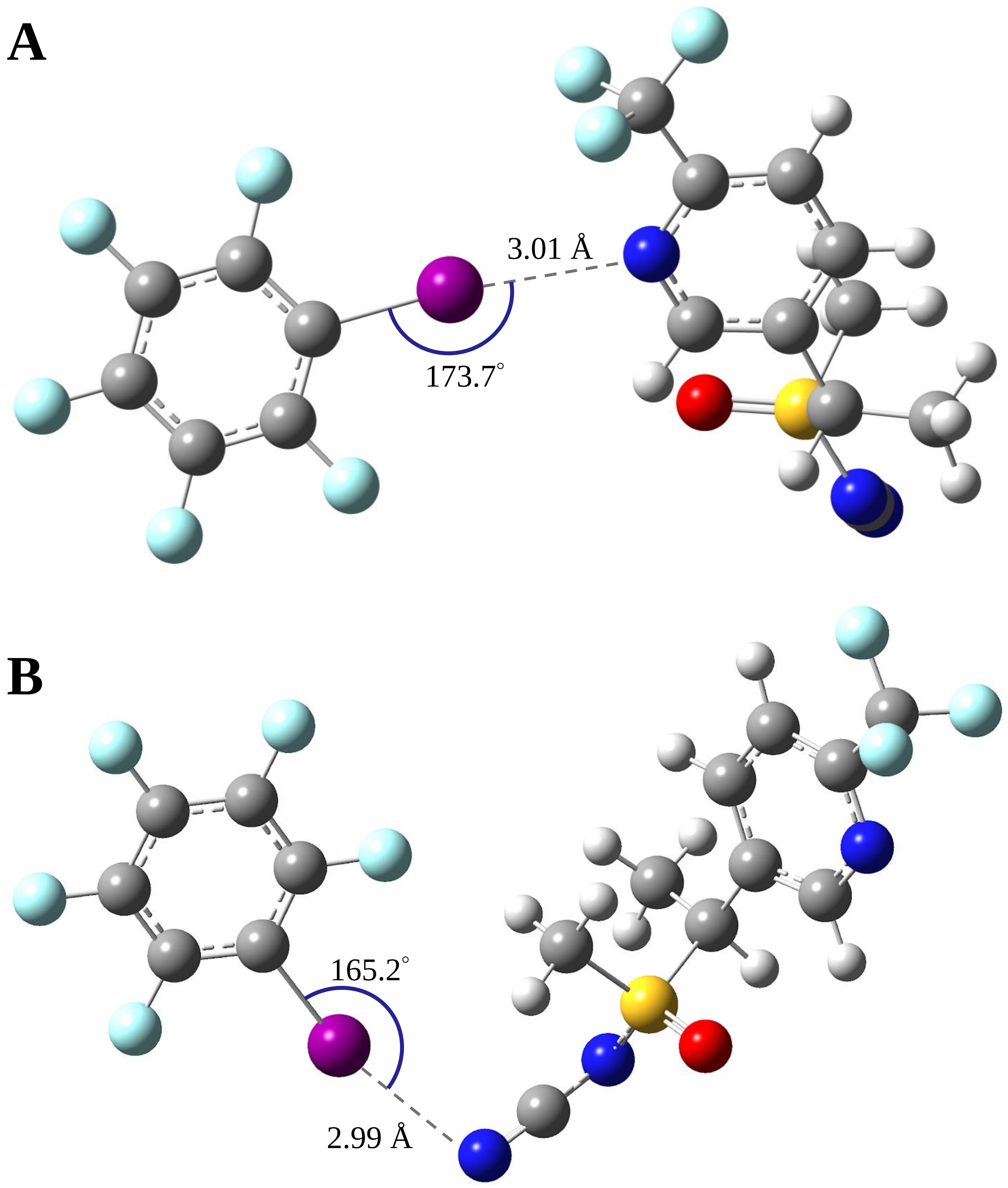

Figure S4. Geometry-optimized XB adducts of XB acceptor **sulfoxaflor** ($C_{10}H_{10}F_3N_3OS$) at **(A)** N^1 and **(B)** N^2 with XB donor **iodopentafluorobenzene (IPFB)**.

A

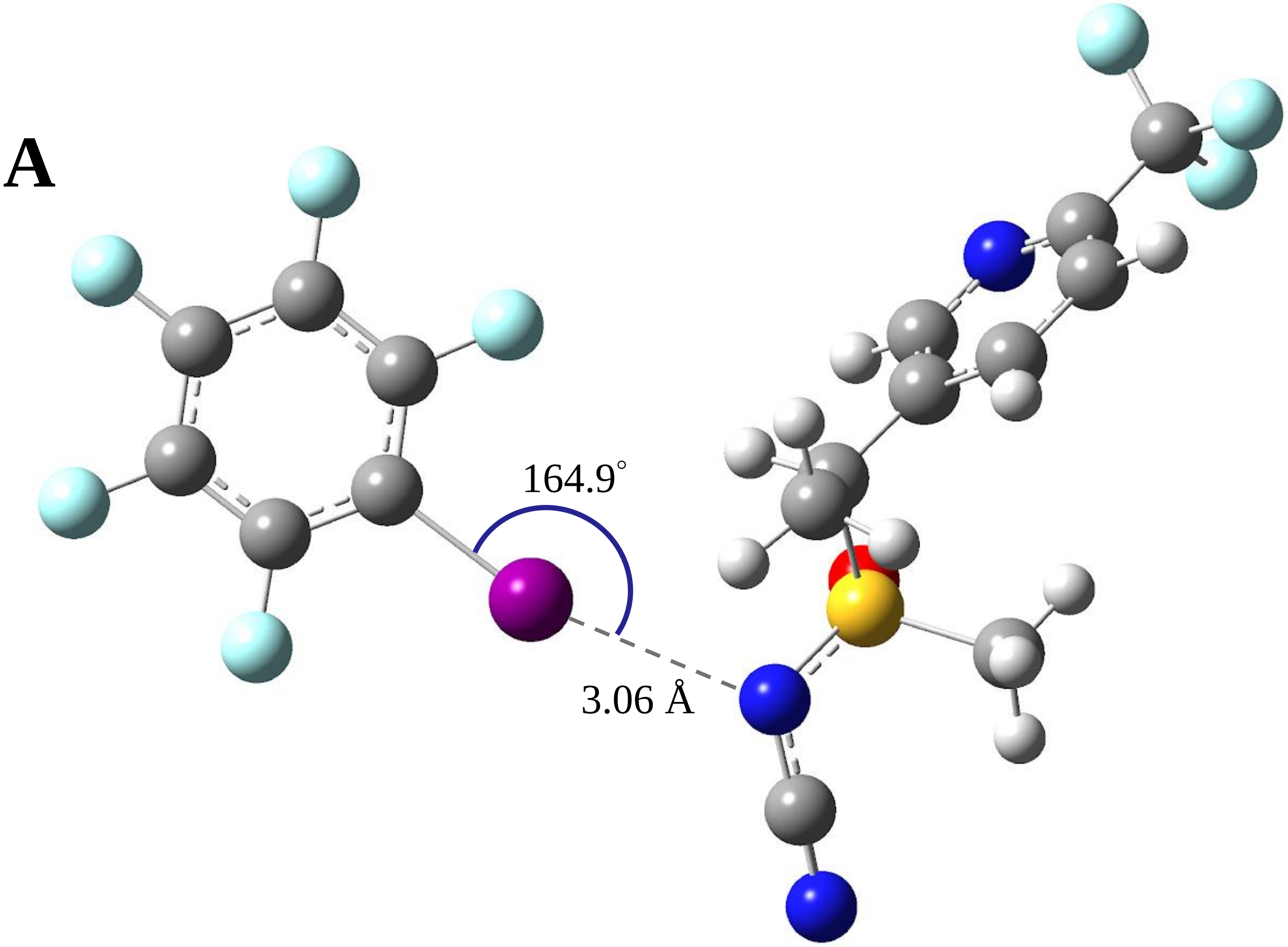

B

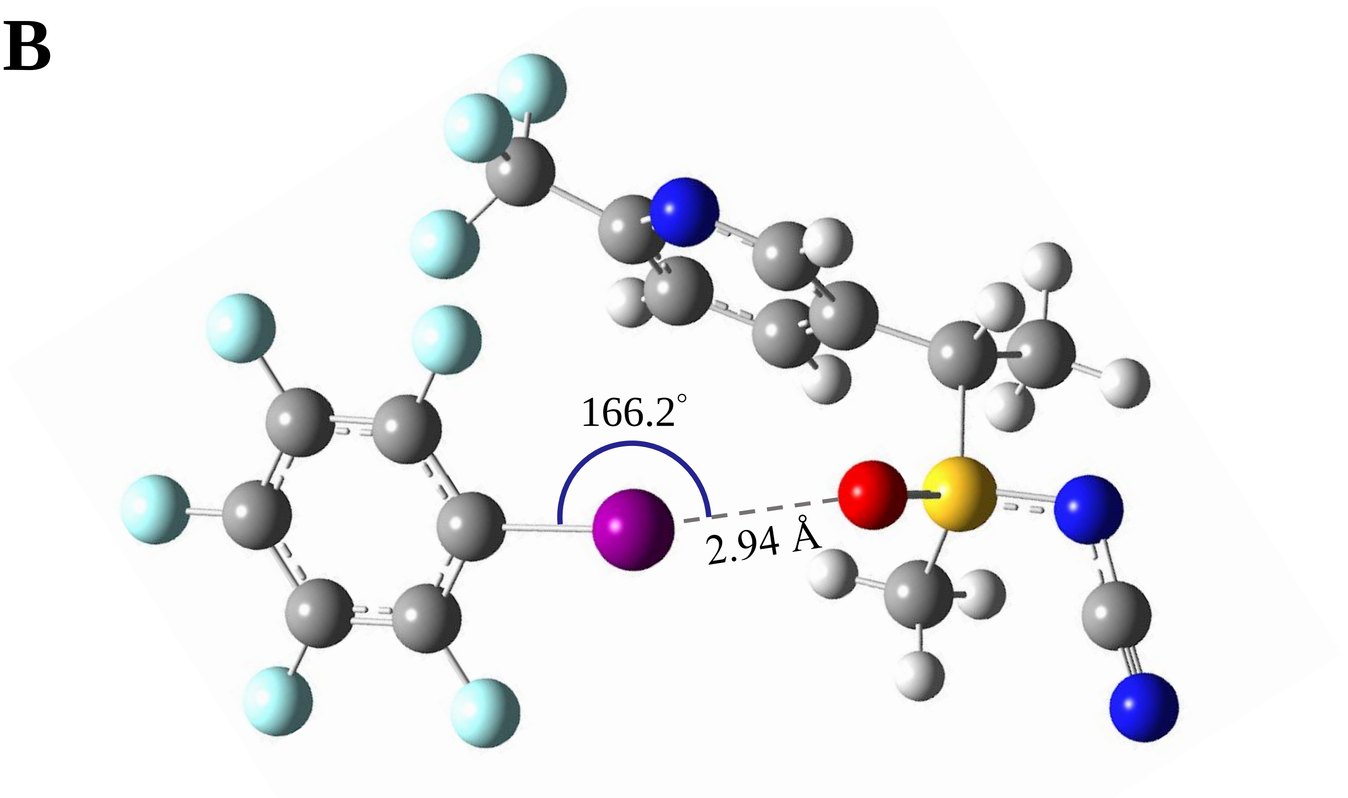

Figure S5. Geometry-optimized XB adducts of XB acceptor **sulfoxaflor** ($C_{10}H_{10}F_3N_3OS$) at (A) N^3 and (B) O^1 with XB donor **iodopentafluorobenzene (IPFB)**.

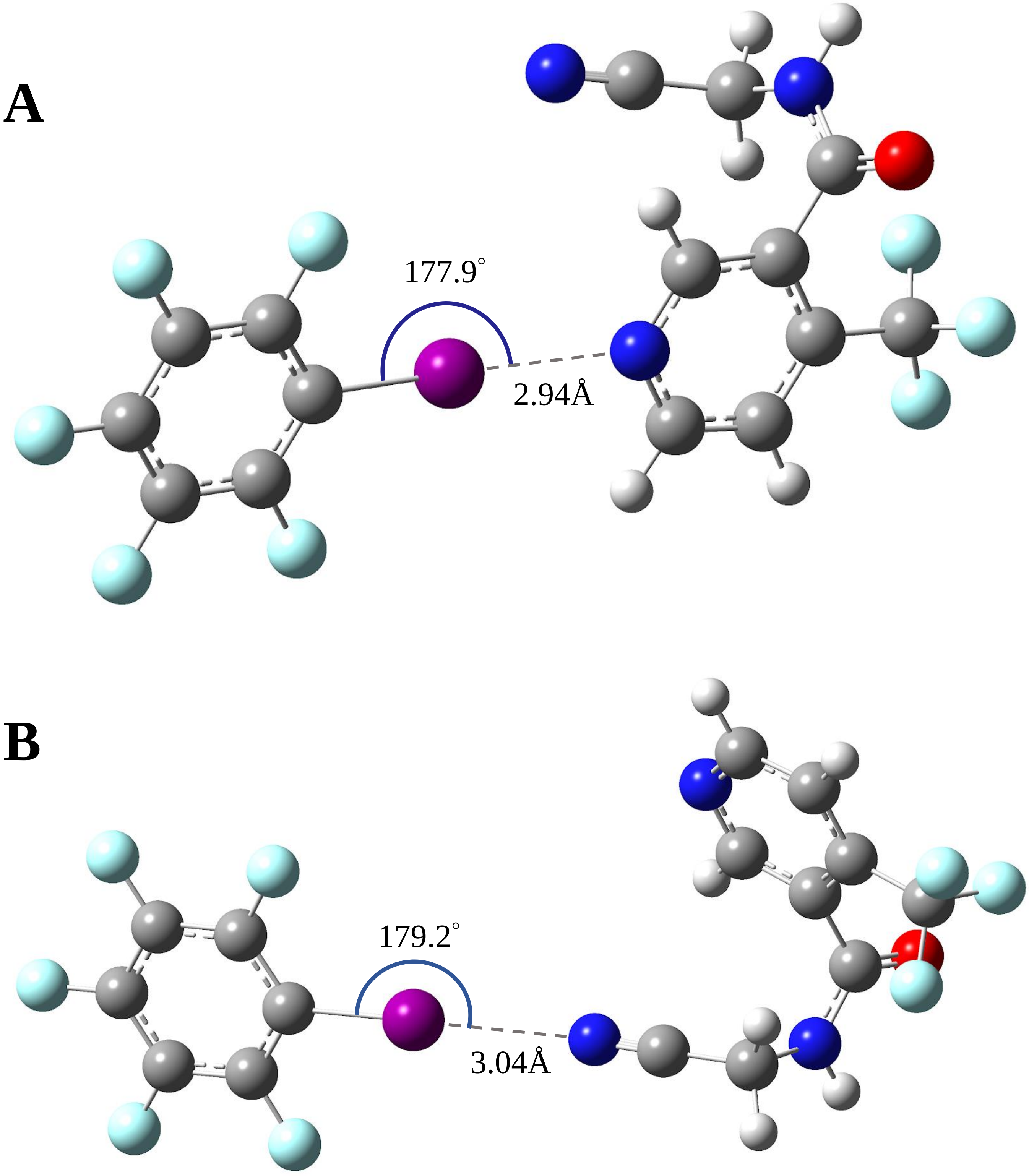

Figure S6. Geometry-optimized XB adducts of XB acceptor **flonicamid** ($C_9H_6F_3N_3O$) at **(A)** N^1 and **(B)** N^2 with XB donor **iodopentafluorobenzene (IPFB)**.

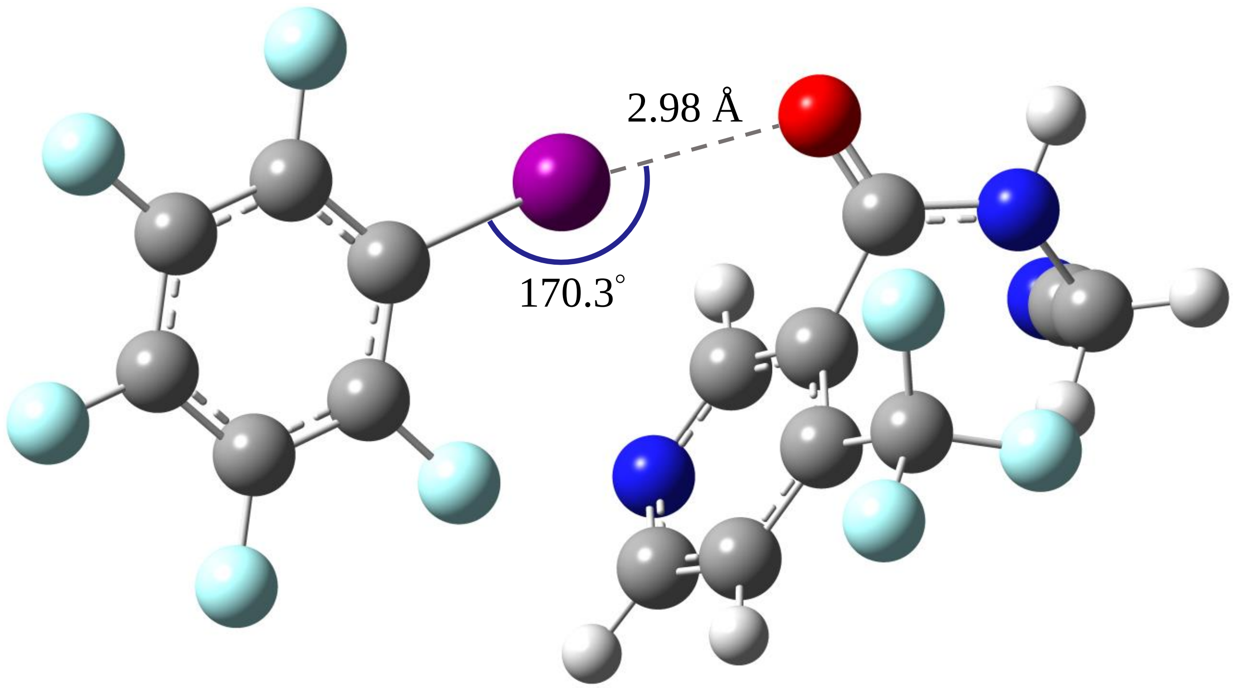

Figure S7. Geometry-optimized XB adducts of XB acceptor **flonicamid** ($C_9H_6F_3N_3O$) at O^1 with XB donor **iodopentafluorobenzene (IPFB)**.

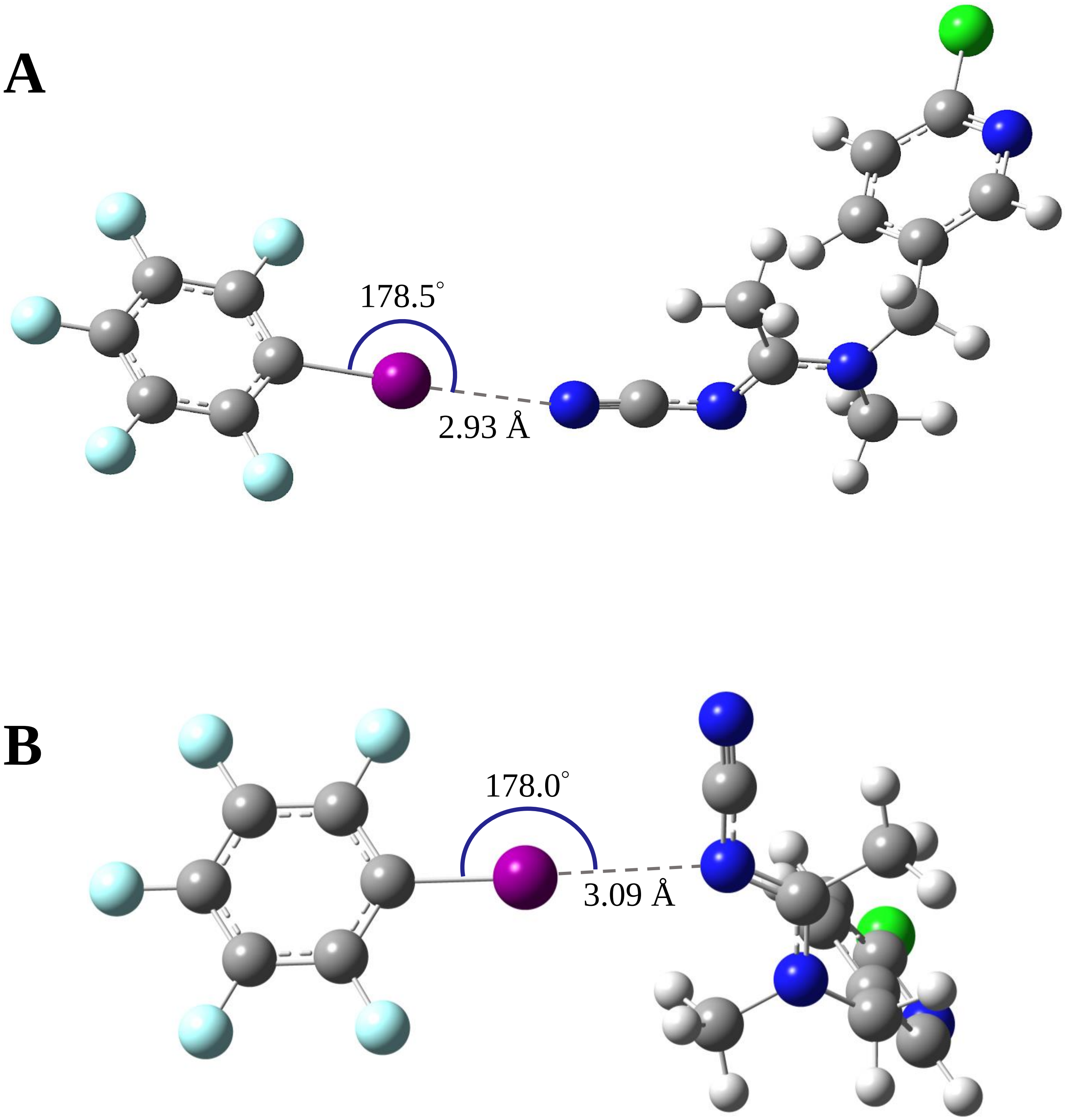

Figure S8. Geometry-optimized XB adducts of XB acceptor **acetamiprid** ($C_{10}H_{11}ClN_4$) at **(A)** N^1 and **(B)** N^2 with XB donor **iodopentafluorobenzene (IPFB)**.

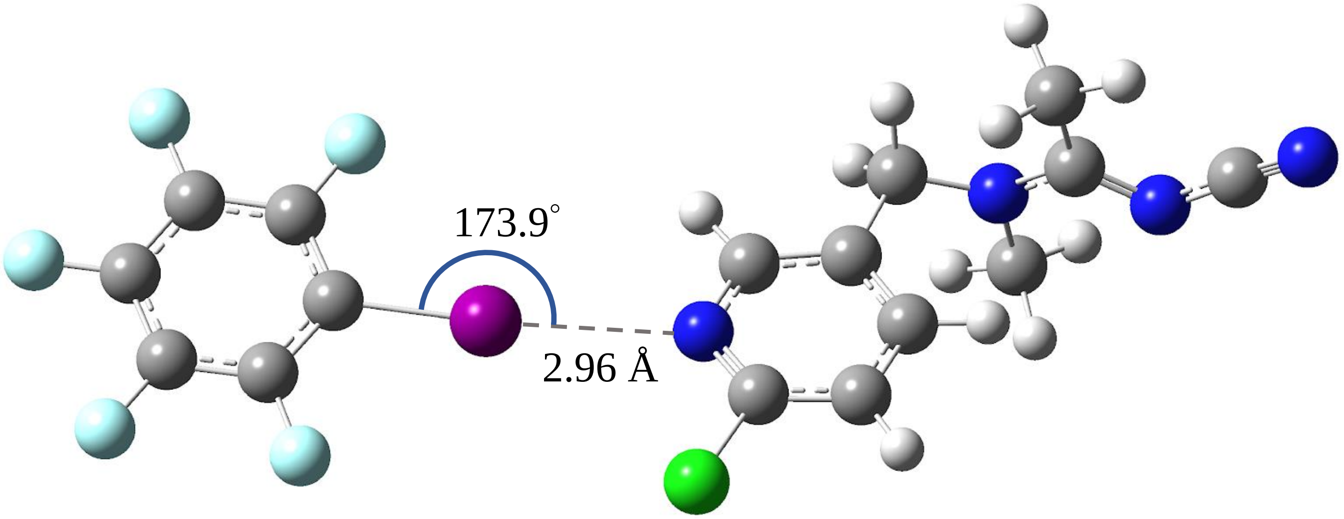

Figure S9. Geometry-optimized XB adducts of XB acceptor **acetamiprid** ($C_{10}H_{11}ClN_4$) at N^3 with XB donor **iodopentafluorobenzene (IPFB)**.

A

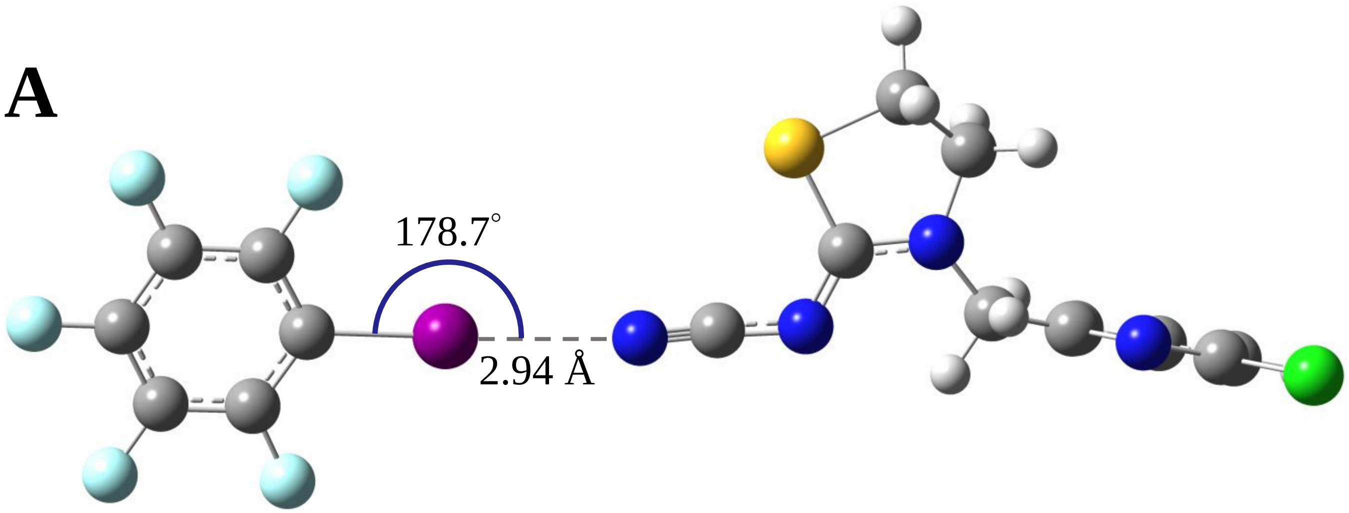

B

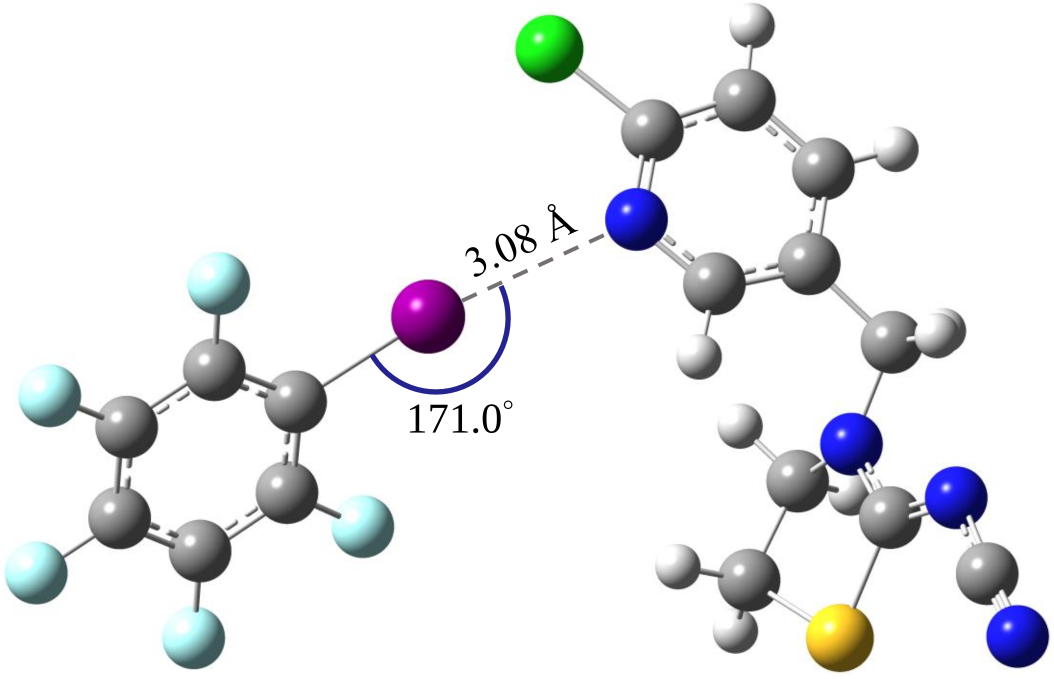

Figure S10. Geometry-optimized XB adducts of XB acceptor **thiacloprid** ($C_{10}H_9ClN_4S$) at **(A)** N^1 and **(B)** N^2 with XB donor **iodopentafluorobenzene (IPFB)**.

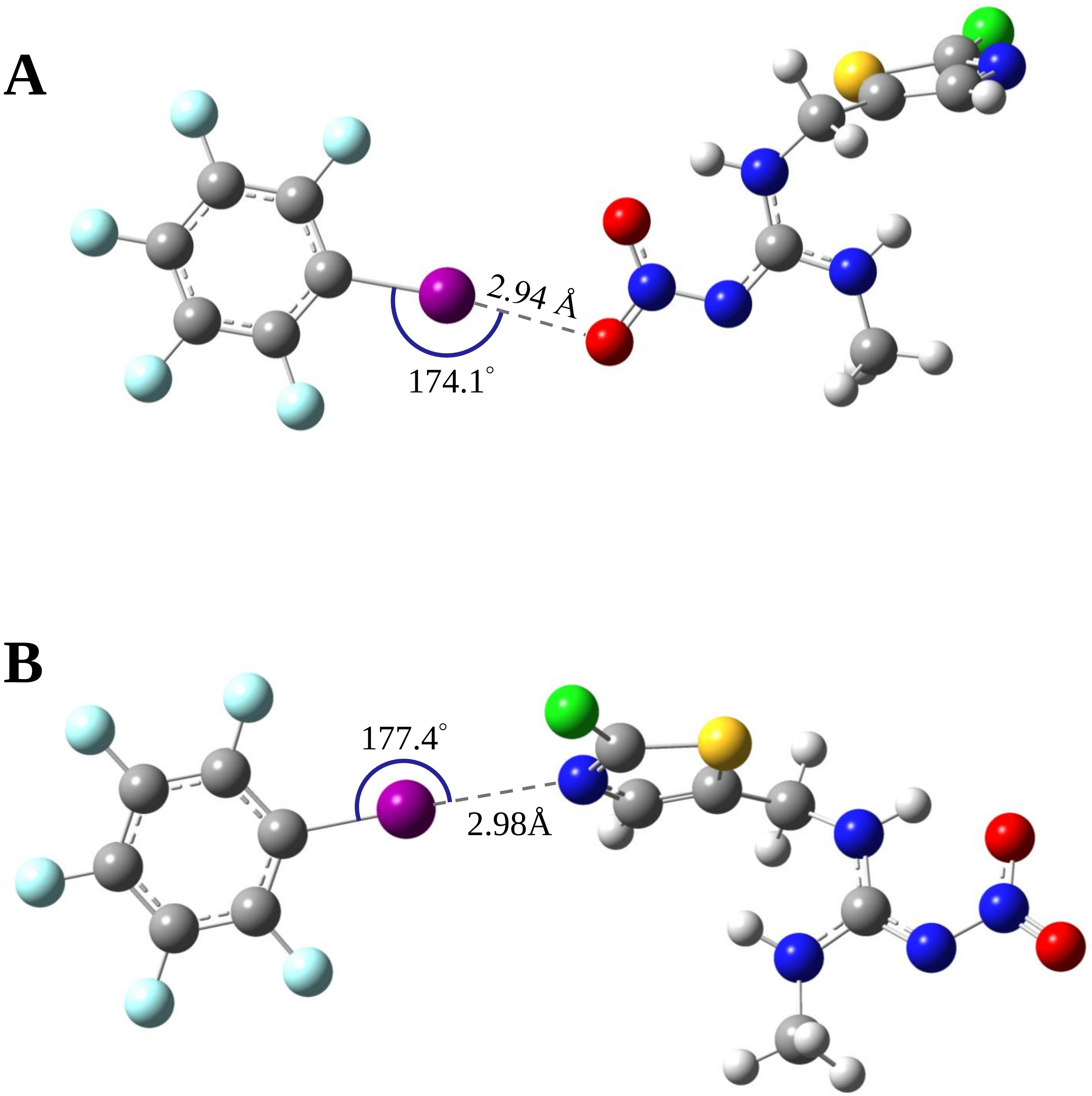

Figure S11. Geometry-optimized XB adducts of XB acceptor **clothianidin** ($C_6H_8ClN_5O_2S$) at **(A)** NO_2 and **(B)** N^1 with XB donor **iodopentafluorobenzene (IPFB)**.

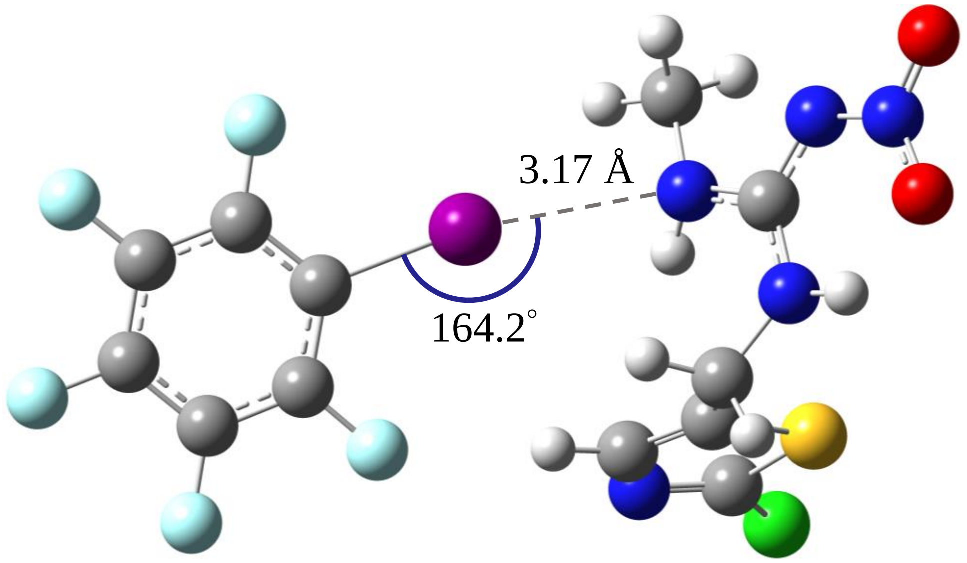

Figure S12. Geometry-optimized XB adducts of XB acceptor **clothianidin** ($C_6H_8ClN_5O_2S$) at N^2 with XB donor **iodopentafluorobenzene (IPFB)**.

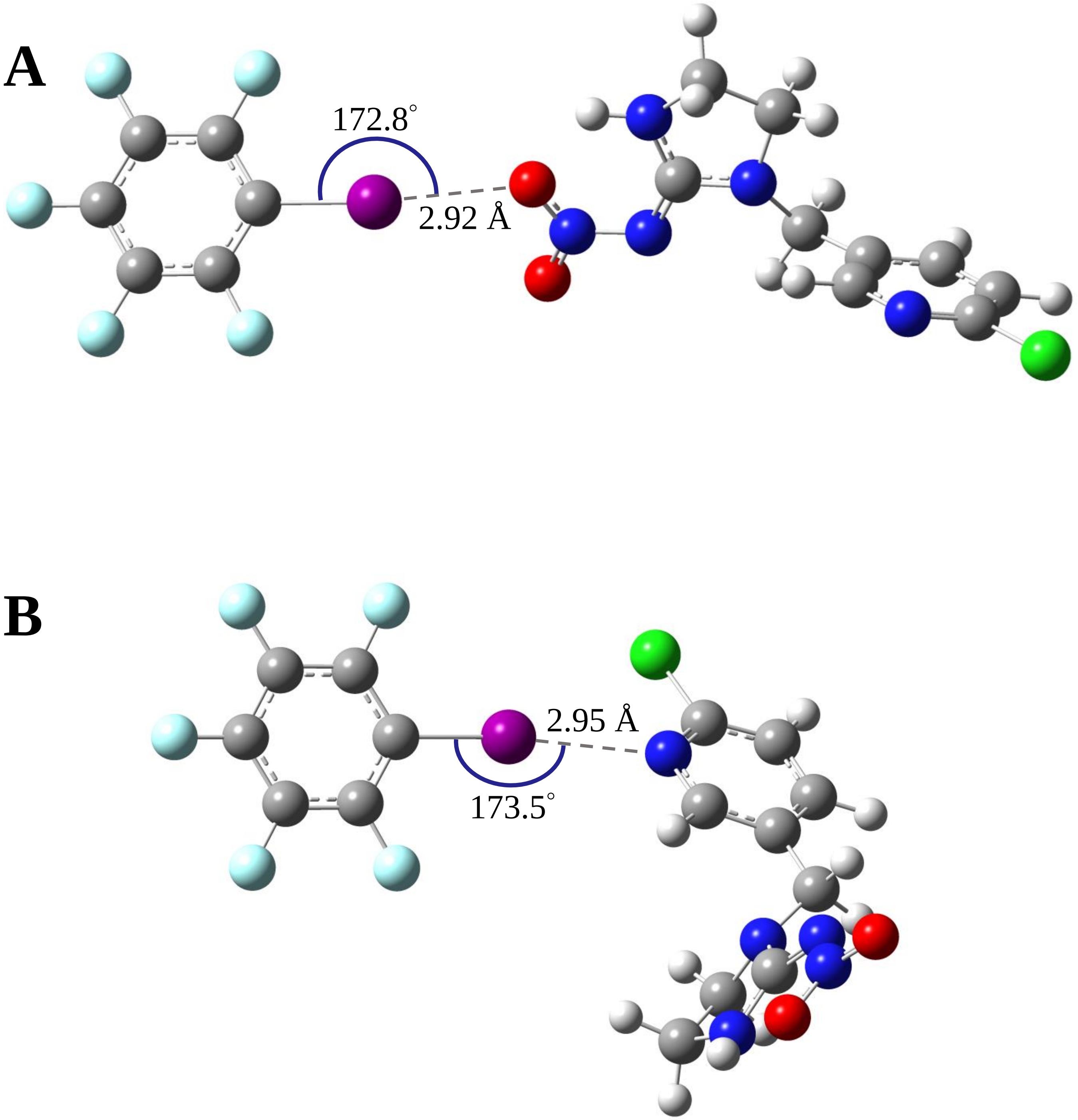

Figure S13. Geometry-optimized XB adducts of XB acceptor **imidacloprid** ($C_9H_{10}ClN_5O_2$) at **(A)** NO_2 and **(B)** N^1 with XB donor **iodopentafluorobenzene (IPFB)**.

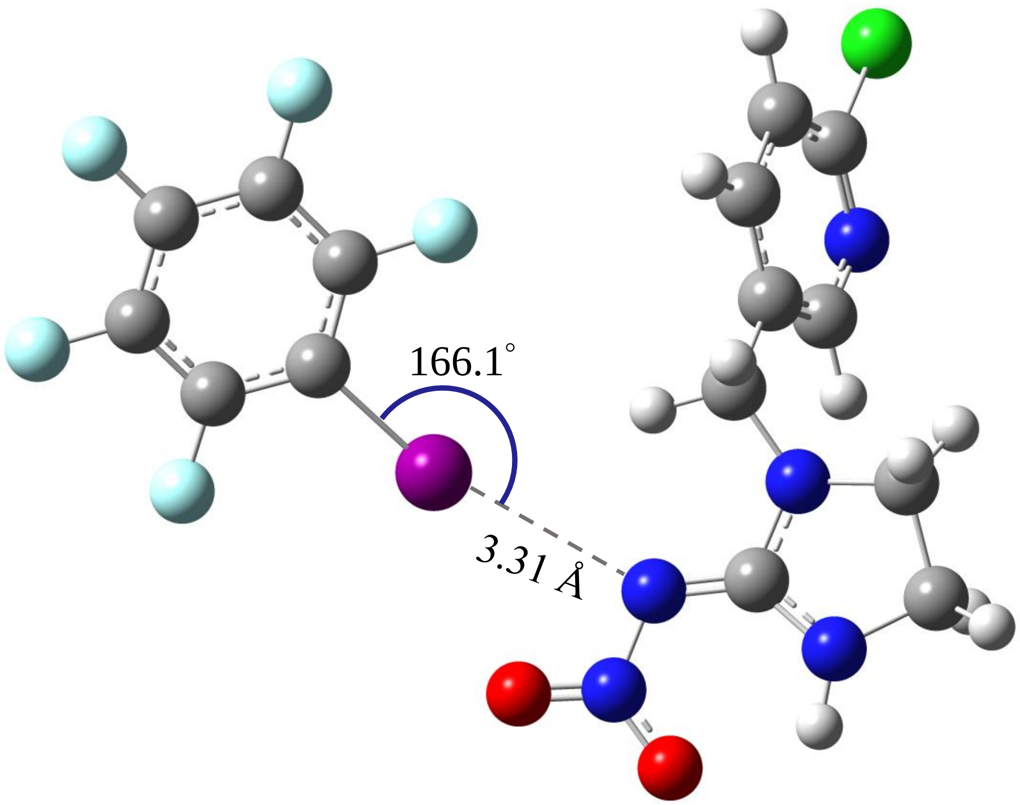

Figure S14. Geometry-optimized XB adducts of XB acceptor **imidacloprid** ($C_9H_{10}ClN_5O_2$) at N^2 with XB donor **iodopentafluorobenzene (IPFB)**.

A

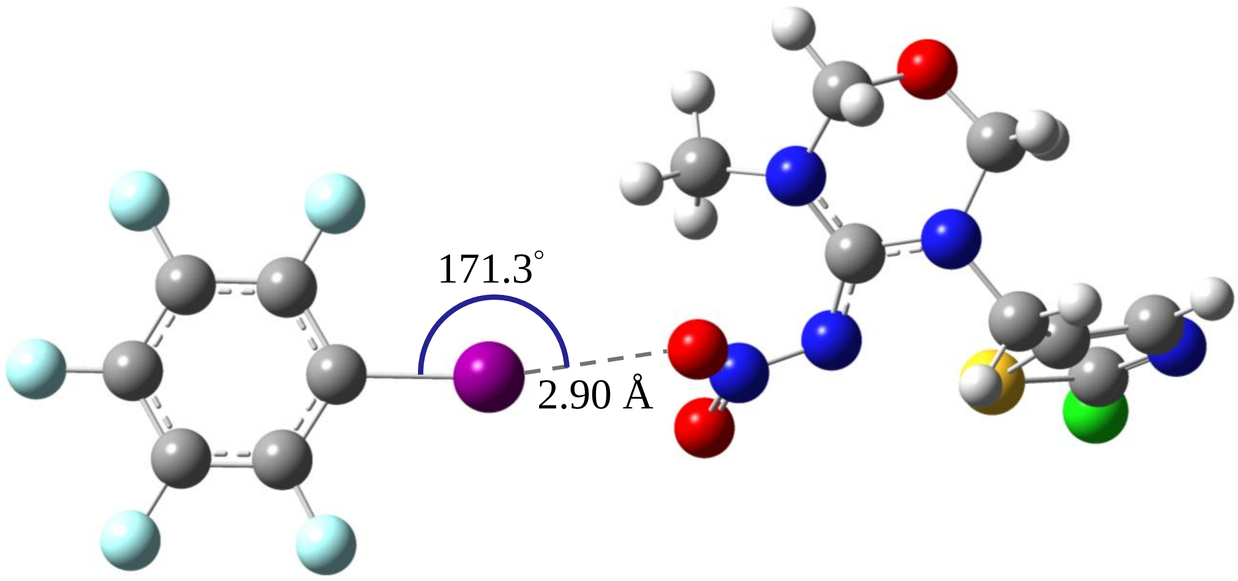

B

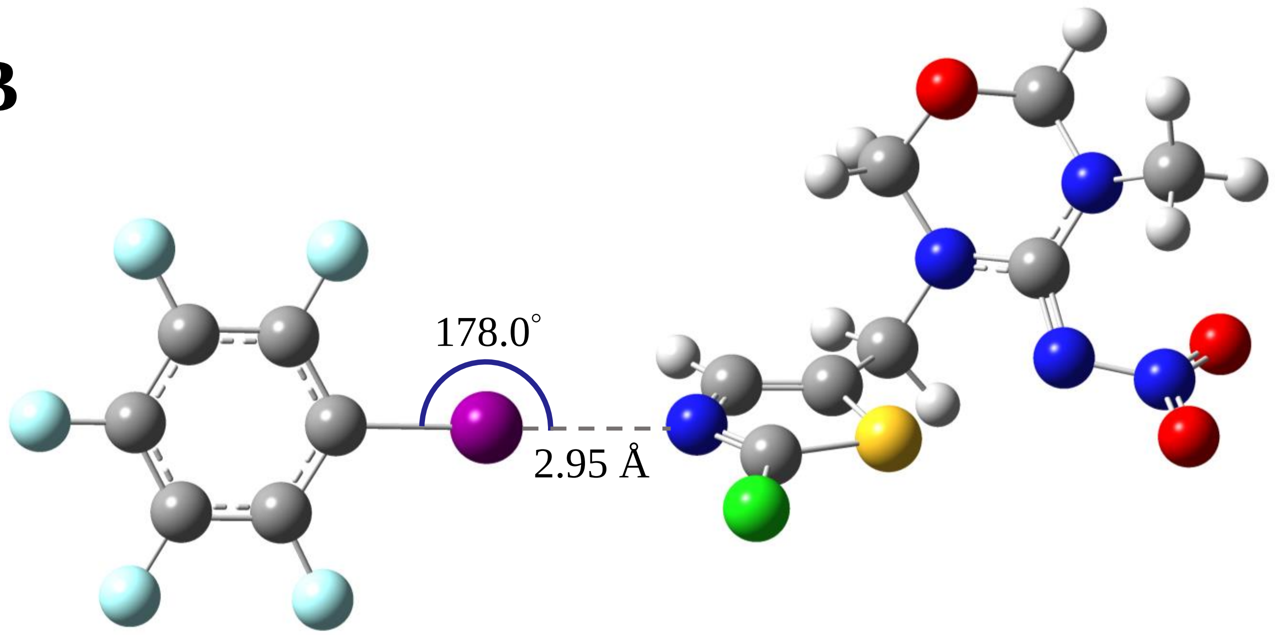

Figure S15. Geometry-optimized XB adducts of XB acceptor **thiamethoxam** ($C_8H_{10}ClN_5O_3$) at **(A)** NO_2 and **(B)** N^1 with XB donor **iodopentafluorobenzene (IPFB)**.

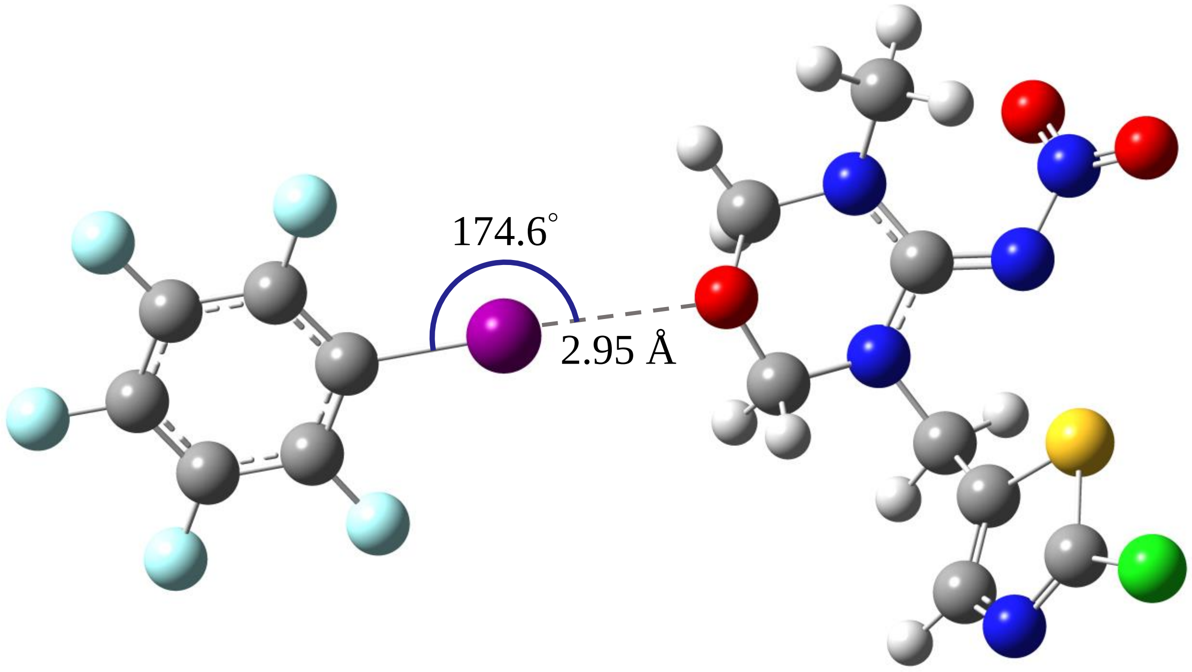

Figure S16. Geometry-optimized XB adducts of XB acceptor **thiamethoxam** ($C_8H_{10}ClN_5O_3$) at O^1 with XB donor **iodopentafluorobenzene (IPFB)**.

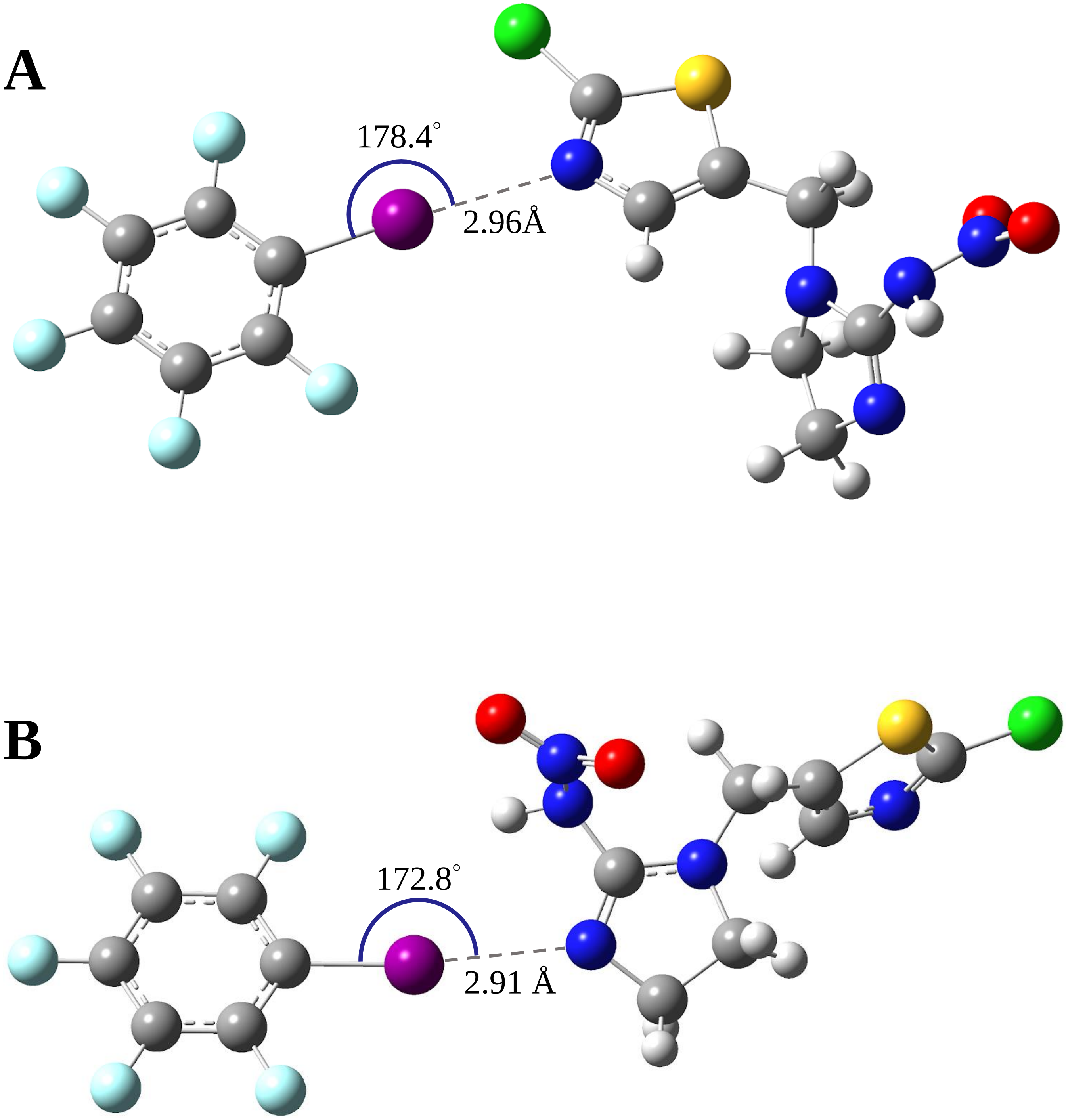

Figure S17. Geometry-optimized XB adducts of XB acceptor **imidaclothiz** ($C_7H_8ClN_5O_2S$) at **(A)** N^1 and **(B)** N^2 with XB donor **iodopentafluorobenzene (IPFB)**.

A

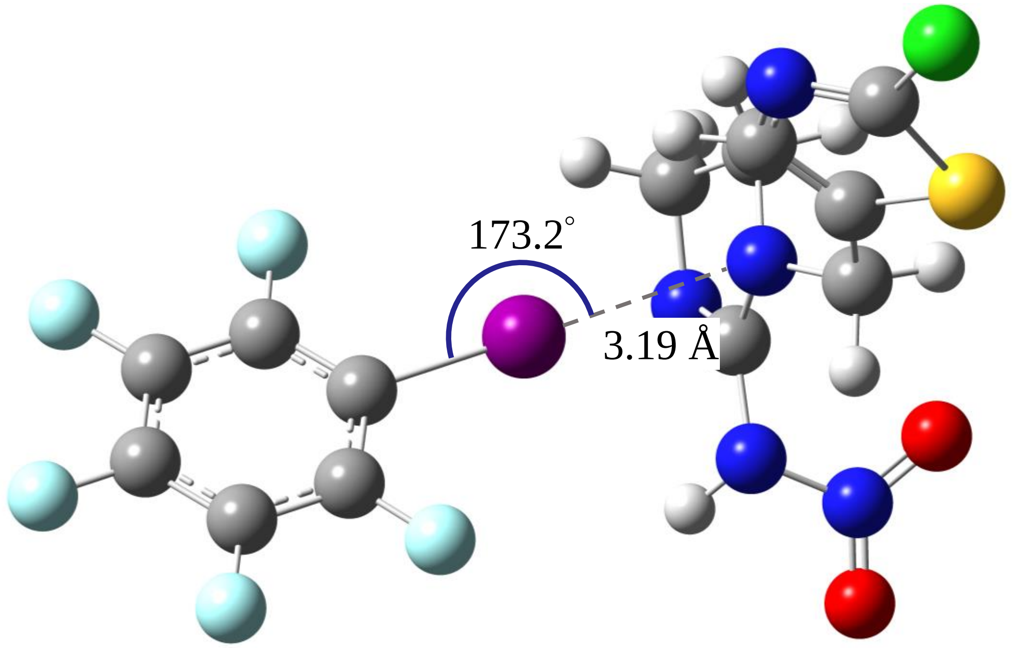

B

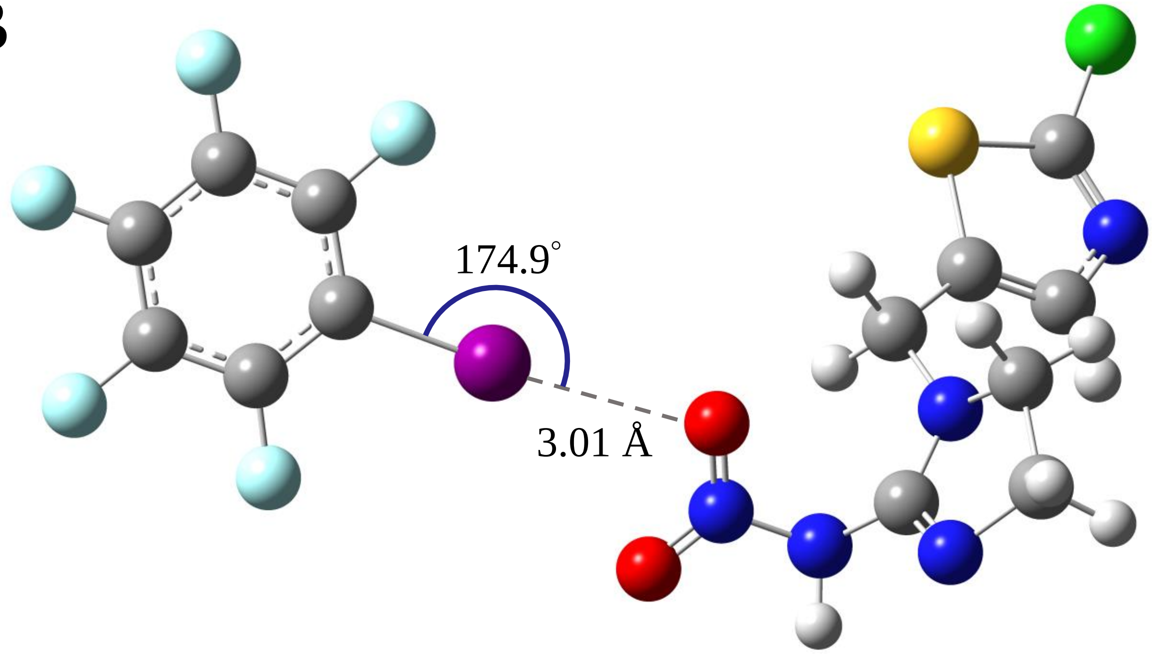

Figure S18. Geometry-optimized XB adducts of XB acceptor **imidaclothiz** ($C_7H_8ClN_5O_2S$) at **(A)** N^3 and **(B)** NO_2 with XB donor **iodopentafluorobenzene (IPFB)**.

A

B

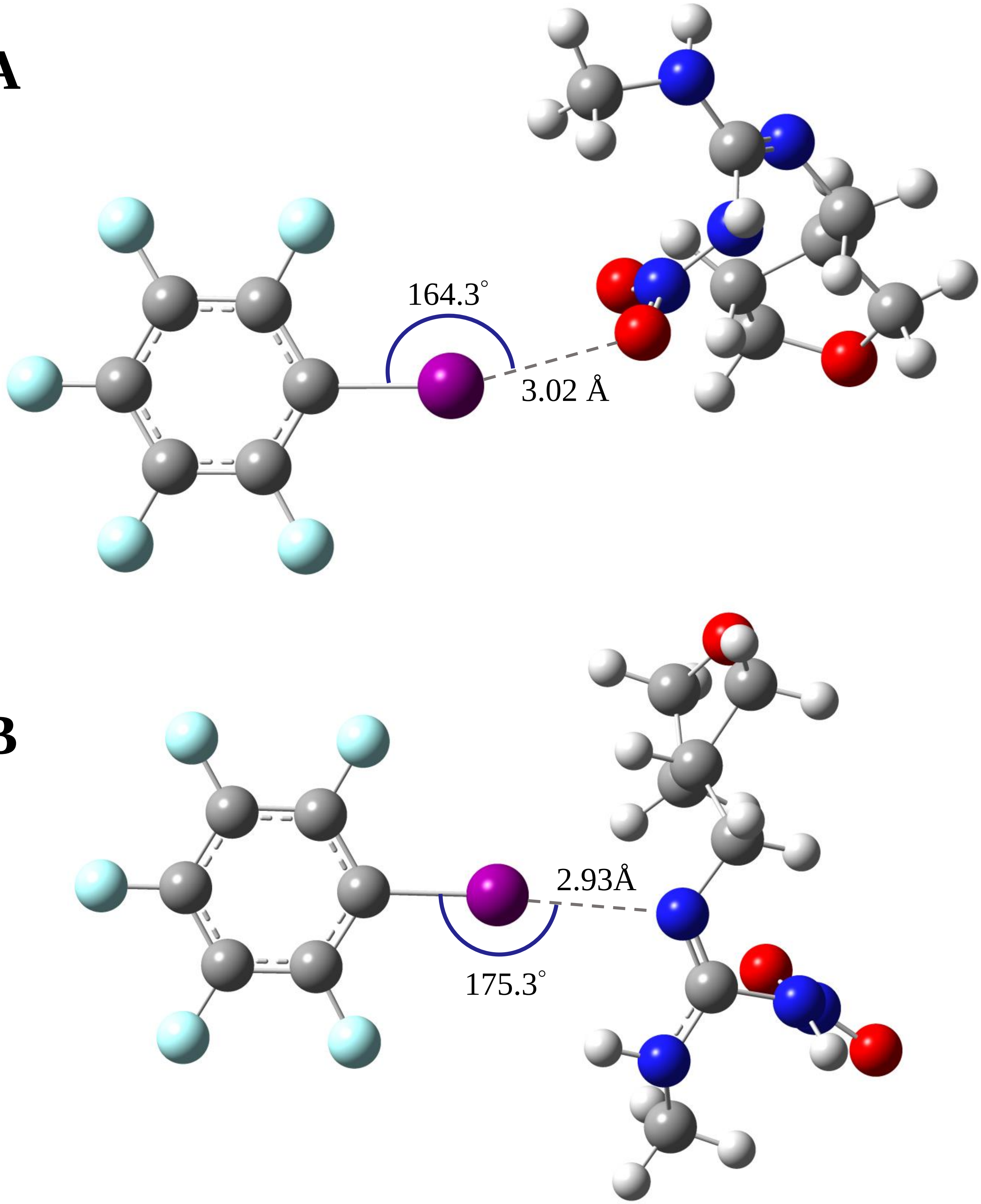

Figure S19. Geometry-optimized XB adducts of XB acceptor **dinotefuran** ($C_7H_{14}N_4O_3$) at **(A)** NO_2 and **(B)** N^1 with XB donor **iodopentafluorobenzene (IPFB)**.

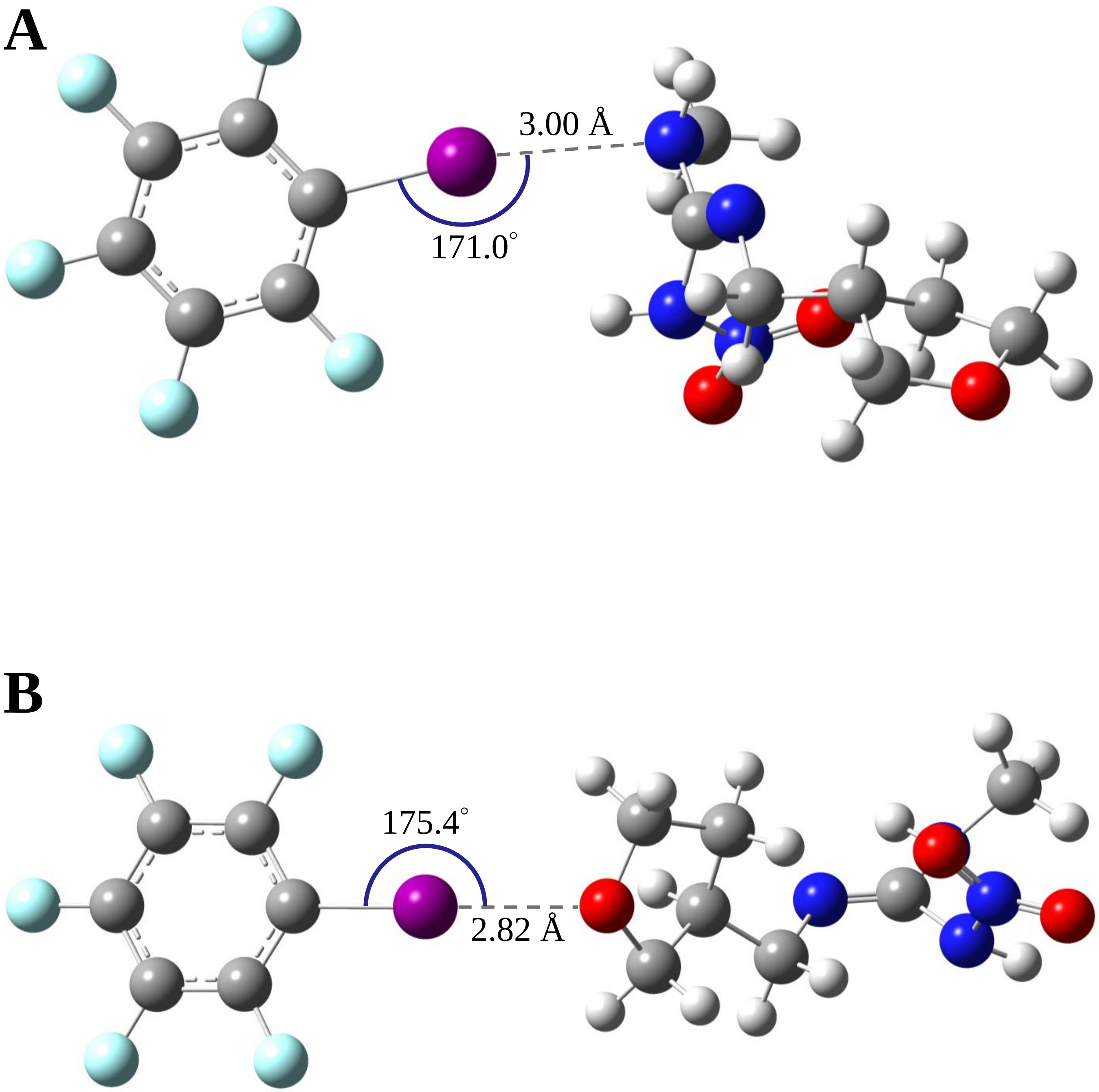

Figure S20. Geometry-optimized XB adducts of XB acceptor **dinotefuran** ($C_7H_{14}N_4O_3$) at **(A)** N^2 and **(B)** O^1 with XB donor **iodopentafluorobenzene (IPFB)**.

Table S1. Diffusion-Ordered Spectroscopy (DOSY) NMR Measurements of Diffusion Coefficients (D) for XB Interactions of Thiamethoxam with XB Donors and Control Systems.

#	XB Acceptor	Solvent	XB Donor	Target	Log D ($\log(m^2/sec)$)
S-1	Thiamethoxam (70 mM)	THF-d_8	None	^1H Thiamethoxam	-8.80 (±0.04)
S-2			IPFB (70 mM)		-8.85 (±0.04)
S-3			PFT (70 mM)		-8.80 (±0.08)
S-4			None	^1H THF-d_7h_1	-8.51 (±0.04)
S-5			IPFB (70 mM)		-8.53 (±0.03)
S-6			PFT (70 mM)		-8.51 (±0.02)

Notes: DOSY NMR measurements were carried out at 15 minutes after sample preparation.

Table S2. Diffusion-Ordered Spectroscopy (DOSY) NMR Measurements of Diffusion Coefficients (D) of Thiamethoxam in the Presence of *f*-MPCs and u*nf*-MPCs.

#	XB Acceptor	Solvent	XB Donor	Target	Log D ($\log(m^2/sec)$)
S-7	Thiamethoxam (70 mM)	THF-d_8	*unf*-MPCs	^1H Thiamethoxam	-8.72 (±0.03)
S-8			*f*-MPCs		-8.76 (±0.04)
S-9			*unf*-MPCs	^1H THF-d_7h_1	-8.48 (±0.05)
S-10			*f*-MPCs		-8.48 (±0.06)

Notes: Concentrations of *unf*-MPCs and *f*-MPCs are equivalent to A_{518} = 0.50 a.u. DOSY NMR measurements of the sample of each NN with either *unf*-MPCs or *f*-MPCs were carried out at 15 minutes after sample preparation.

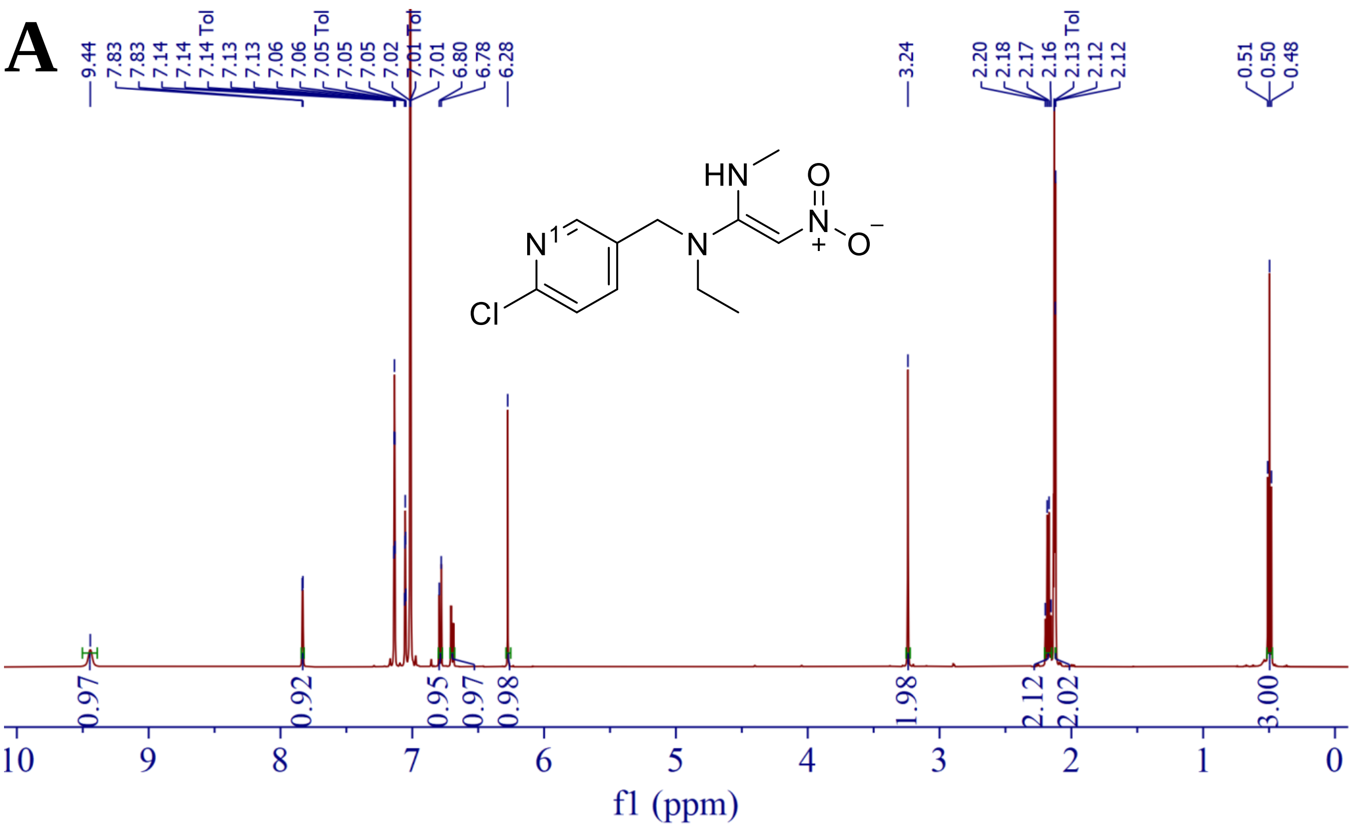

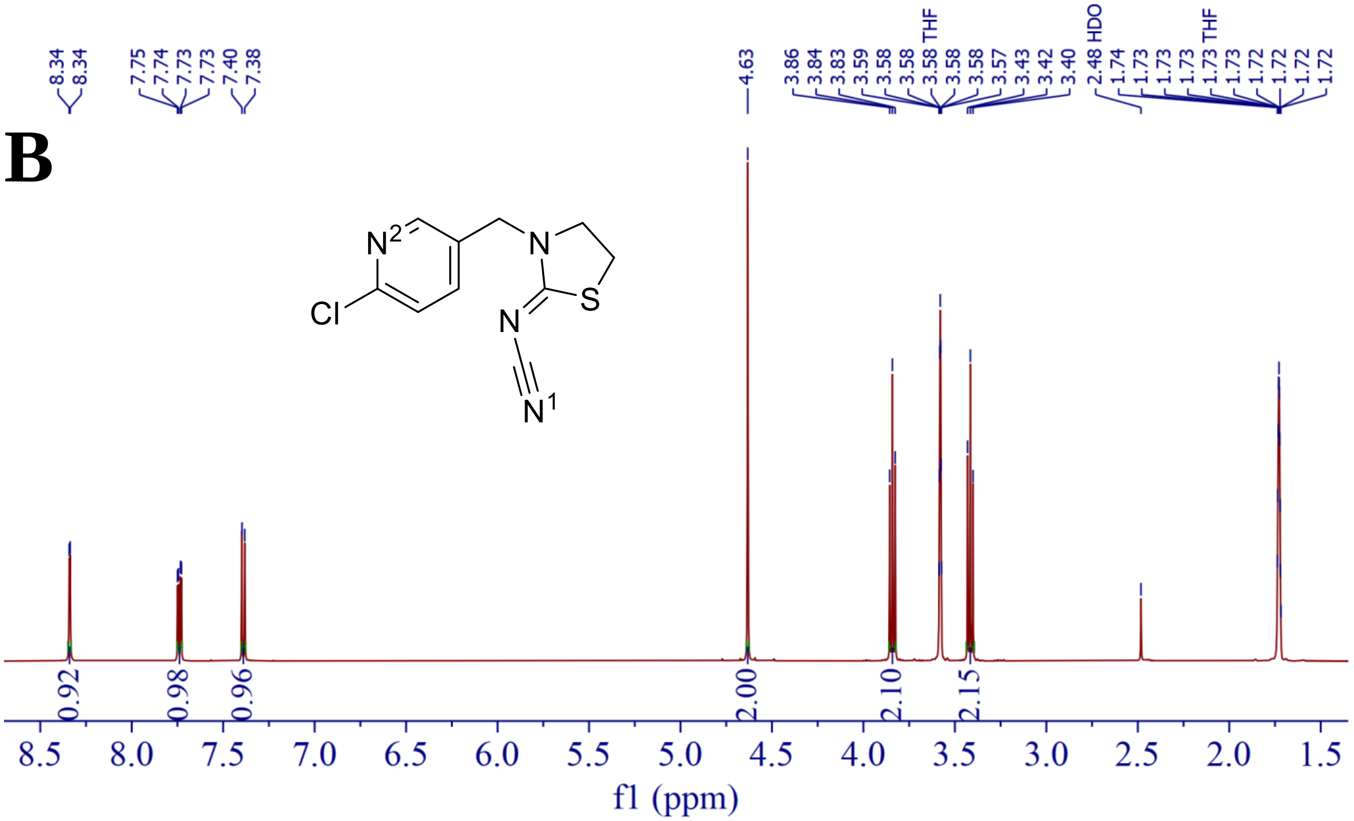

Figure S21. (A) ^{1}H NMR (400 MHz) spectra of **(A)** nitenpyram (in toluene-d_8) and **(B)** thiacloprid (in tetrahydrofuran-d_8) at 298K.

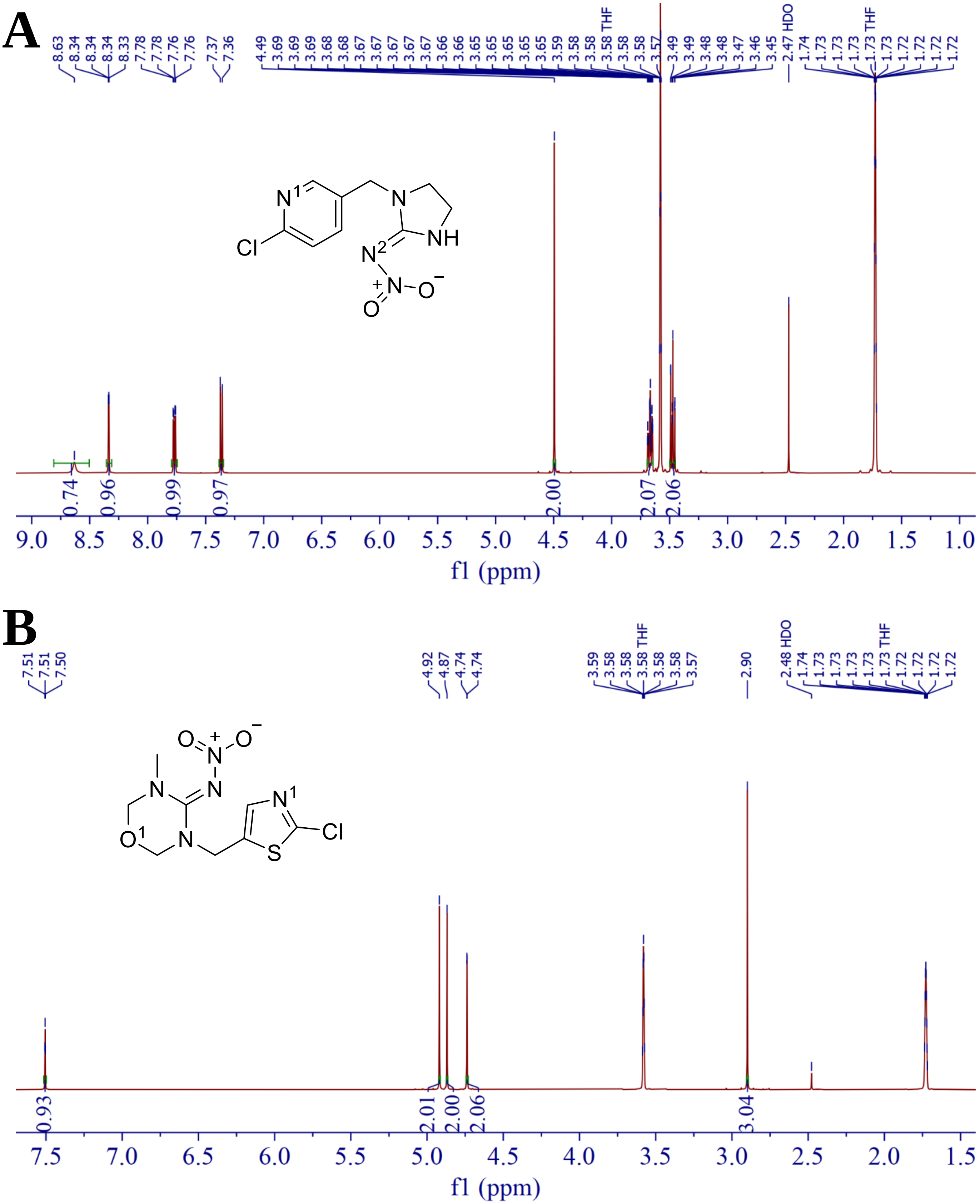

Figure S22. **(A)** ^{1}H NMR (400 MHz) spectra of **(A)** imidacloprid and **(B)** thiamethoxam in tetrahydrofuran-d_8 at 298K.

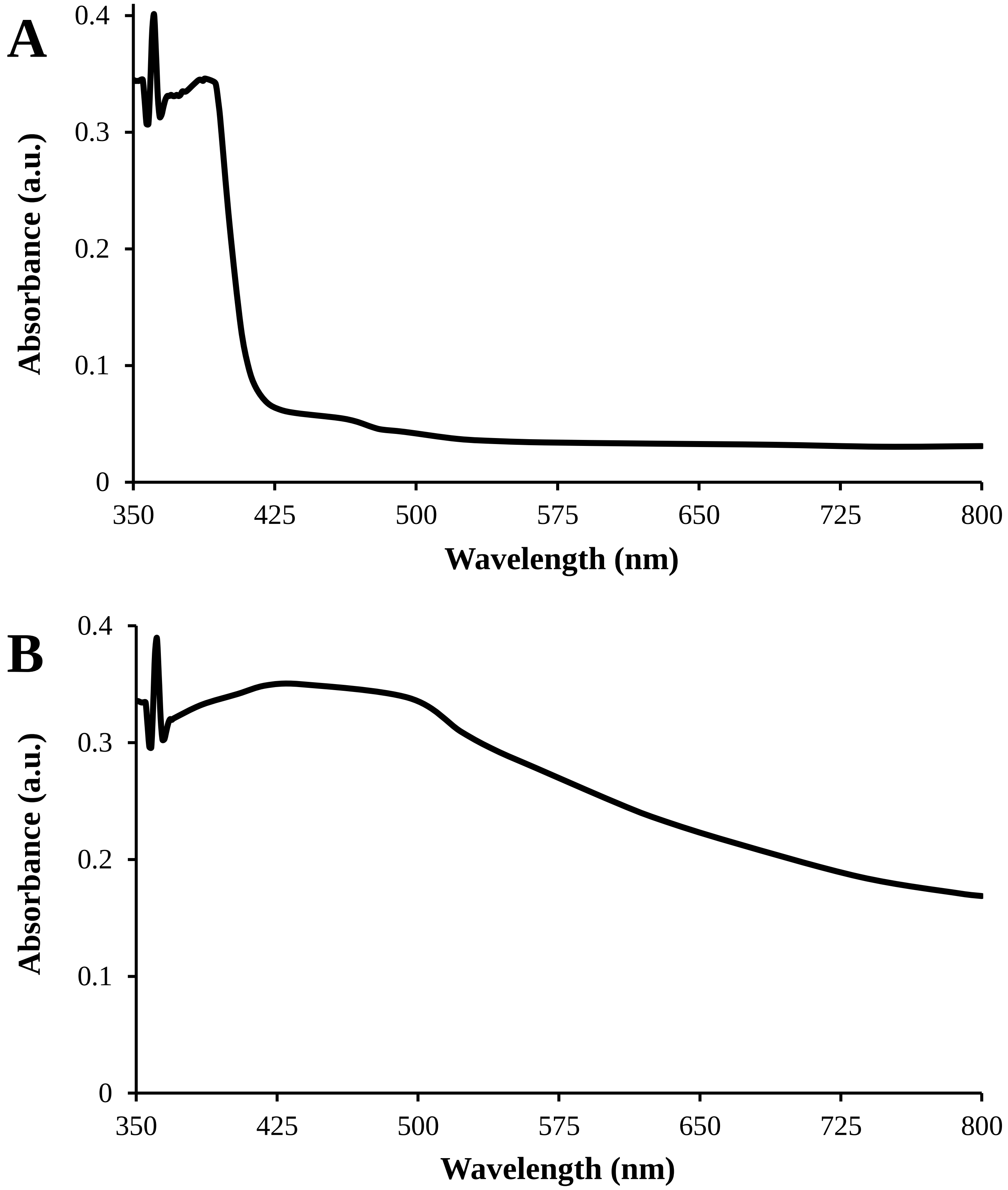

Figure S23. UV-Vis spectra of **nitenpyram** at **(A)** 11 mM in toluene and **(B)** 246 mM in tetrahydrofuran.

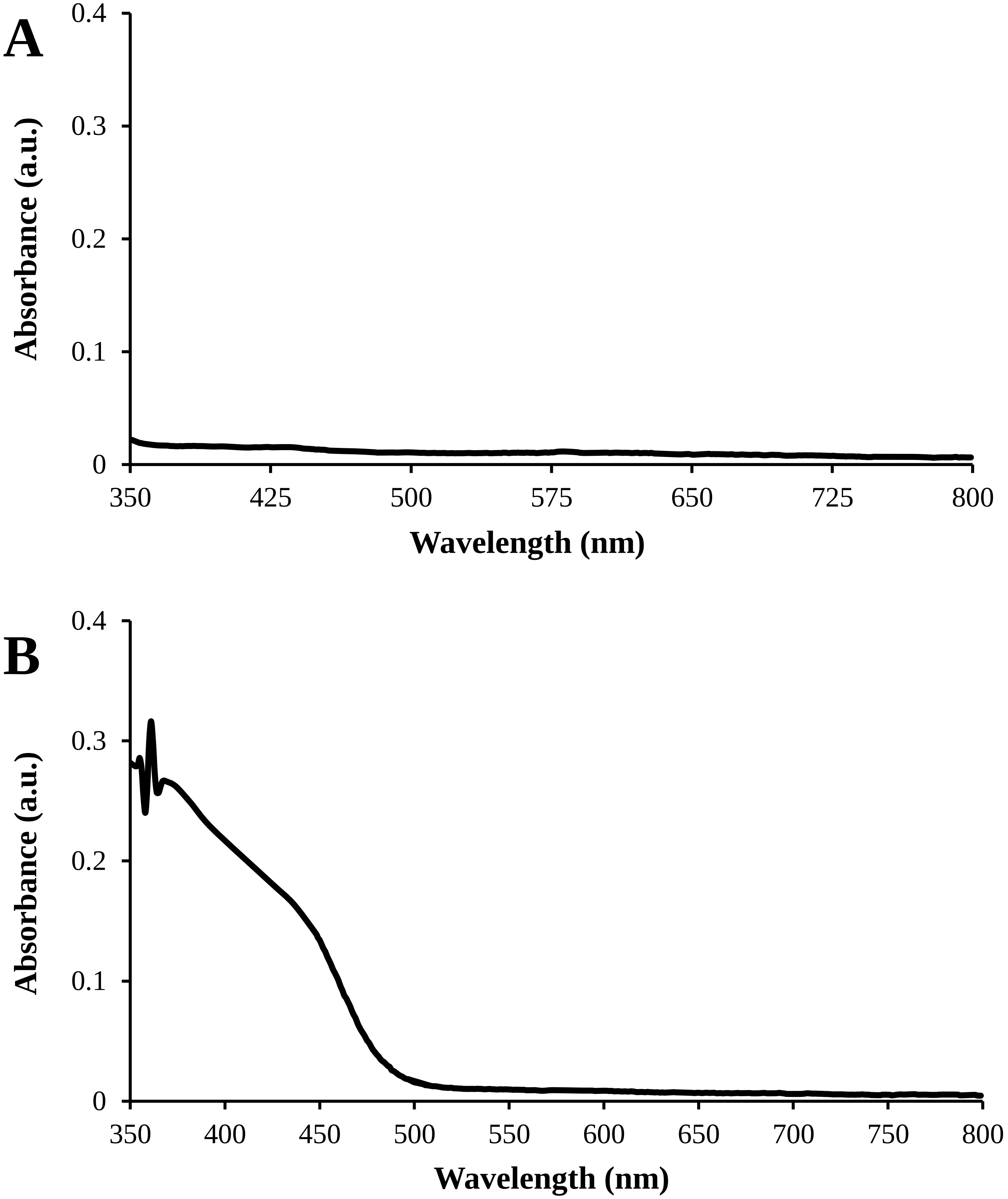

Figure S24. UV-Vis spectra of **(A) sulfoxaflor** (72 mM) and **(B) acetamiprid** (75 mM) in tetrahydrofuran.

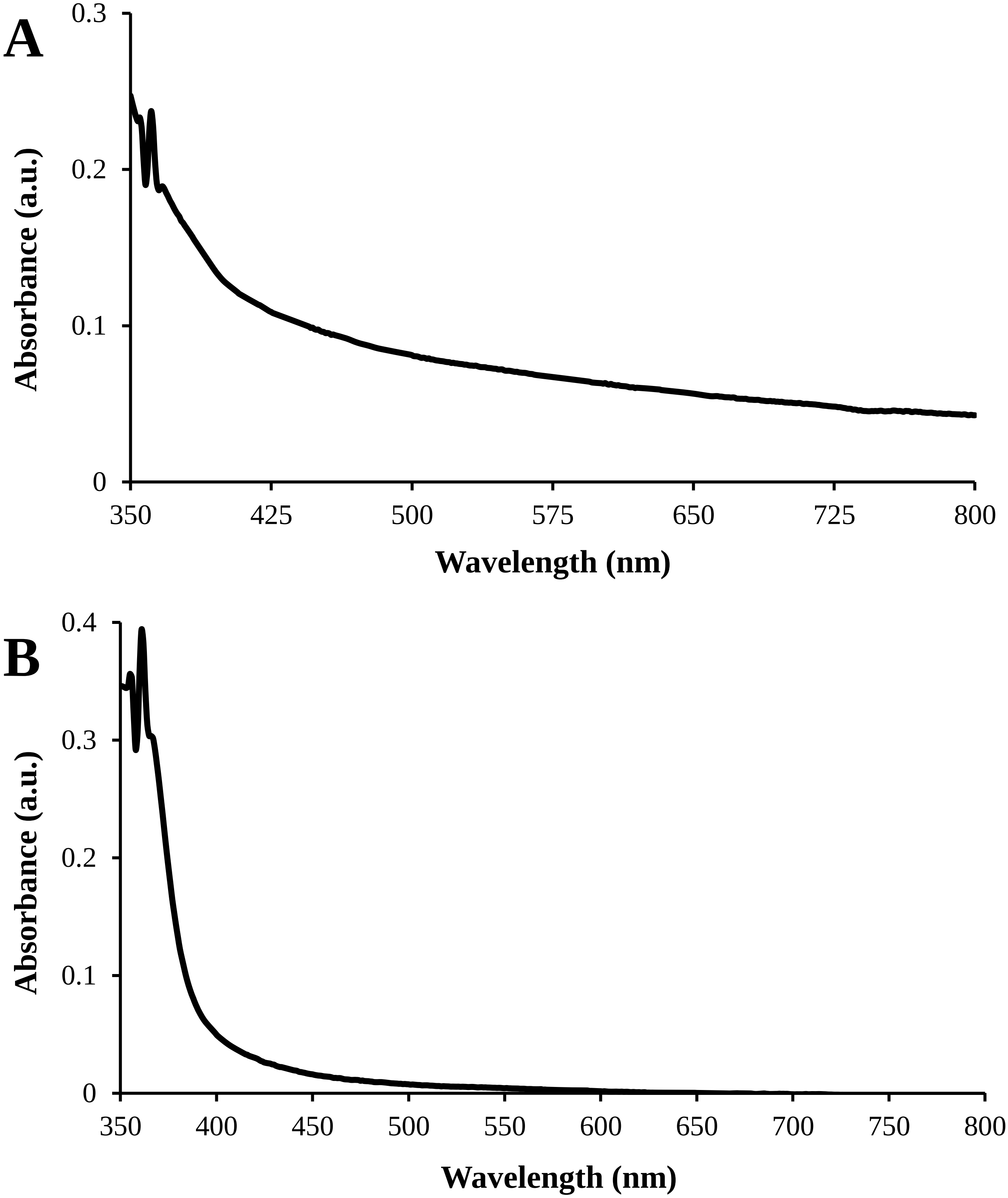

Figure S25. UV-Vis spectra of **(A) thiacloprid** (113 mM) and **(B) clothianidin** (67 mM) in tetrahydrofuran.

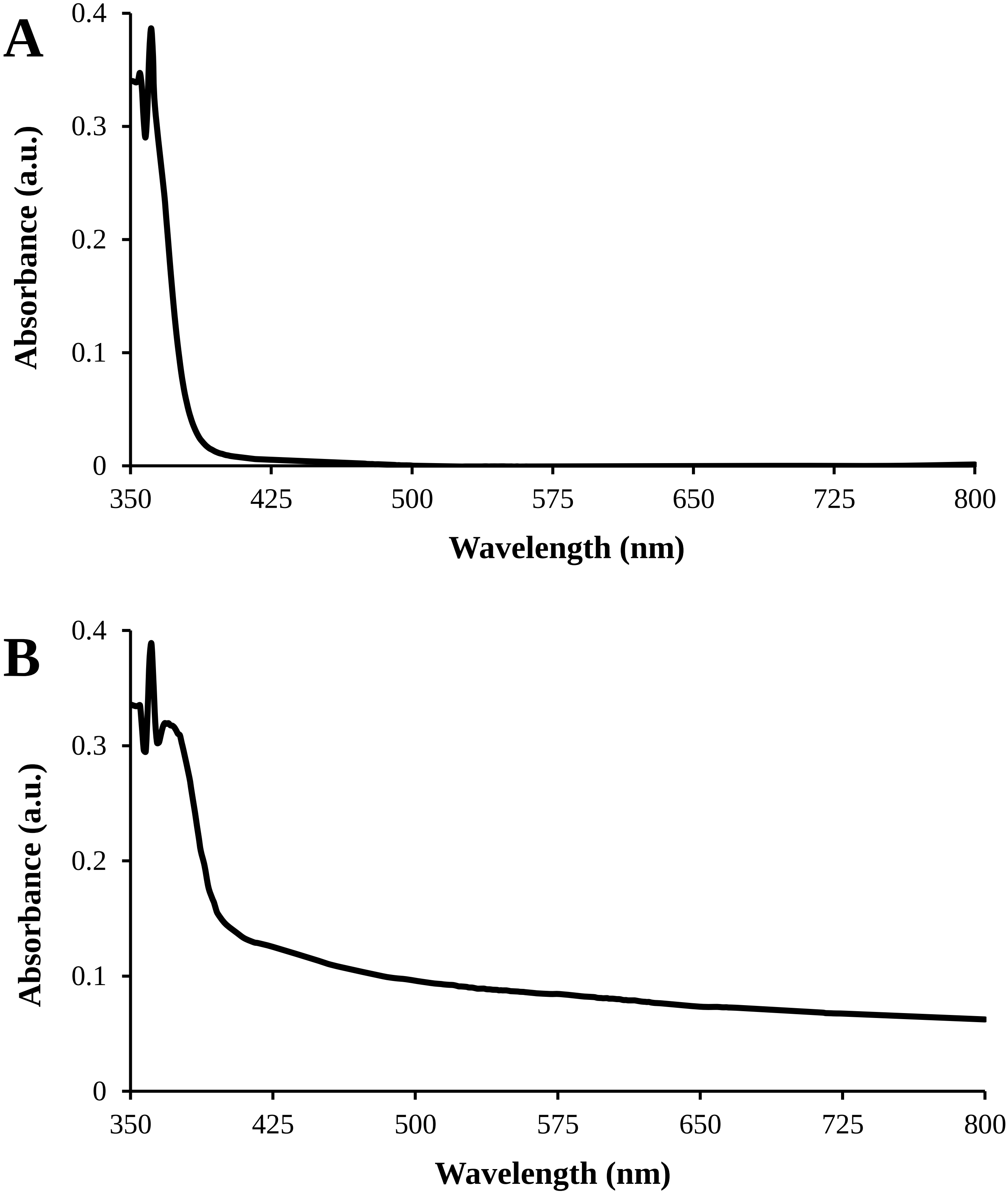

Figure S26. UV-Vis spectra of **(A) imidacloprid** (50 mM) and **(B) thiamethoxam** (70 mM) in tetrahydrofuran.

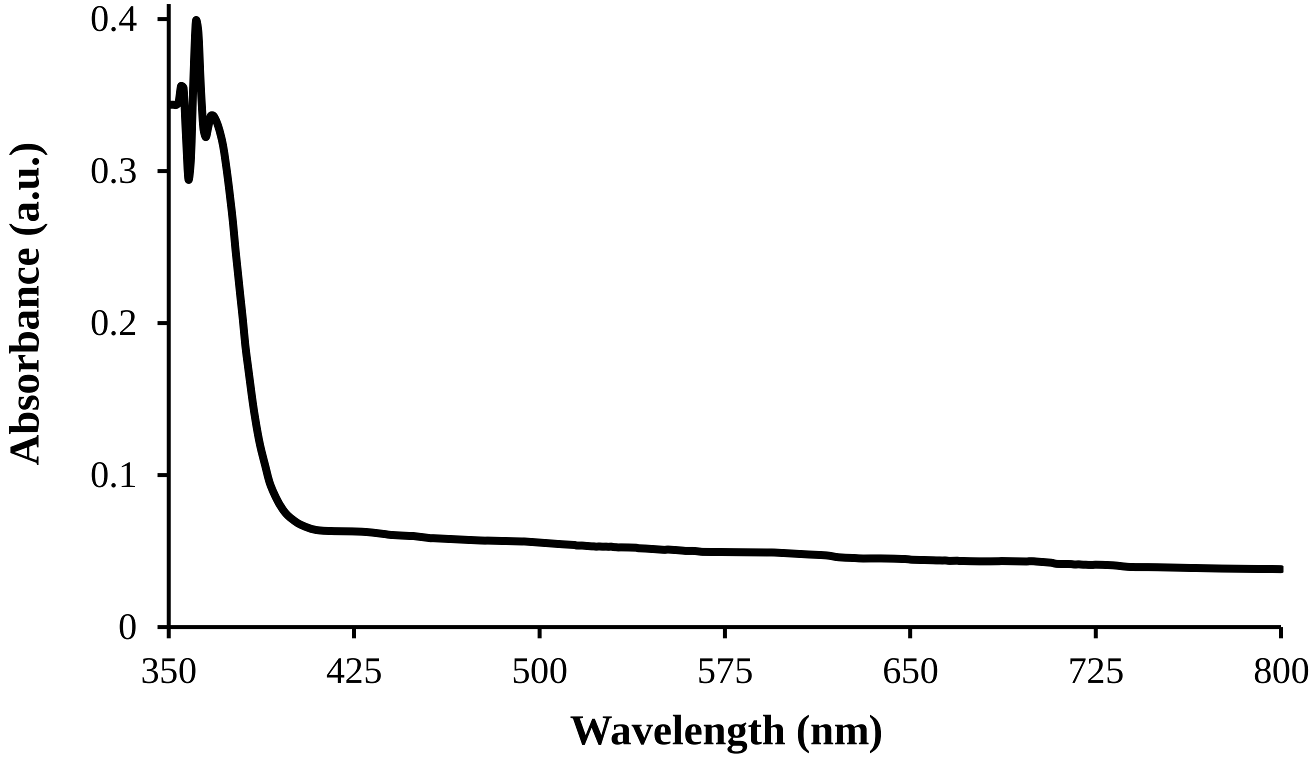

Figure S27. UV-Vis spectrum of **dinotefuran** (75 mM) in tetrahydrofuran.

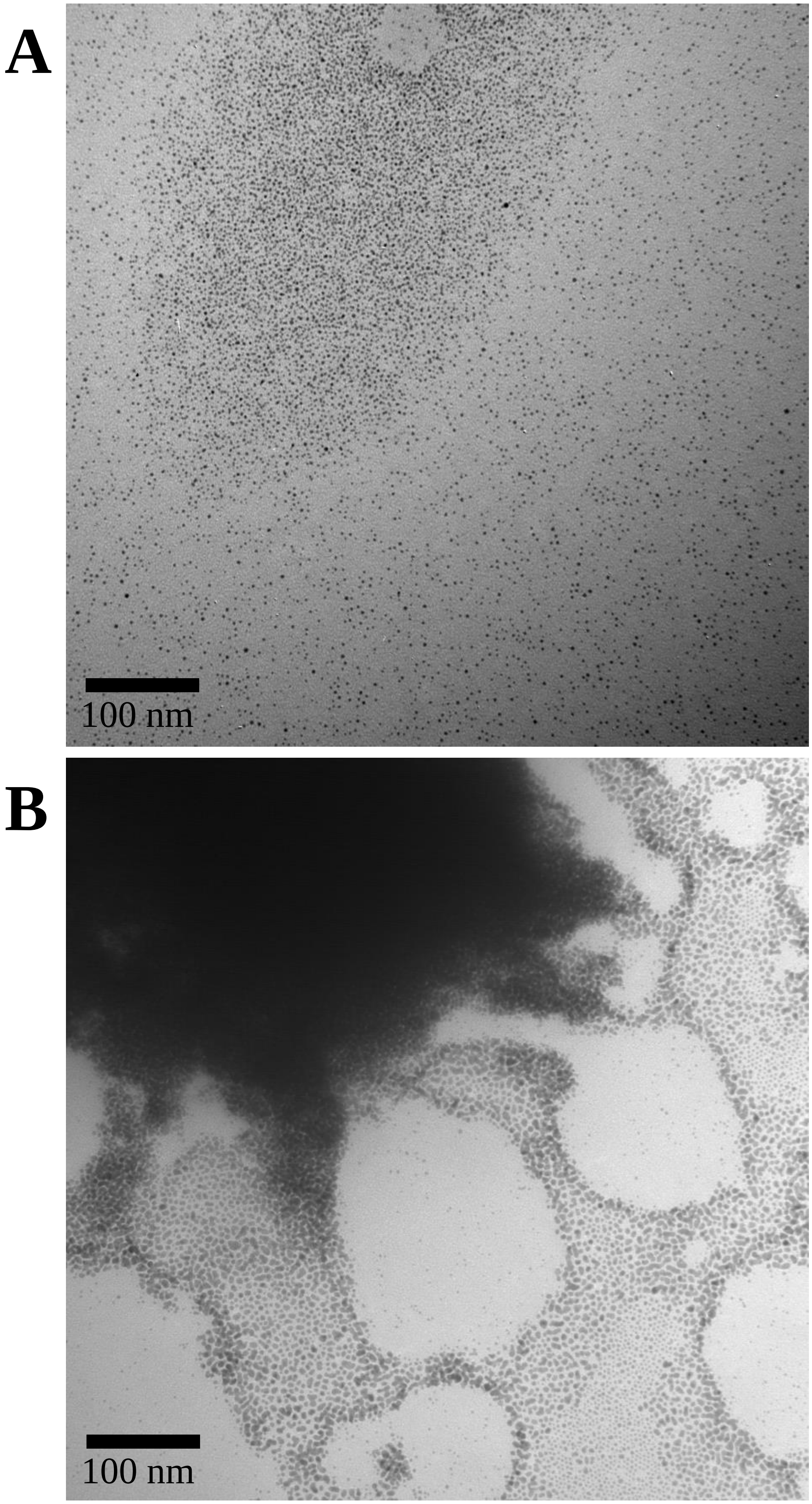

Figure S28. TEM images of *f*-MPCs **(A)** before and **(B)** 2 minutes after the addition of **imidacloprid** (50 mM). Images are representative of multiple sites imaged of each sample.

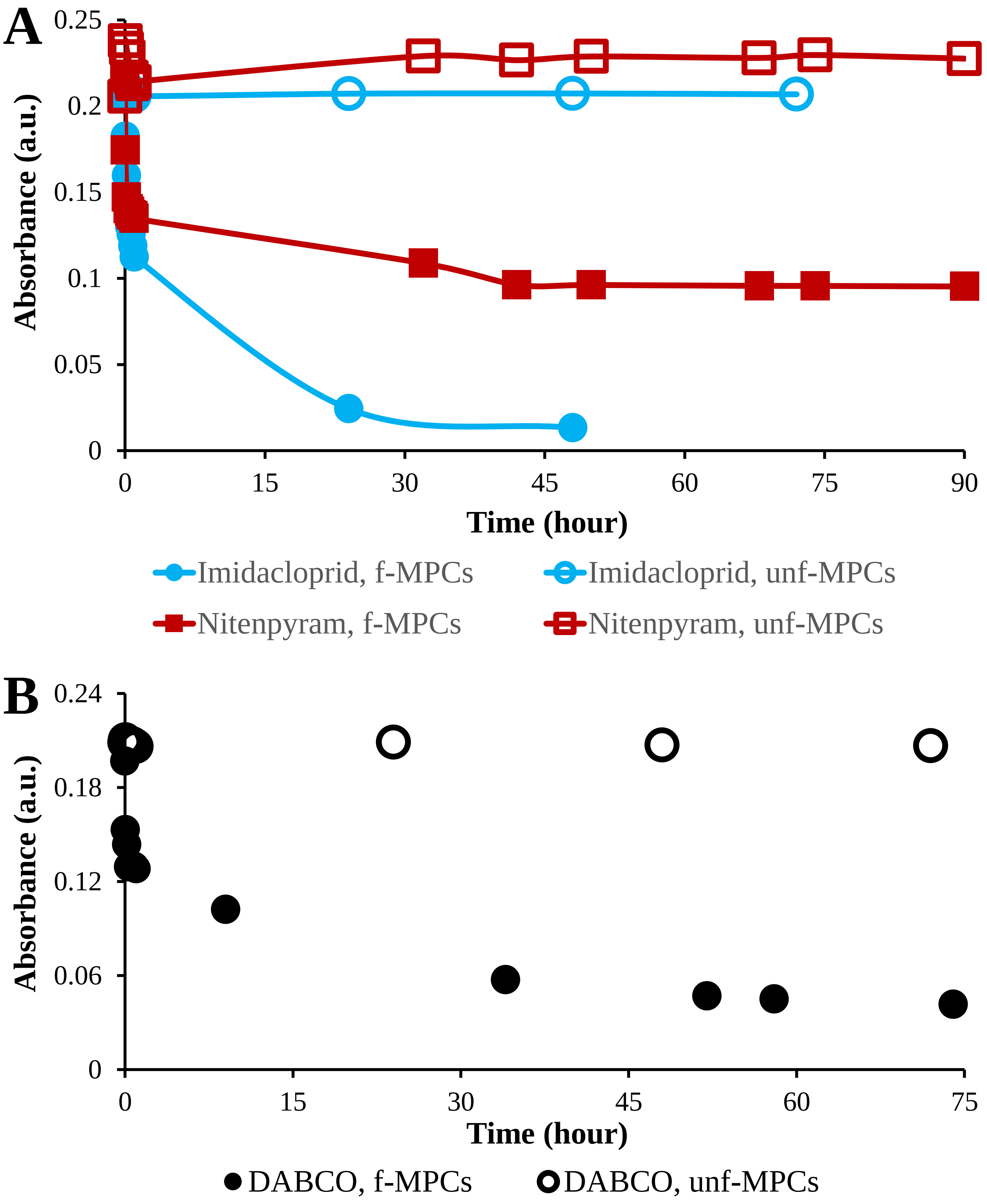

Figure S29. Spectroscopic tracking of $Abs_{@518nm}$ as a function of time of the mixtures of either *f*-MPCs or *unf*-MPCs exposed to **(A)** either **imidacloprid** and **nitenpyram** (longer time-scale analysis) or **(B)** DABCO (control).

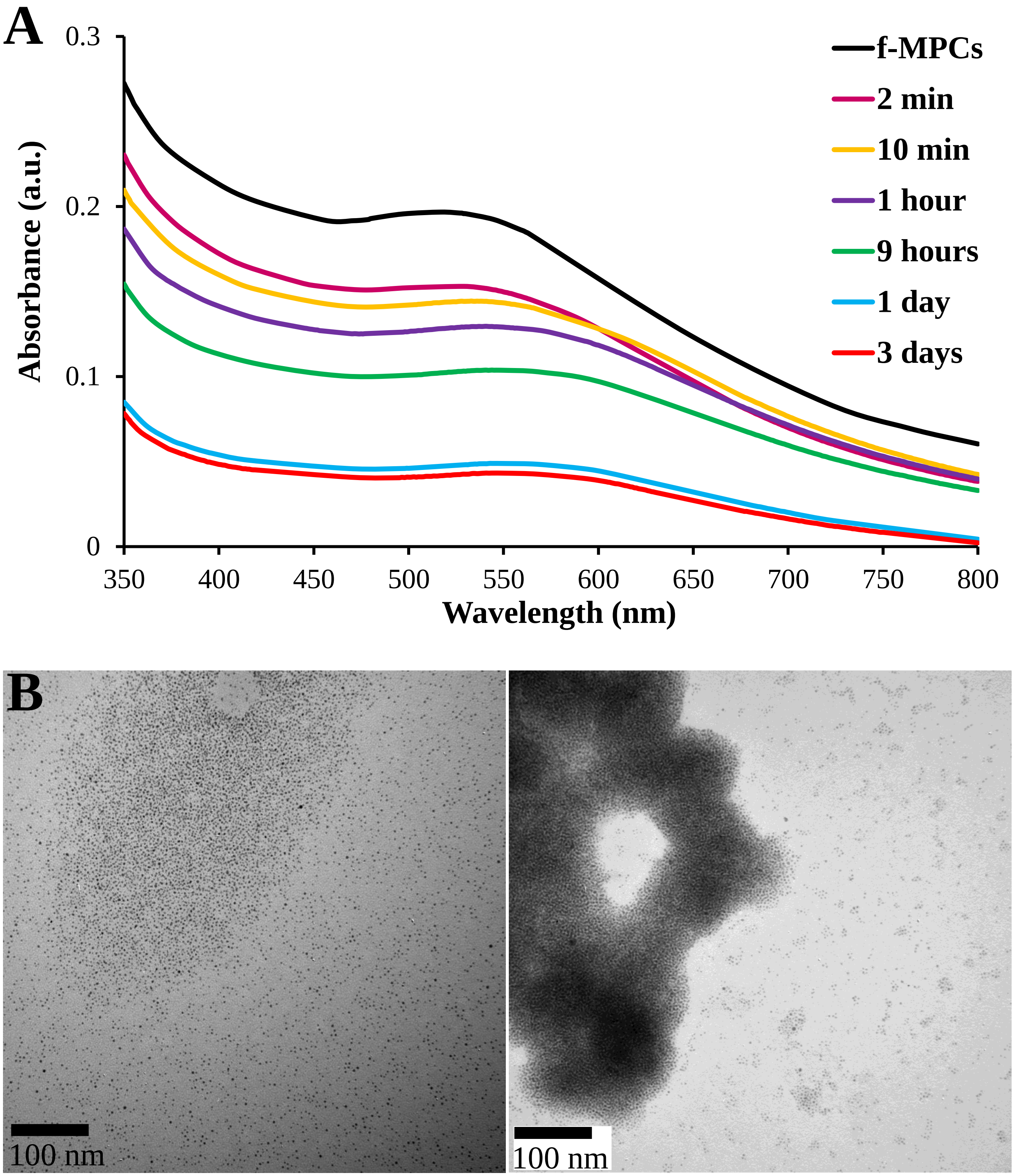

Figure S30. **(A)** UV–Vis spectra of *f*-MPCs in toluene upon exposure to DABCO (1 mM) over time. **(B)** TEM images of *f*-MPCs **(left)** before and **(right)** 5 minutes after DABCO addition.

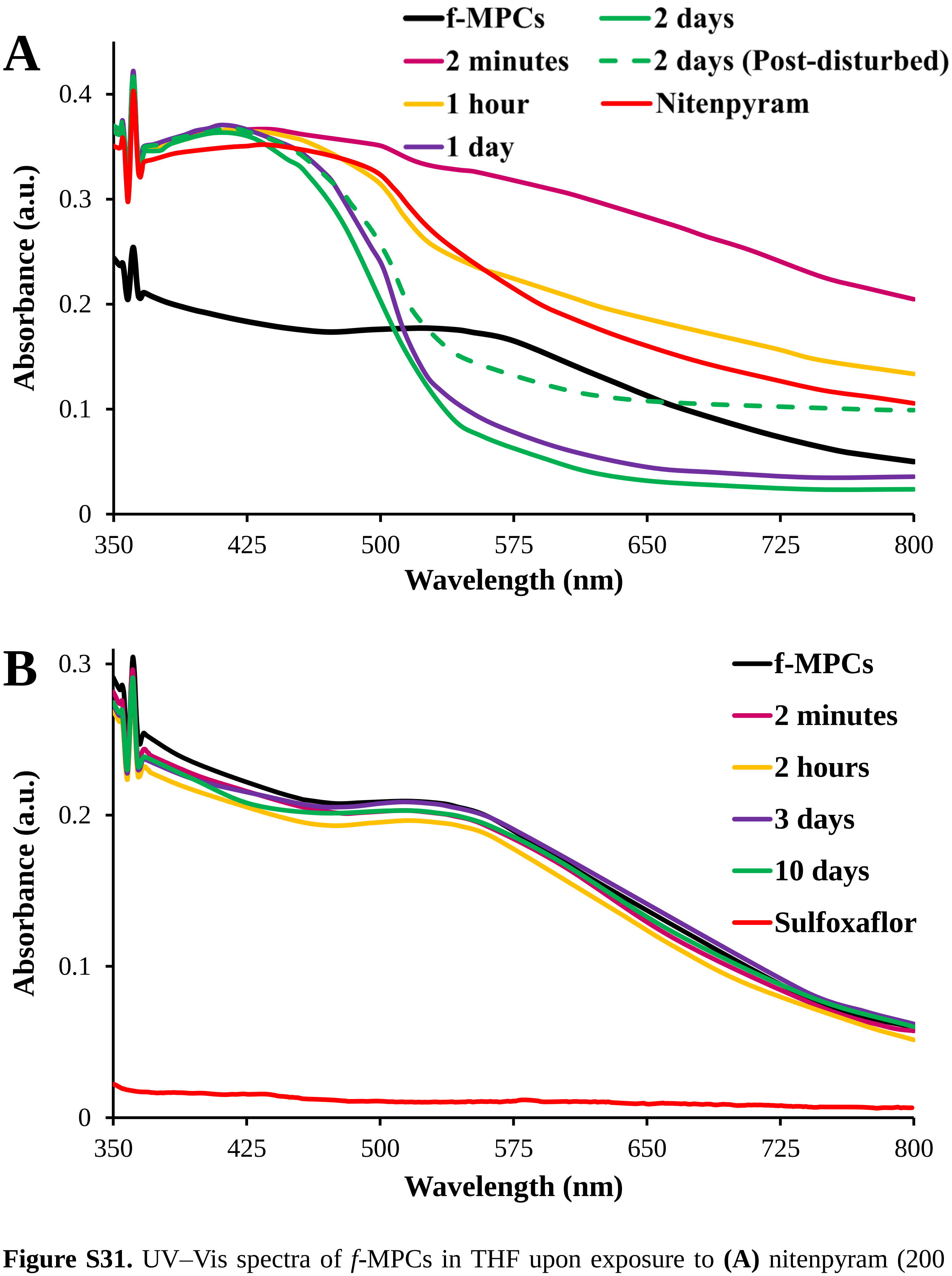

Figure S31. UV–Vis spectra of *f*-MPCs in THF upon exposure to **(A)** nitenpyram (200 mM) and **(B)** sulfoxaflor (72 mM) over time.

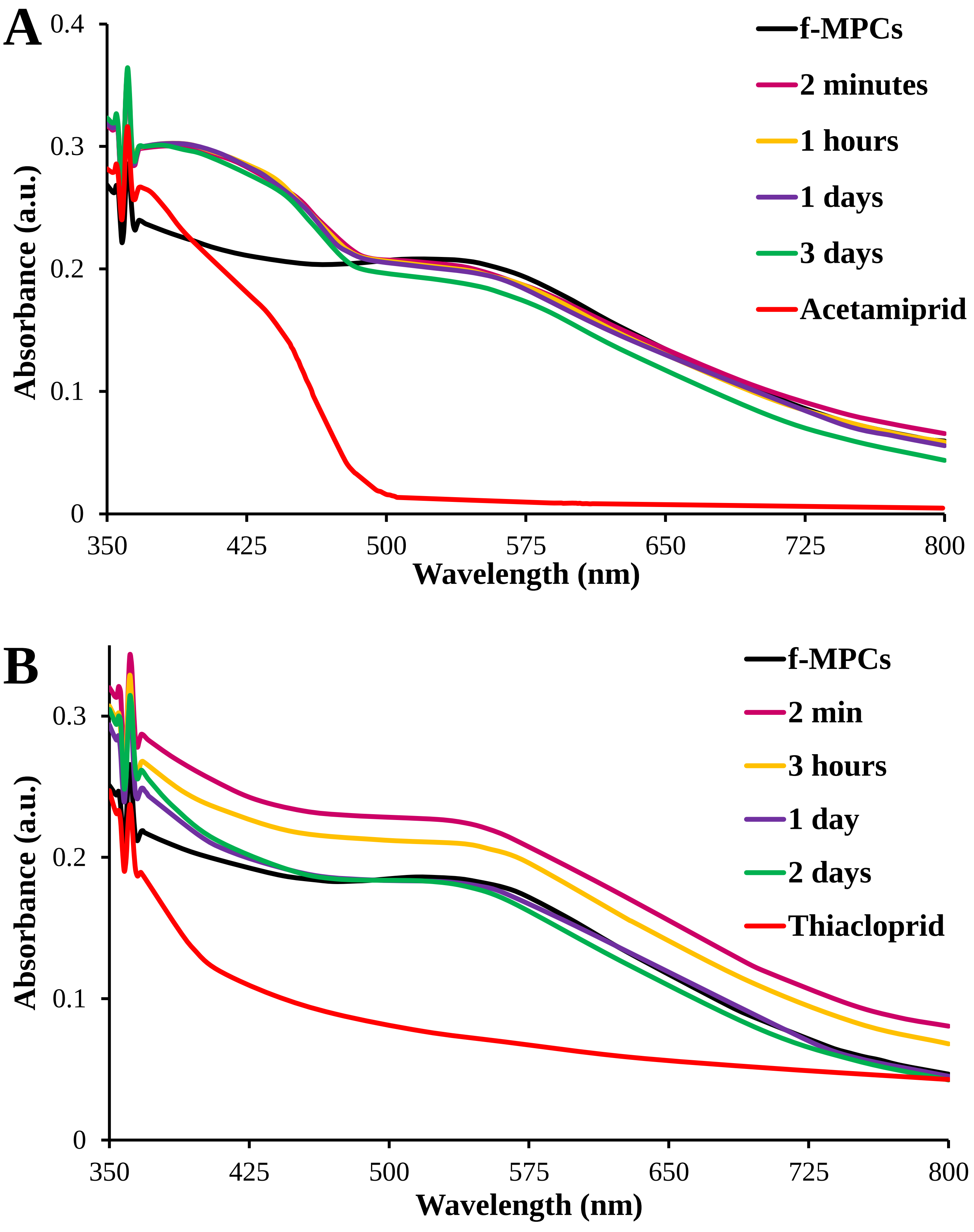

Figure S32. UV–Vis spectra of *f*-MPCs in THF upon exposure to **(A)** acetamiprid (75 mM) and **(B)** thiacloprid (113 mM) over time.

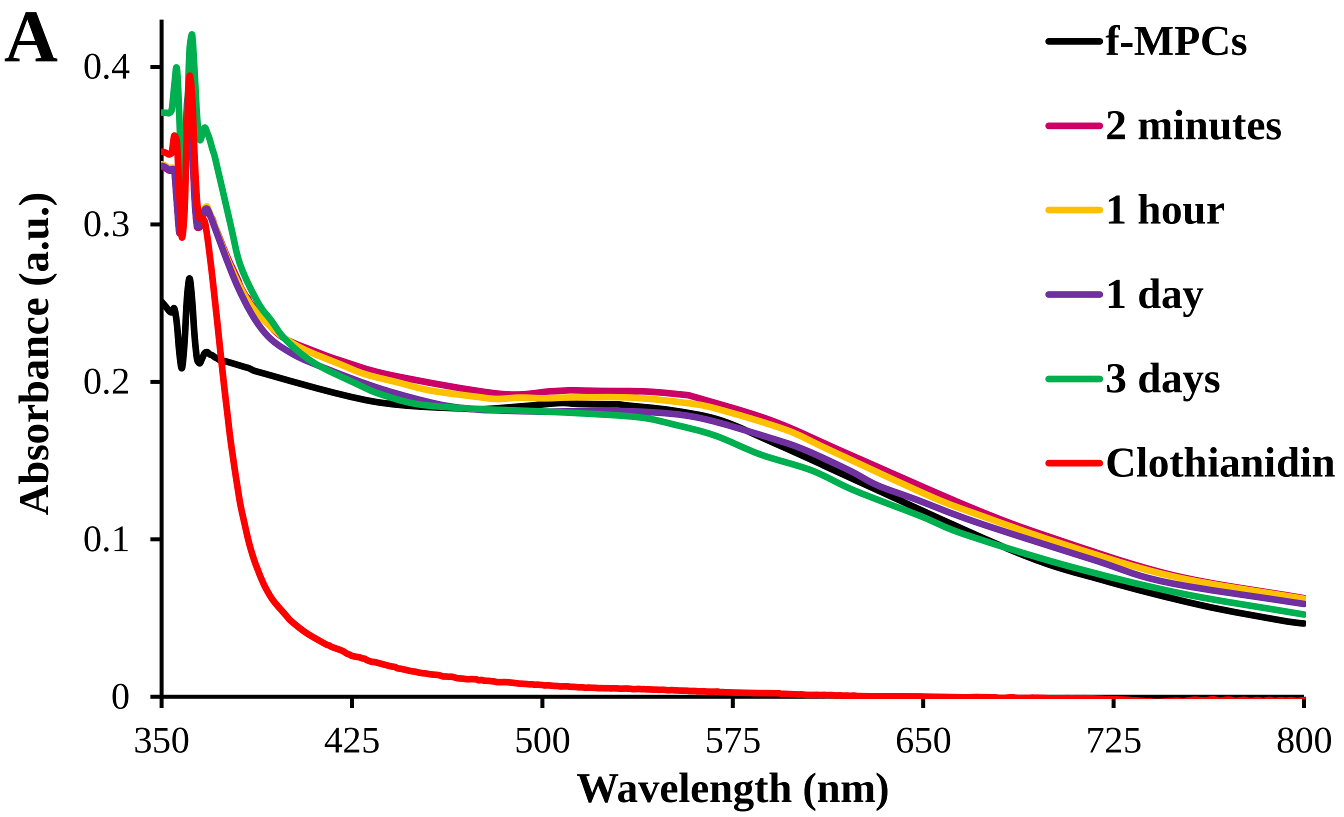

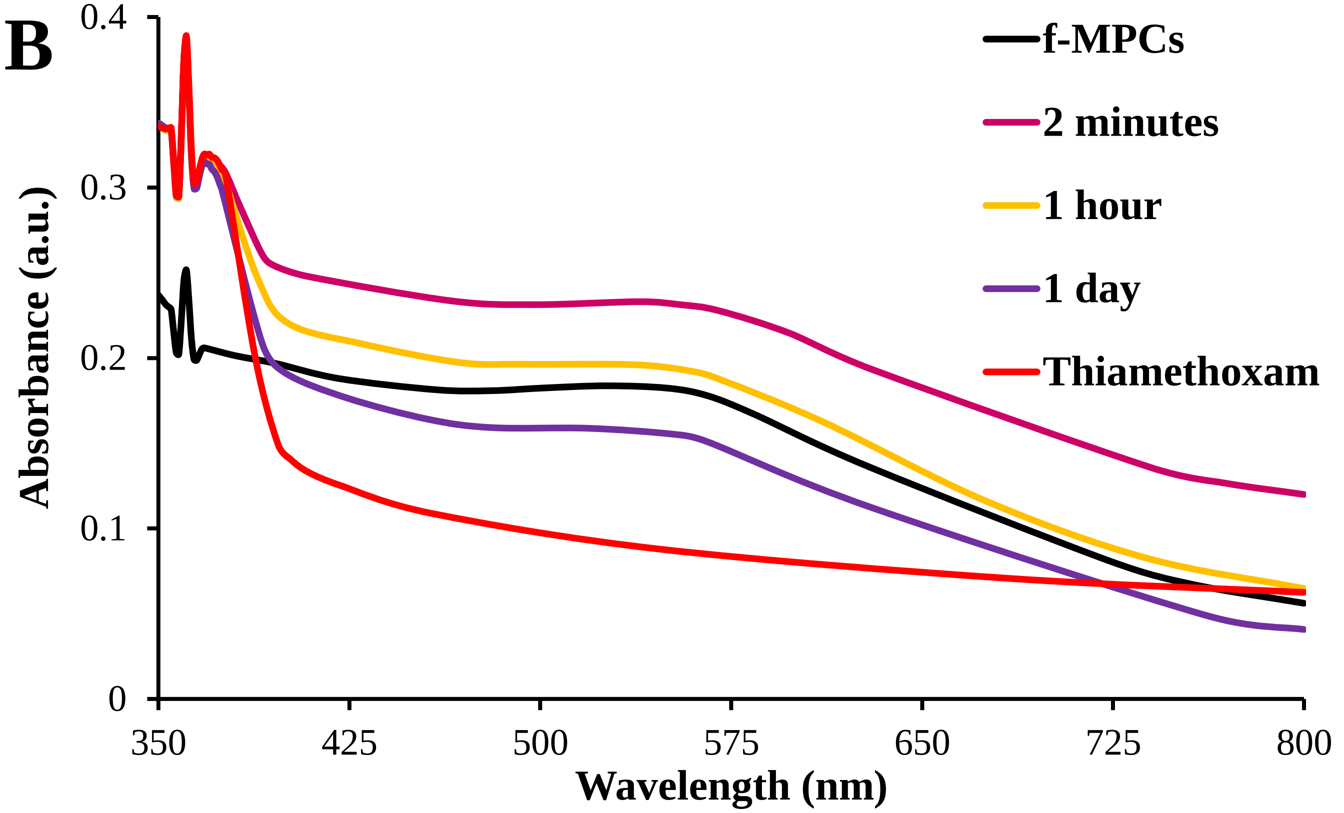

Figure S33. UV–Vis spectra of *f*-MPCs in THF upon exposure to **(A)** clothianidin (67 mM) and **(B)** thiamethoxam (50 mM) over time.

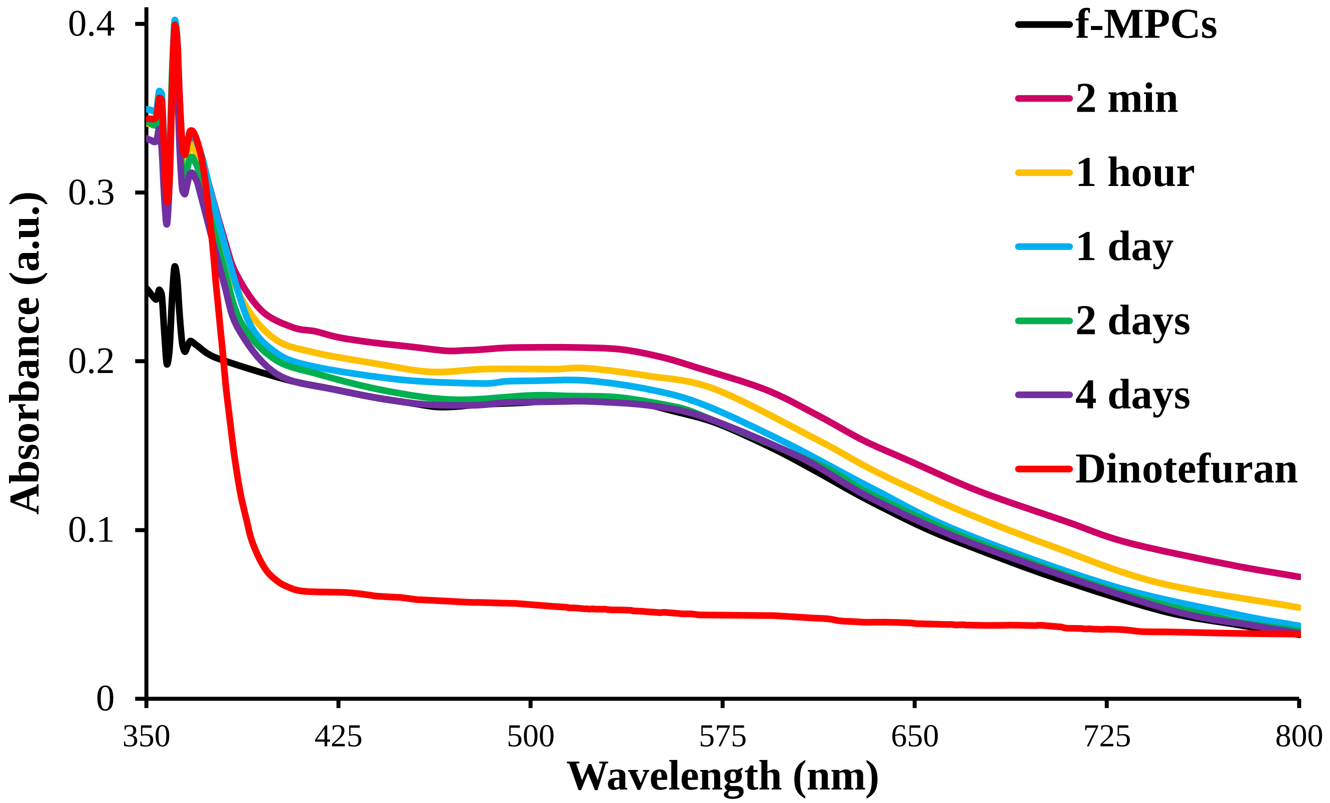

Figure S34. UV–Vis spectra of *f*-MPCs in THF upon exposure to dinotefuran (73 mM) over time.

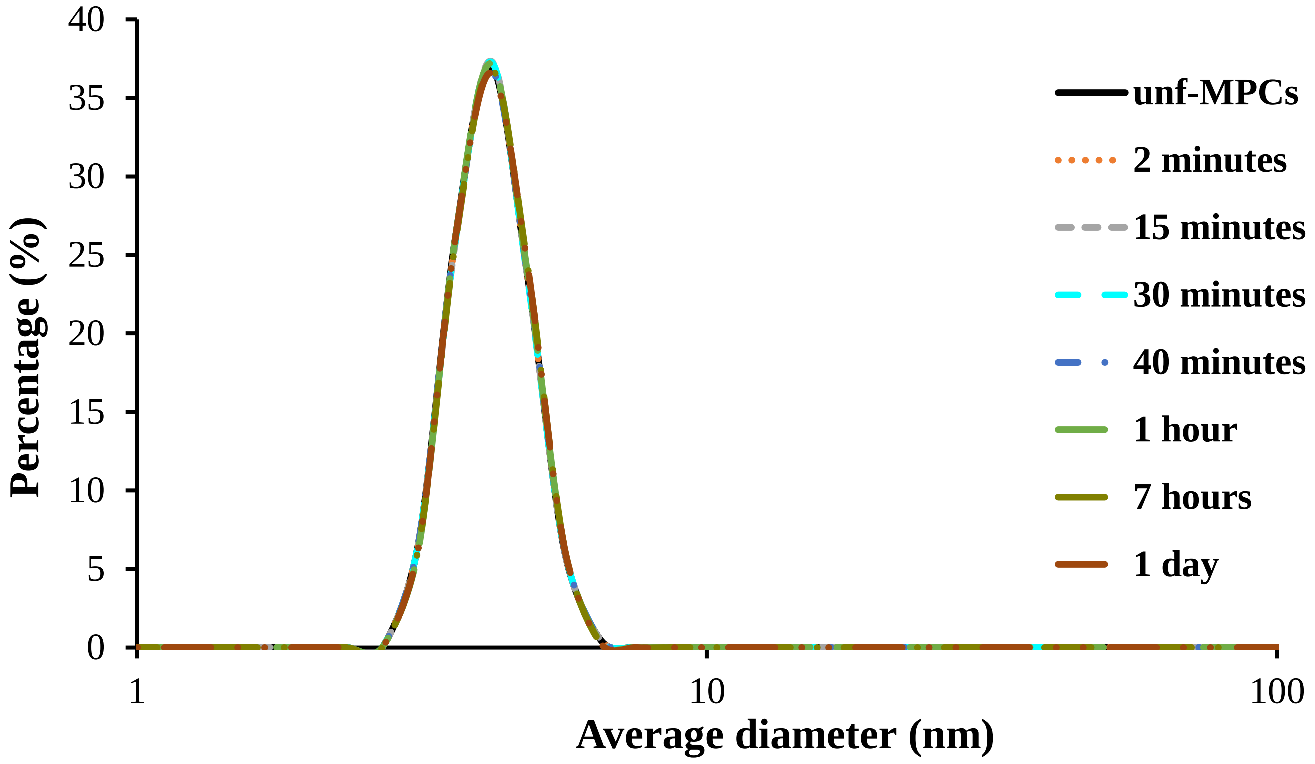

Figure S35. DLS results of *unf*-MPCs in THF ($Abs_{@518}$= 0.20 a.u.) before and after the addition of imidacloprid (12 mM) as a function of time.

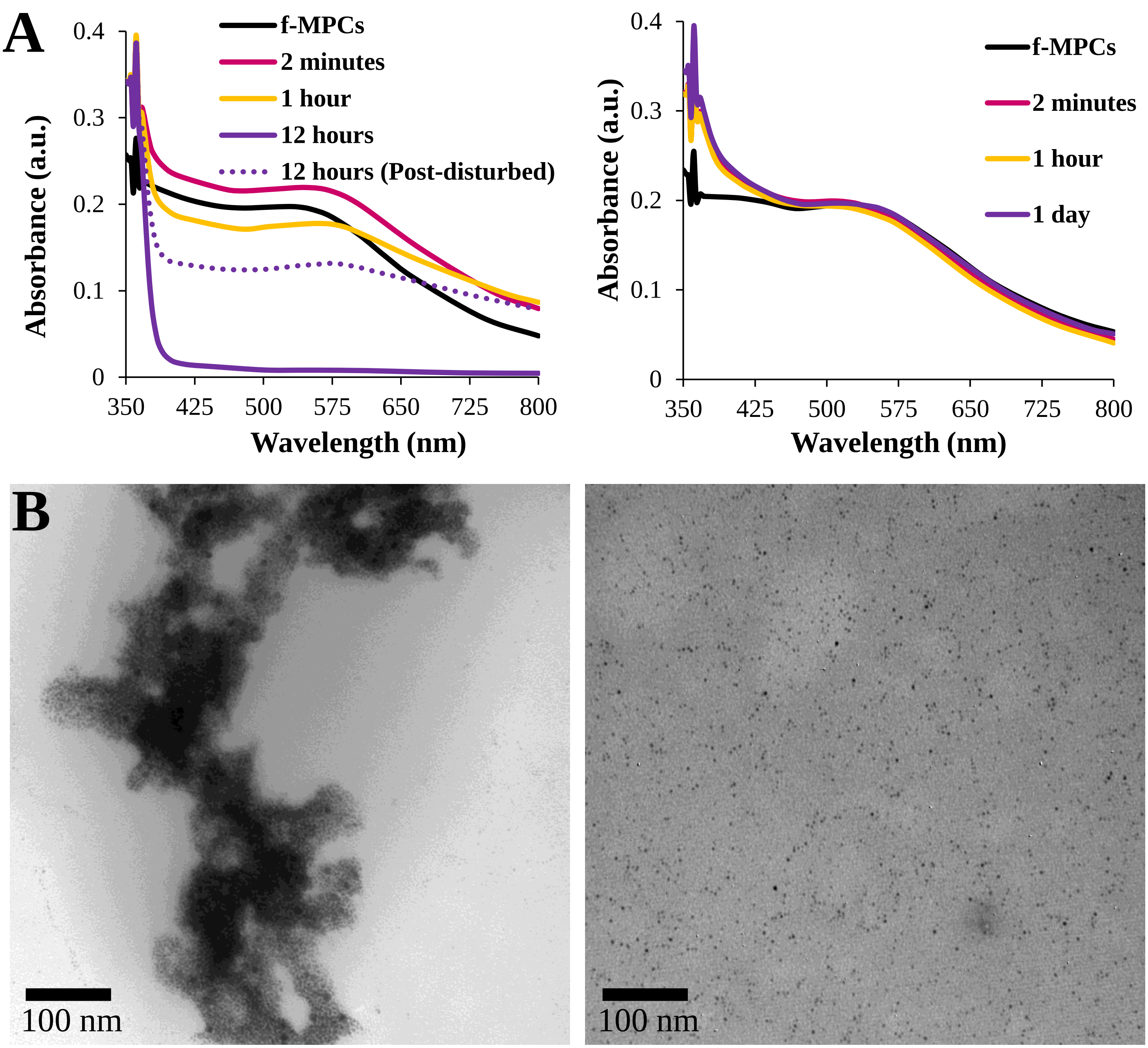

Figure S36. **(A)** UV-Vis spectra and **(B)** corresponding TEM images (at 1 hour) of *f*-MPCs in THF upon exposure to other neonicotinoid compounds (50 mM clothianidin and 50 mM acetamiprid) in the presence (***left***) and absence (***right***) of 50 mM imidacloprid over time.

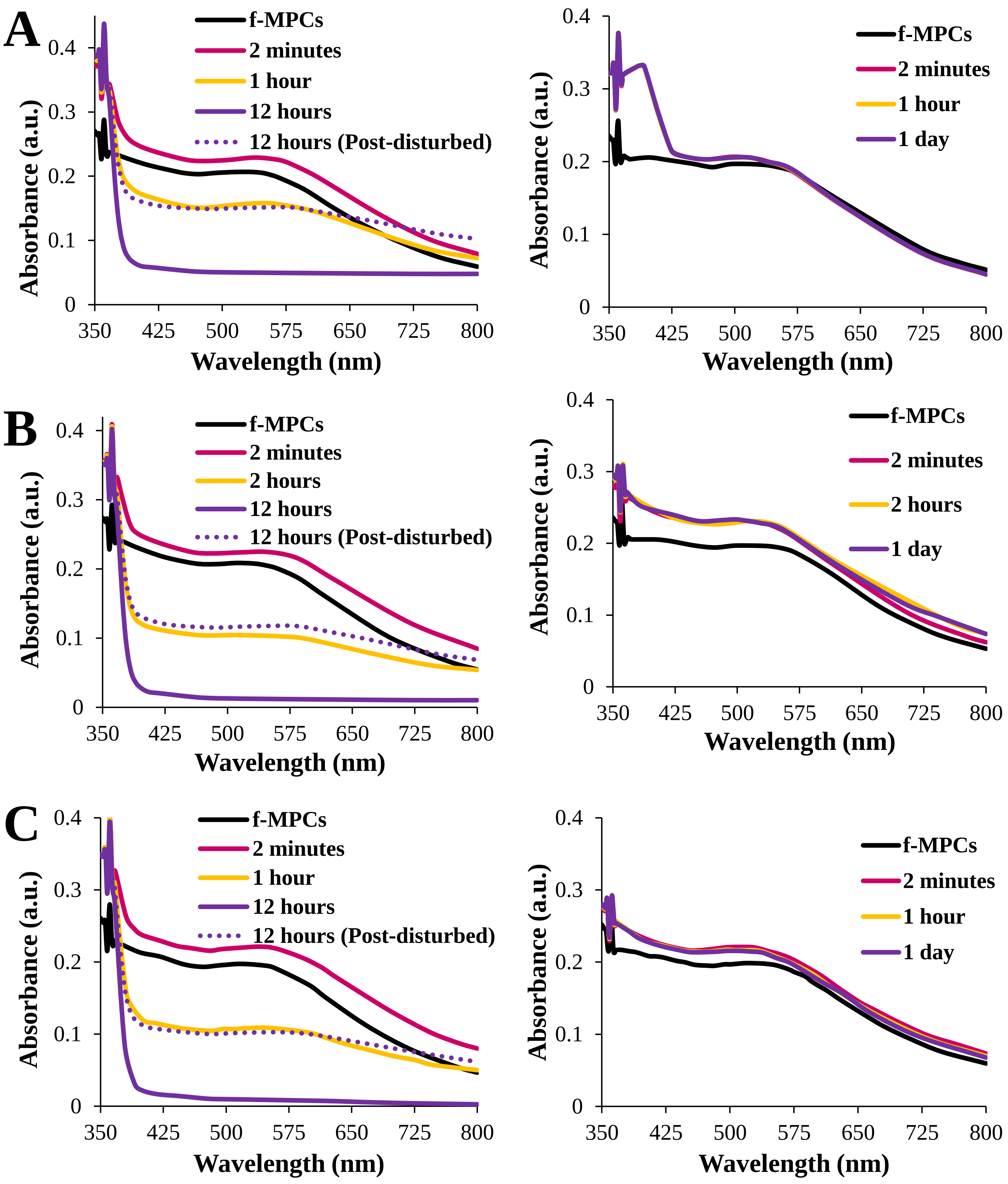

Figure S37. UV-Vis spectra of *f*-MPCs in THF upon exposure to other organophosphate pesticides **(A)** 50 mM parathion, **(B)** 50 mM chlorpyrifos, and **(C)** carbamate pesticide carbaryl (50 mM) in the presence (***left***) and absence (***right***) of 50 mM imidacloprid over time.

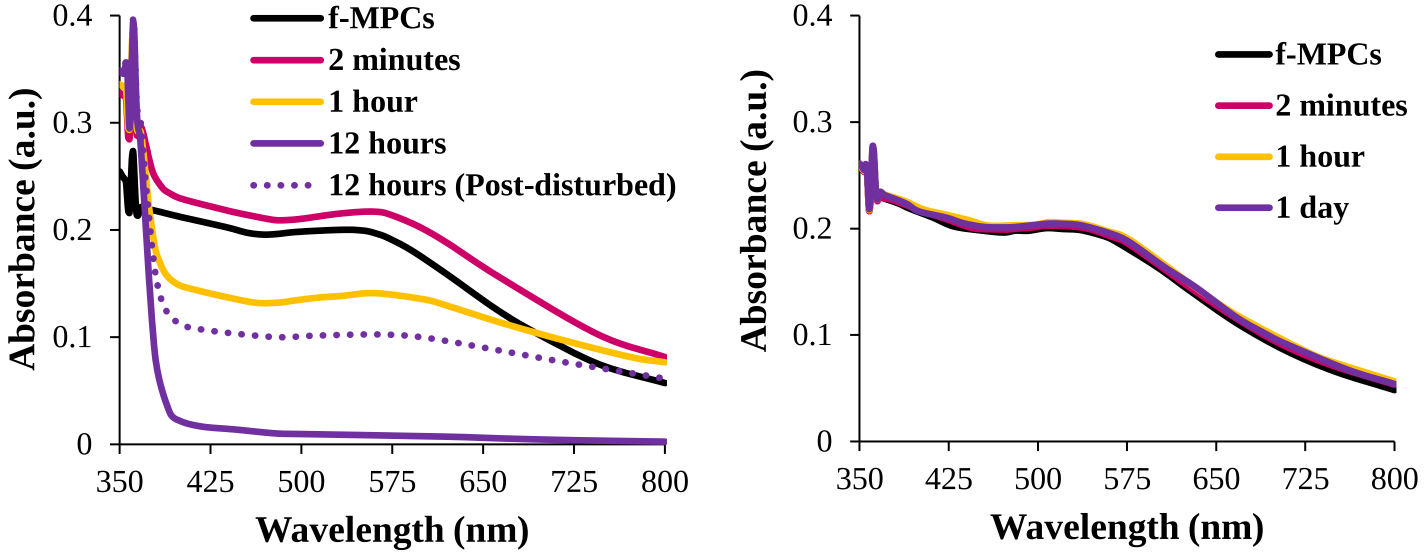

Figure S38. UV-Vis spectra of *f*-MPCs in THF with a common plasticizer found in the environment (50 mM dioctyl phlthalate) in the presence (***left***) and absence (***right***) of 50 mM imidacloprid over time.

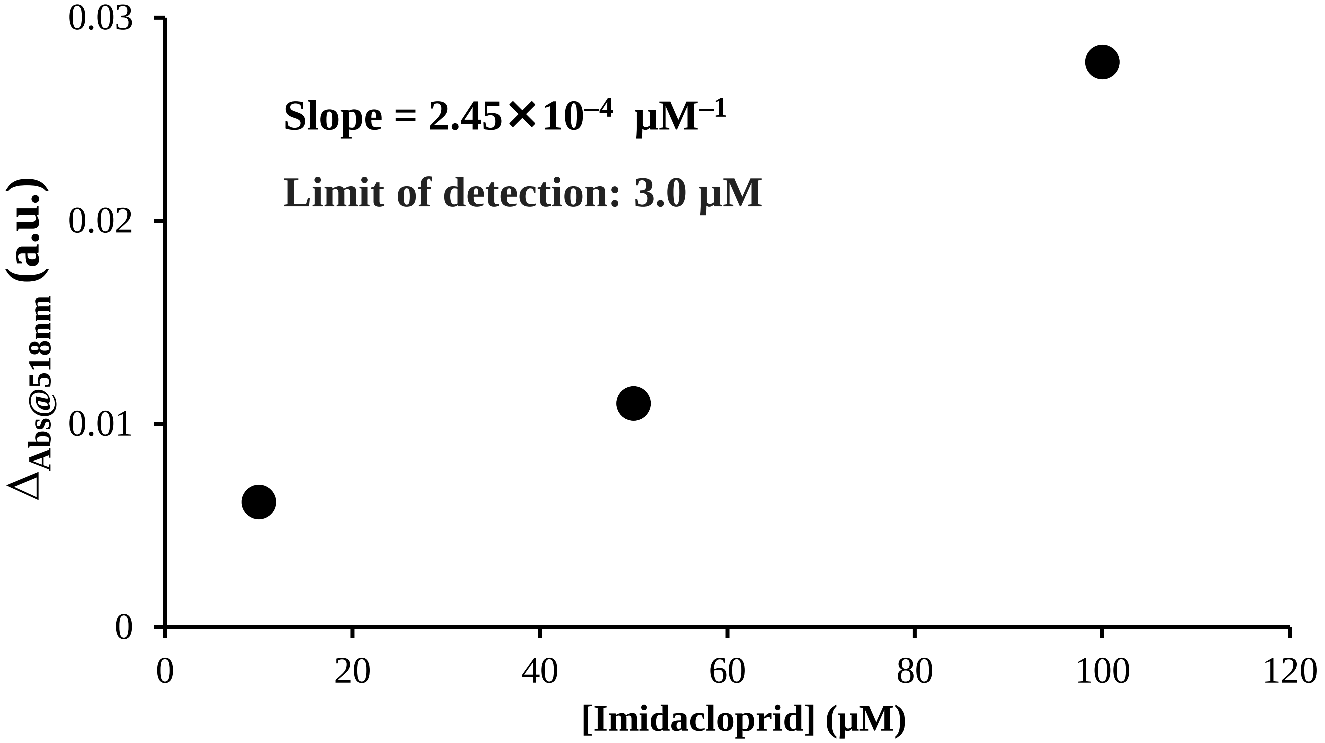

Figure S39. Imidacloprid calibration curve for low concentrations (µM) mixtures with *f*-MPC solutions ($Abs_{@518}$= 0.20 a.u. or ~28 nM) in THF where measurements were collected 1 hour after the addition of imidacloprid. Limit of detection was calculated using the equation $3\sigma_{blank}$ / (slope of calibration curve).

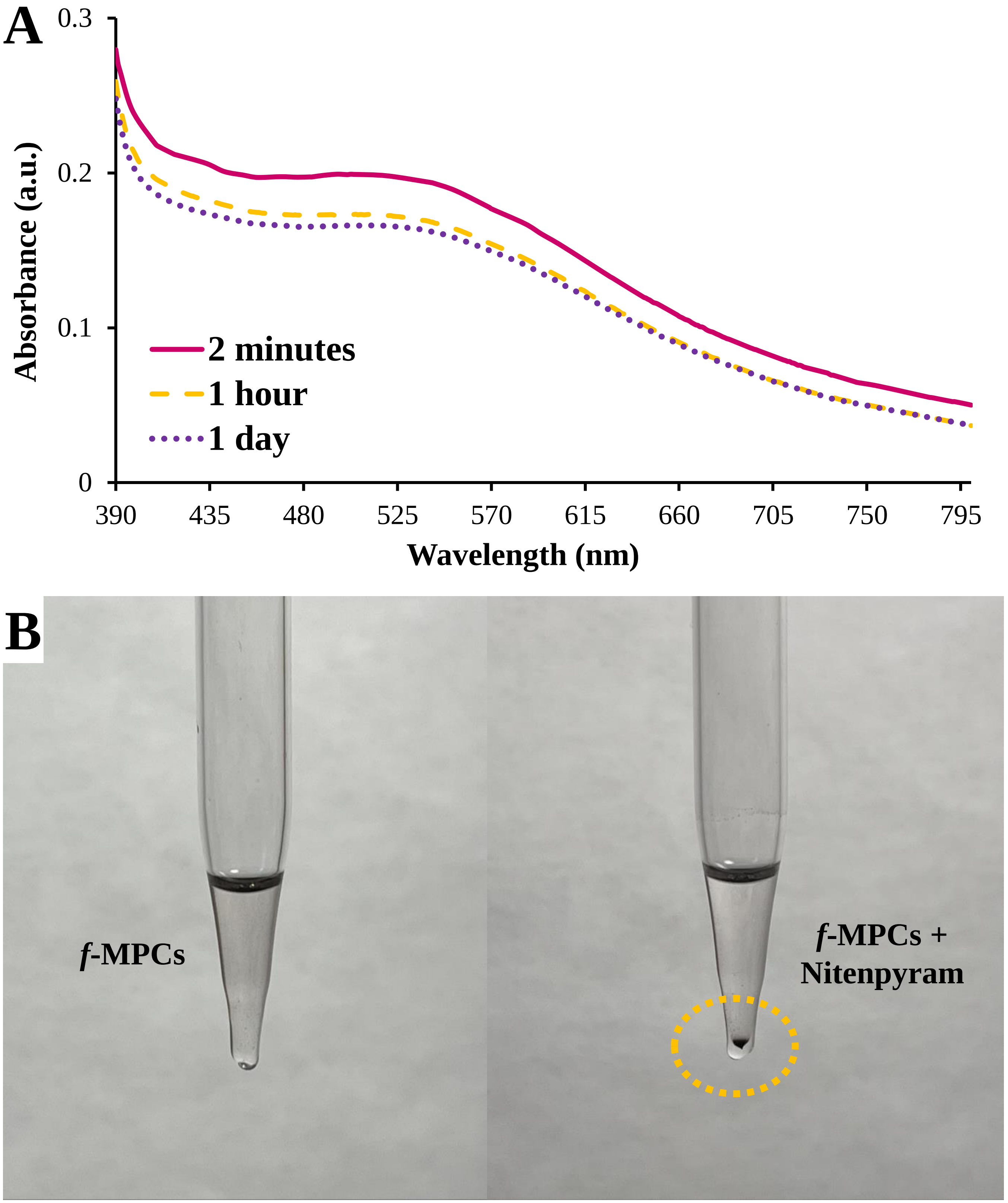

Figure S40. (A) UV–Vis spectra of *f*-MPCs in toluene exposed to 1 μM nitenpyram over time. **(B)** Visual images of 10 μL of *f*-MPC solution in toluene in a sealed Pasteur pipette before (**left**) and 1 hour after (**right**) the addition of 11 μg of nitenpyram.

References

1. Frisch, M. J.; Trucks, G. W.; Schlegel, H. B.; Scuseria, G. E.; Robb, M. A.; Cheeseman, J. R.; Scalmani, G.; Barone, V.; Petersson, G. A.; Nakatsuji, H. *Gaussian 16,* Gaussian Inc.: Wallington, CT, 2016.
2. Zhao, Y.; Truhlar, D. G., The M06 suite of density functionals for main group thermochemistry, thermochemical kinetics, noncovalent interactions, excited states, and transition elements: two new functionals and systematic testing of four M06-class functionals and 12 other functionals. *Theor Chem Acc* **2008,** *120* (1-3), 215-241.
3. Dunning, T. H., Gaussian-Basis Sets for Use in Correlated Molecular Calculations .1. The Atoms Boron through Neon and Hydrogen. *J Chem Phys* **1989,** *90* (2), 1007-1023.
4. Kendall, R. A.; Jr., T. H. D.; Harrison, R. J., Electron affinities of the first-row atoms revisited. Systematic basis sets and wave functions. *The Journal of Chemical Physics* **1992,** *96* (9), 6796-6806.
5. Peterson, K. A.; Shepler, B. C.; Figgen, D.; Stoll, H., On the Spectroscopic and Thermochemical Properties of Clo, Bro, Io, and Their Anions. *J Phys Chem A* **2006**, *110*, 13877-13883.
6. Stoll, H.; Metz, B.; Dolg, M., Relativistic Energy-Consistent Pseudopotentials--Recent Developments. *J Comput Chem* **2002**, *23*, 767-78.
7. Tomasi, J.; Mennucci, B.; Cammi, R., Quantum Mechanical Continuum Solvation Models. *Chem Rev* **2005**, *105*, 2999-3094.
8. Donald, K. J.; Wittmaack, B. K.; Crigger, C., Tuning Sigma-Holes: Charge Redistribution in the Heavy (Group 14) Analogues of Simple and Mixed Halomethanes Can Impose Strong Propensities for Halogen Bonding. *J Phys Chem A* **2010**, *114*, 7213-7222.
9. Donald, K. J.; Tawfik, M., The Weak Helps the Strong: Sigma-Holes and the Stability of Mf4 Center Dot Base Complexes. *J Phys Chem A* **2013**, *117*, 14176-14183.
10. Parker, A. J.; Stewart, J.; Donald, K. J.; Parish, C. A., Halogen Bonding in DNA Base Pairs. *Journal of the American Chemical Society* **2012**, *134*, 5165-5172.
11. Tawfik, M.; Donald, K. J., Halogen Bonding: Unifying Perspectives on Organic and Inorganic Cases. *J Phys Chem A* **2014**, *118*, 10090-10100.
12. Dang, Q. M.; Simpson, J. H.; Parish, C. A.; Leopold, M. C., Evaluating Halogen-Bond Strength as a Function of Molecular Structure Using Nuclear Magnetic Resonance Spectroscopy and Computational Analysis. *J Phys Chem A* **2021,** *125* (42), 9377-9393.
